# Supplementary material for: SLC26A11 is an atypical solute carrier with dual transport-channel function mediating lysosomal sulfate transport
Source: Nat Commun. 2026 Jul 27;17:7407. doi: 10.1038/s41467-026-75749-4 (PMC13408680; doi:10.1038/s41467-026-75749-4)
Supplement: Supplementary file 1 — Supplementary Information [file 41467_2026_75749_MOESM1_ESM.pdf]

SLC26A11 is an atypical solute carrier with dual transport-channel function mediating lysosomal sulfate transport

Benedikt T. Kuhn *et alia*

Supplementary Information

- Supplementary Tables 1 – 3
- Supplementary Figures 1 – 20

Supplementary Table 1: Buffer conditions used in proteoliposome transport assays with variable component highlighted in bold. Hepes/Mes buffer stocks were pH adjusted using potassium hydroxide if not state differently. All gluconate stocks were pH adjusted using gluconic acid.

figure panel	internal buffers	external buffers	replicates
1A	20 mM Hepes, 20 mM Mes<br>50 mM potassium<br>2 mM magnesium gluconate<br>**50 mM chloride**<br>pH 7.5	20 mM Hepes, 20 mM Mes<br>50 µM potassium sulfate (2 µCi/mL)<br>50 mM potassium gluconate<br>2 mM magnesium gluconate<br>pH 5.0	n = 3
	20 mM Hepes, 20 mM Mes<br>50 mM potassium<br>2 mM magnesium gluconate<br>**50 mM gluconate**<br>pH 7.5		
1B/4F	20 mM Hepes, 20 mM Mes<br>50 mM potassium chloride<br>2 mM magnesium gluconate<br>**pH 5.0**	20 mM Hepes, 20 mM Mes<br>50 µM potassium sulfate (2 µCi/mL)<br>50 mM potassium gluconate<br>2 mM magnesium gluconate<br>**pH 5.0 to pH 7.5 (0.5 pH steps)**	n = 4 for pH5/pH5,<br>n = 3 for all remaining conditions
	20 mM Hepes, 20 mM Mes<br>50 mM potassium chloride<br>2 mM magnesium gluconate<br>**pH 7.5**		
S2A	20 mM Hepes, 20 mM Mes (KOH)<br>50 mM potassium chloride<br>2 mM magnesium gluconate<br>pH 7.5	20 mM Hepes, 20 mM Mes (**KOH**)<br>50 µM potassium sulfate (2 µCi/mL)<br>**50 mM potassium gluconate**<br>2 mM magnesium gluconate<br>pH 5.0	n = 4
		20 mM Hepes, 20 mM Mes (**NaOH**)<br>50 µM potassium sulfate (2 µCi/mL)<br>**50 mM sodium gluconate**<br>2 mM magnesium gluconate<br>pH 5.0	
1C	20 mM Hepes, 20 mM Mes<br>50 mM potassium chloride<br>2 mM magnesium gluconate<br>pH 7.5	20 mM Hepes, 20 mM Mes<br>**potassium sulfate (2 µCi/mL)**<br>**(6.25, 12.5, 25, 50 or 100 µM)**<br>50 mM potassium gluconate<br>2 mM magnesium gluconate<br>pH 5.0	n = 3
		20 mM Hepes, 20 mM Mes<br>**potassium sulfate (10 µCi/mL)**<br>**(200, 400 or 600 µM)**<br>50 mM potassium gluconate<br>2 mM magnesium gluconate<br>pH 5.0	

figure panel	internal buffers	external buffers	replicates
1D	20 mM Hepes, 20 mM Mes<br>2 mM magnesium gluconate<br>**50 mM potassium chloride**<br>pH 7.5	20 mM Hepes, 20 mM Mes<br>50 µM potassium sulfate (2 µCi/mL)<br>2 mM magnesium gluconate<br>**50 mM potassium chloride**<br>pH 5.0	n = 4
	20 mM Hepes, 20 mM Mes<br>2 mM magnesium gluconate<br>**50 mM potassium gluconate**<br>pH 7.5	20 mM Hepes, 20 mM Mes<br>50 µM potassium sulfate (2 µCi/mL)<br>2 mM magnesium gluconate<br>**50 mM potassium gluconate**<br>pH 5.0	
1E	20 mM Hepes, 20 mM Mes<br>50 mM potassium chloride<br>2 mM magnesium gluconate<br>pH 7.5	20 mM Hepes, 20 mM Mes<br>50 µM potassium sulfate (2 µCi/mL)<br>45 mM potassium gluconate<br>2 mM magnesium gluconate<br>5 mM **tested anions (sodium salt)**<br>pH 5.0	n = 4
1F	20 mM Hepes, 20 mM Mes<br>42 mM potassium gluconate<br>8 mM potassium chloride<br>2 mM magnesium gluconate<br>pH 7.5	20 mM Hepes, 20 mM Mes<br>50 µM potassium sulfate (2 µCi/mL)<br>50 mM potassium gluconate<br>2 mM magnesium gluconate<br>pH 5.0<br><br>21-fold counterflow buffer:<br>20 mM Hepes, 20 mM Mes<br>50 mM sodium gluconate<br>2 mM magnesium gluconate<br>pH 5.0<br>2.1 mM CCCP<br>**105 mM tested anions (sodium salt)**	n = 3
S2B	20 mM Hepes, 20 mM Mes (NaOH)<br>**50 mM potassium chloride**<br>2 mM magnesium gluconate<br>pH 7.5	20 mM Hepes, 20 mM Mes (NaOH)<br>50 µM potassium sulfate (2 µCi/mL)<br>**50 mM potassium gluconate**<br>2 mM magnesium gluconate<br>100 nM valinomycin<br>pH 5.0	n = 4
	20 mM Hepes, 20 mM Mes (NaOH)<br>**5 mM potassium chloride**<br>45 mM sodium chloride<br>2 mM magnesium gluconate<br>pH 7.5	20 mM Hepes, 20 mM Mes (NaOH)<br>50 µM potassium sulfate (2 µCi/mL)<br>**5 mM potassium gluconate**<br>45 mM sodium gluconate<br>2 mM magnesium gluconate<br>100 nM valinomycin<br>pH 5.0	
		20 mM Hepes, 20 mM Mes (NaOH)<br>50 µM potassium sulfate (2 µCi/mL)<br>**50 mM potassium gluconate**<br>2 mM magnesium gluconate<br>pH 5.0	
		20 mM Hepes, 20 mM Mes (NaOH)<br>50 µM potassium sulfate (2 µCi/mL)<br>**5 mM potassium gluconate**<br>45 mM sodium gluconate<br>2 mM magnesium gluconate<br>pH 5.0	

Supplementary Table 2: Cryo-EM datacollection, refinement and validation statistics

	$SLC26A11^{\Delta C}$-Nb4	$SLC26A11^{\Delta C}$-Nb11
**Data collection and processing**		
EM reconstruction method	SINGLE PARTICLE	SINGLE PARTICLE
Imposed symmetry	POINT, C2	POINT, C2
Number of particles used	210771	48803
Resolution determination method	FSC 0.143 CUT-OFF	FSC 0.143 CUT-OFF
CTF correction method	PHASE FLIPPING AND AMPLITUDE CORRECTION	PHASE FLIPPING AND AMPLITUDE CORRECTION
Microscope	TFS KRIOS	TFS KRIOS
Voltage (kV)	300	300
Electron dose ($e^-/Å^2$)	70	79
Minimum defocus (nm)	600	1000
Maximum defocus (nm)	1600	1400
Magnification	130000	75000
Image detector	FEI FALCON IV (4k x 4k)	FEI FALCON III (4k x 4k)
Map size (Å)	283.8, 283.8, 283.8	212.70001, 212.70001, 212.70001
Map angles (°)	90.0, 90.0, 90.0	90.0, 90.0, 90.0
Pixel spacing (Å)	0.946, 0.946, 0.946	1.0635, 1.0635, 1.0635
**Refinement**		
Model resolution (Å)	2.8	3.2
Model composition		
Nonhydrogen atoms	10176	10316
Protein residues	1316	1344
Ligands	PEE, CL	NAG, CL
B factors ($Å^2$)		
Protein	53.04	80.32
Ligand	70.75	135.48
R.m.s. deviations		
Bond lengths (Å)	0.005	0.004
Bond angles (°)	1.024	1.023
Validation		
CC (mask)	0.8	0.77
Q-score	0.518	0.521
MolProbity score	1.17	1.1
Clashscore	3.83	3.11
Poor rotamers (%)	0.18	0.09
Ramachandran favored (%)	98.46	98.43
Ramachandran allowed (%)	1.54	1.57
Ramachandran disallowed (%)	0	0

Supplementary Table 3: Molecular Dynamics simulations checklist

**Reliability and reproducibility checklist for molecular dynamics simulations**<br>***All boxes must be marked YES by acceptance unless "Response not needed if No".**	**Yes**	**No**	**Response**<br>**(Please state where this information can be found in the text)**
**1. Convergence of simulations and analysis**			
1a. Is an evaluation presented in the text to show that the property being measured has equilibrated in the simulations (*e.g.* time-course analysis)?	☒	☐	The quintessential property being observed in the MD simulations are the kinetic/equilibrium rate constants (Kon, Koff, Kd), which are calculated from transitions of hundreds of spontaneous (un)binding events, thus means are not dependent on convergence/time averaging. This is stated in the methods.
1b. Then, is it described in the text how simulations are split into equilibration and production runs and how much data were analyzed from production runs?	☒	☐	Stated in the methods.
1c. Are there at least 3 simulations per simulation condition with statistical analysis?	☒	☐	Each system had at least 3 independent replicates. Values for equilibrium (Kd) and kinetic rate (Kon, Koff) constants are displayed as violin-plot distributions. The analyses and simulations conditions are stated in the methods.
1d. Is evidence provided in the text that the simulation results presented are independent of initial configuration?	☒	☐	As our simulation results, i.e., kinetic/equilibrium constants are derived from hundreds of spontaneous (un)binding events (uncorrelated in the simulation replicas) over the course of the microseconds long simulations, they can be safely assumed not to depend on the initial configuration.
**2. Connection to experiments**			
2a. Are calculations provided that can connect to experiments (*e.g.* loss or gain in function from mutagenesis, binding assays, NMR chemical shifts, J-couplings, SAXS curves, interaction distances or FRET distances, structure factors, diffusion coefficients, bulk modulus and other mechanical properties, *etc.*)?	☒	☐	We made calculations of pKa, as well as Kd, both of which can be (and are) directly compared to experimental quantities.
**3. Method choice**			
3a. Do simulations contain membranes, membrane proteins, intrinsically disordered proteins, glycans, nucleic acids, polymers, or cryptic ligand binding?	☒	☐	SLC26A11, a membrane-bound protein, was simulated in a pure POPC membrane.
3b. Is it described in the text whether the accuracy of the chosen model(s) is sufficient to address the question(s) under investigation (e.g. all-atom vs. coarse-grained models, fixed charge vs. polarizable force fields, implicit vs. explicit solvent or membrane, force field and water model, etc.)?	☒	☐	Simulations were performed using standard and verified force-fields (CHARMM36m). Non-polarizable force-fields were used. As for the divalent sulfate, extra care was taken to ensure experimentally relevant parameters were used (e.g., CUFIX; see methods).
3c. Is the timescale of the event(s) under investigation beyond the brute-force MD simulation timescale in this study that enhanced sampling methods are needed?	☐	☒	The events studied were ion binding, as opposed to gross conformational changes, thus we observed multiple spontaneous binding/unbinding events in the microseconds simulated.
If **YES**, are the parameters and convergence criteria for the enhanced sampling method clearly stated?	☐	☐	
If **NO,** is the evidence provided in the text?	☒	☐	The events studied were ion-binding, as opposed to gross conformational changes, thus we observed multiple spontaneous binding/unbinding events in the microseconds simulated.
**4. Code and reproducibility**			
4a. Is a table provided describing the system setup that includes simulation box dimensions, total number of atoms, total number of water molecules, salt concentration, lipid composition (number of molecules and type)?	☒	☐	Though a table is not included, all relevant information is provided in the methods (i.e., salt concentration, molecular species, force-fields, etc.). Additionally, the initial PDB files for each system are deposited in a public repository linked in the manuscript.
4b. Is it described in the text what simulation and analysis software and which versions are used?	☒	☐	See methods.
4c. Are other parameters for the system setup described in the text, such as protonation state, type of structural restraints if applied, nonbonded cutoff, thermostat and barostat, etc.?	☒	☐	See methods.
4d. Are initial coordinate and simulation input files and a coordinate file of the final output provided as supplementary files or in a public repository?	☒	☐	Initial/final system PDB files are deposited in a public repository linked in the manuscript.
4e. Is there custom code or custom force field parameters?	☒	☐	The code used to calculated Kd and kinetic rate constants is deposited in a public repository linked in the manuscript.
If **YES**, are they provided as supplementary files or in a public repository?	☒	☐	All relevant codes and PDB files are deposited in a public repository linked in the manuscript. Further details and trajectories will be provided upon request.

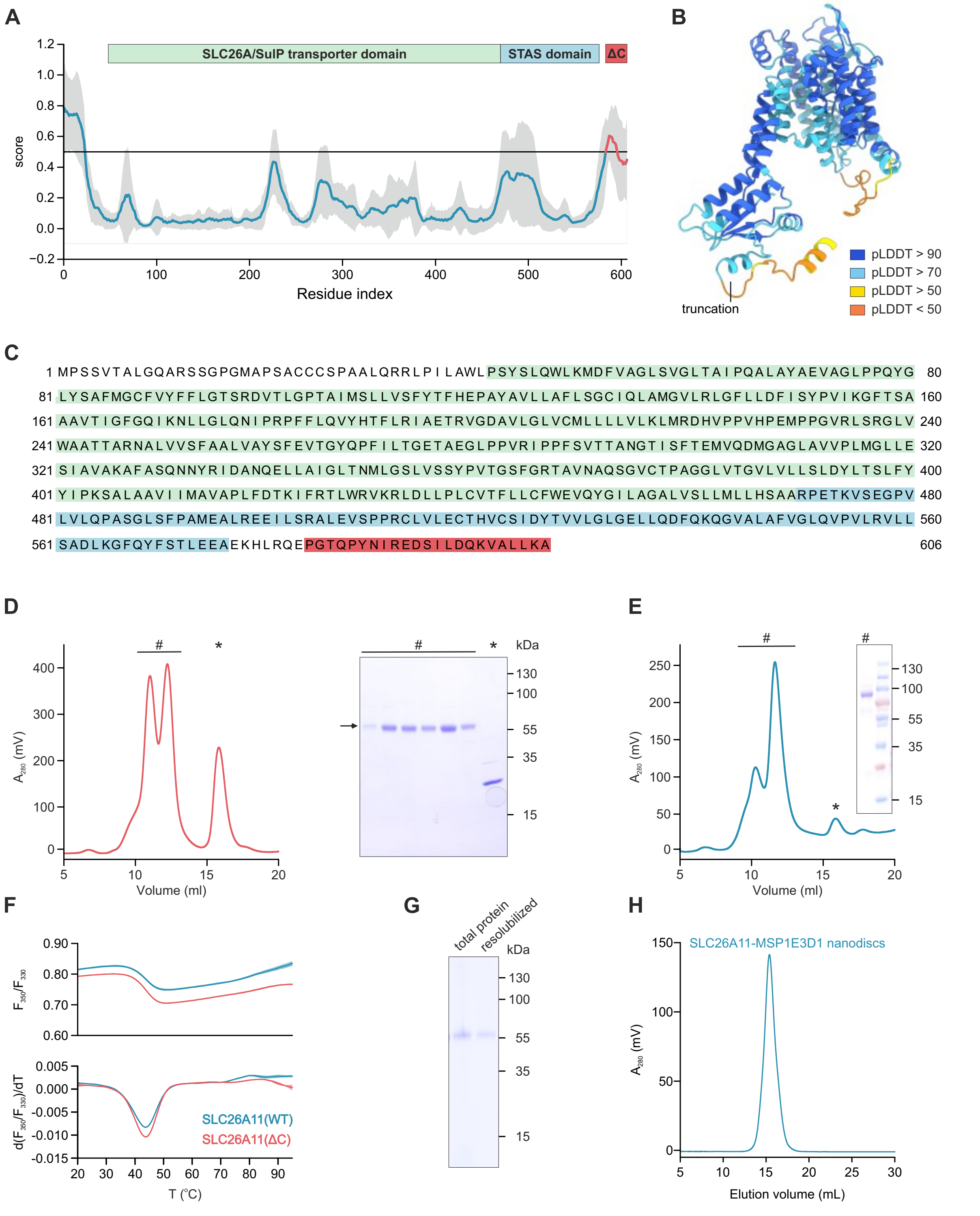

Supplementary Figure 1: C-terminal truncation of SLC26A11. (A) Combined intrinsically disordered region prediction for SLC26A11 based on Alphafold 2 pLDDT, IUPRED3, PONDR-VL-XT, PONDR-VL3, PONDR-VSL2 and Disopred3. **(B)** Alphafold model of SLC26A11 with color code showing the pLDDT and position of the C-terminal truncation as indicated. **(C)** SLC26A11 amino acid sequence showing the truncated C-terminus in red. **(D)** Size exclusion of detergent solubilized SLC26A11(ΔC) on a Superdex 200 increase 10/300 column and SDS-PAGE of elution fractions showing the monomeric and dimeric species of SLC26A11(ΔC) (#) and the cleaved C-terminal GFP (*). **(E)** Size exclusion of detergent-solubilized, IMAC-purified and HRV 3C protease-treated SLC26A11(WT) on a Superdex 200 increase 10/300 and SDS-PAGE of pooled fractions. Note the inefficiency of the 3C protease-treatment in cleaving the C-terminal GFP fusion protein as indicated by the small SEC peak at an elution volume of 16 ml (*) and the apparent migration position of the protein on SDS-PAGE. **(F)** Thermal unfolding of detergent solubilized SLC26A11(WT) and SLC26A11(ΔC). **(G)** SDS-PAGE of SoyPC-SLC26A11(ΔC) proteoliposomes with total protein and DDM resolubilized material recovered from supernatant after high speed ultracentrifugation indicating functional reconstitution of SLC26A11(ΔC). **(H)** Size exclusion of SLC26A11(ΔC)-MSP1E3D1 nanodiscs on a Superose 6 increase 10/300 column. The sample was injected twice to improve homogeneity. Shown is the re-injected size exclusion fraction of the first SEC run. The center of the peak was used for single-particle cryoEM.

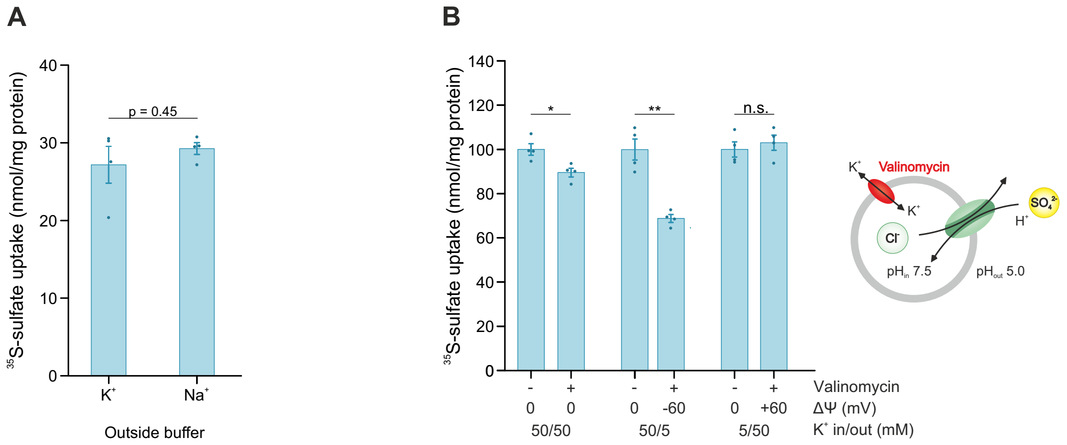

Supplementary Figure 2: Sulfate transport of SLC26A11$^{\Delta C}$ (A) Na^{+}-dependence of sulfate transport in proteoliposomes loaded with 50 mM K^{+} and diluted in buffer containing 50 mM K^{+} or Na^{+} as indicated. **(B)** Sulfate transport in presence of different membrane potentials generated by the addition of the K^{+}-ionophore valinomycin and K^{+}-gradients as indicated below the bars. A Two-tailed Student's t test was performed (** $p < 0.01$, * $p < 0.05$, p values are shown in the **Source Data file**). For all experiments, the individual datapoints as well as the mean ± SEM ($n \geq 3$) are shown. Assay buffers and precise number of replicates are detailed in **Supplementary Table 1**. Bar graphs represent sulfate accumulation levels reached after 16 min of transport.

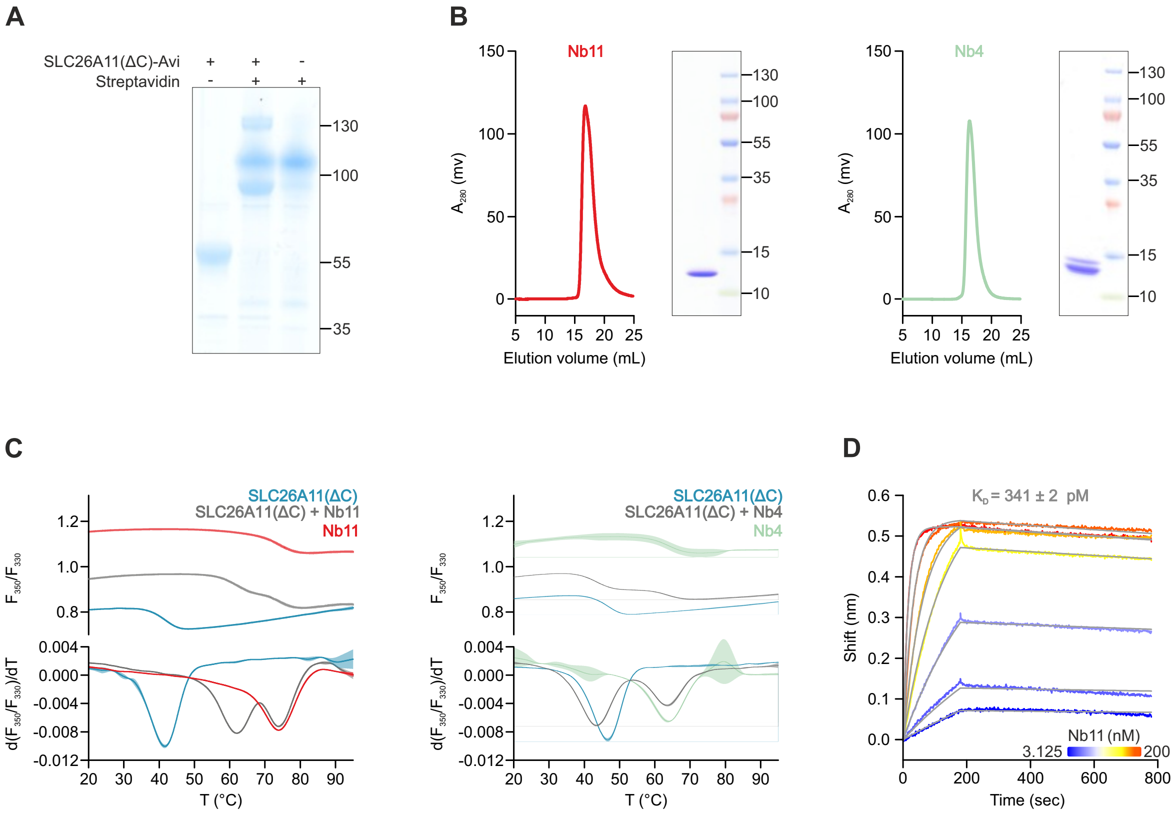

Supplementary Figure 3: Nanobody selection, purification and characterization. (A) Mobility shift in SDS-PAGE of enzymatically biotinylated SLC26A11(ΔC)-Avi in presence of Streptavidin. **(B)** Size exclusion chromatogram (Sepax SRT-10C SEC-300 column) and SDS-PAGE of purified Nb11 and Nb4. **(C)** Thermal unfolding of SLC26A11(ΔC) in presence of Nb11 or Nb4. **(D)** Bio-layer interferometry based affinity determination of Nb11 binding to immobilized SLC26A11(ΔC)-Avi.

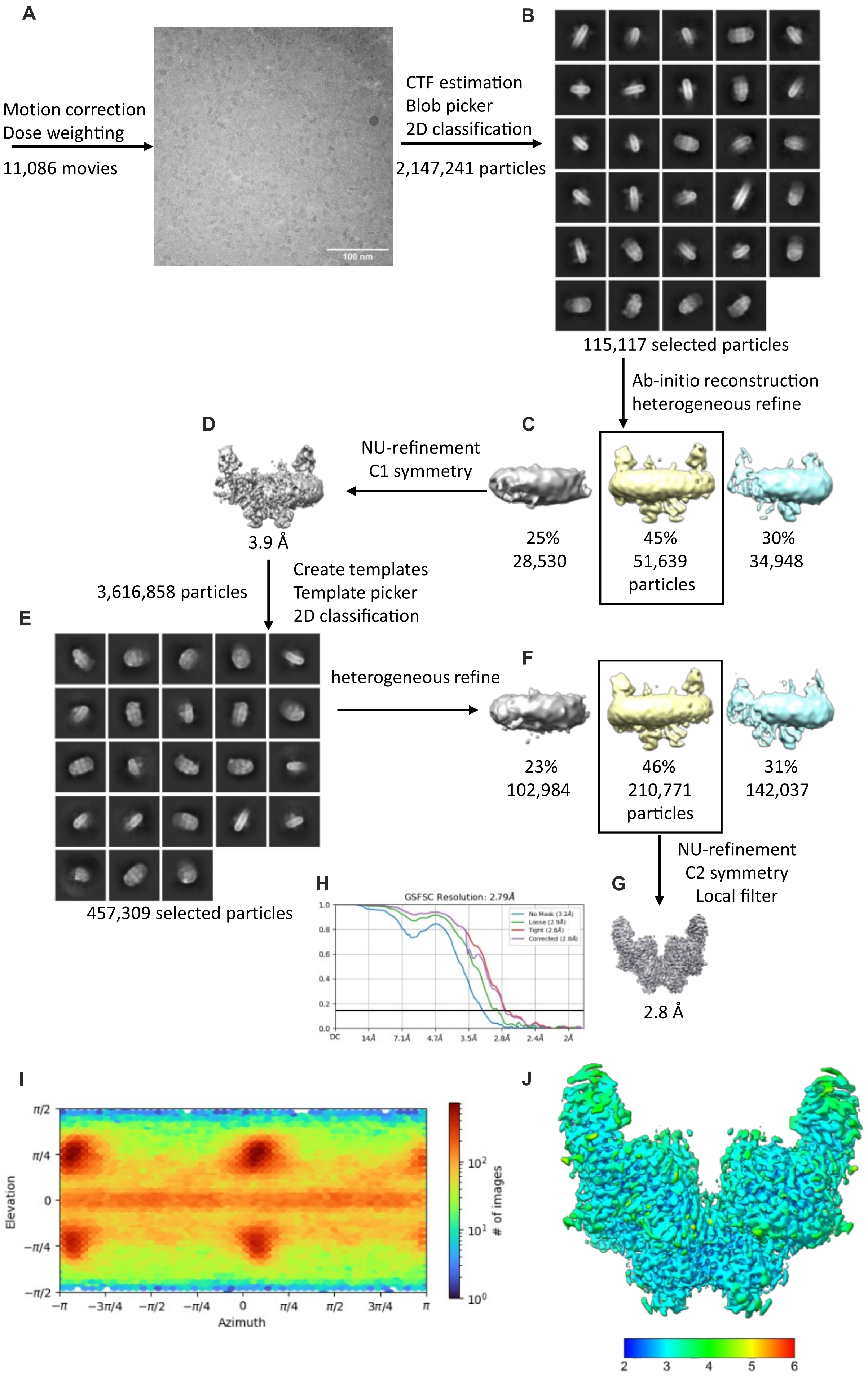
A
Motion correction
Dose weighting
11,086 movies
100 nm
CTF estimation
Blob picker
2D classification
2,147,241 particles
B
115,117 selected particles
Ab-initio reconstruction
heterogeneous refine
C
25%
28,530
45%
51,639
particles
30%
34,948
NU-refinement
C1 symmetry
D
3.9 Å
3,616,858 particles
Create templates
Template picker
2D classification
E
457,309 selected particles
heterogeneous refine
F
23%
102,984
46%
210,771
particles
31%
142,037
NU-refinement
C2 symmetry
Local filter
G
2.8 Å
H
GSFSC Resolution: 2.79Å
No Mask (3.2Å)
Loose (2.9Å)
Tight (2.8Å)
Corrected (2.8Å)
1.0
0.8
0.6
0.4
0.2
0.0
DC
14Å
7.1Å
4.7Å
3.5Å
2.8Å
2.4Å
2Å
I
Elevation
π/2
π/4
0
−π/4
−π/2
−π
−3π/4
−π/2
−π/4
0
π/4
π/2
3π/4
π
Azimuth
10^2
10^1
10^0
# of images
J
2
3
4
5
6

Supplementary Figure 4: Cryo EM workflow of SLC26A11 with Nb4. (A) Representative motion corrected micrograph of the dataset of the SLC26A11-Nb4 complex. Initially, 2,147,241 particles were picked with a blob picker and reduced to 115,117 particles by **(B)** 2D classification keeping particles in classes with the best-defined class averages. **(C)** Three initial volumes were determined in an ab-initio reconstruction. These volumes were used as starting references in a heterogeneous refinement. The classes were further refined by non-uniform refinement. **(D)** One of the classes provided a 3D-map, which was projected equally in space to create templates for template picking. Template picking identified 3,616,816 particles which were reduced to 457,309 particles by **(E)** 2D-classification and selection of the best 2D-classes. **(F)** The selected particles were analysed by another round of heterogeneous refinement. **(G)** One of the classes of the heterogeneous refinement with 210,771 particles was subjected to non-uniform refinement with imposed C2-symmetry. **(H)** GS-FSC plot of the final reconstruction reaching a nominal resolution of 2.8 Å resolution. **(I)** Angular distribution of the particles for the final refinements with C2 symmetry. **(J)** Final map colored according to local resolution.

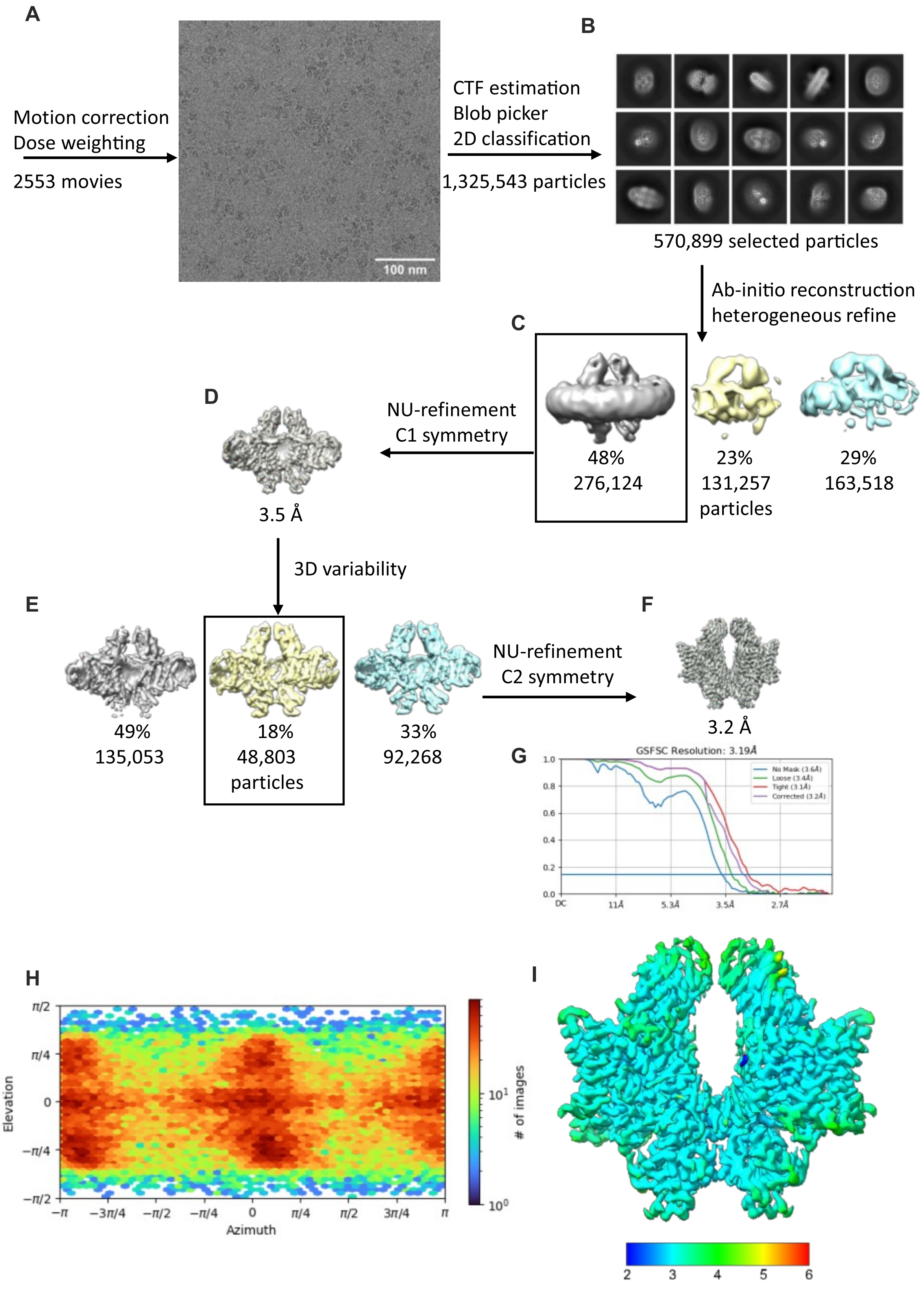

Supplementary Figure 5: Cryo EM workflow of SLC26A11 with Nb11. (A) Representative motion corrected micrograph of the dataset of the SLC26A11-Nb11 complex. The initial set of 1,325,543 particles were obtained with a blob picker and cleaned up by **(B)** 2D classification to 570,899 particles. **(C)** Three initial volumes were obtained by an ab-initio reconstruction. The initial volumes were used in a heterogeneous refinement as starting references. One of the resulting classes with 276,124 particles was further refined in a **(D)** non-uniform refinement with C1 symmetry and used as input for a **(E)** variability analysis with three principal components subdividing the data set into three clusters. **(F)** One of three clusters with 48,803 particles was then analysed in another non-uniform refinement with applied C2 symmetry. **(G)** GS-FSC plot of the final reconstruction reaching a nominal resolution of 3.2 Å resolution. **(H)** Angular distribution of the particles for the final refinements with C2 symmetry. **(I)** Final map colored according to local resolution.

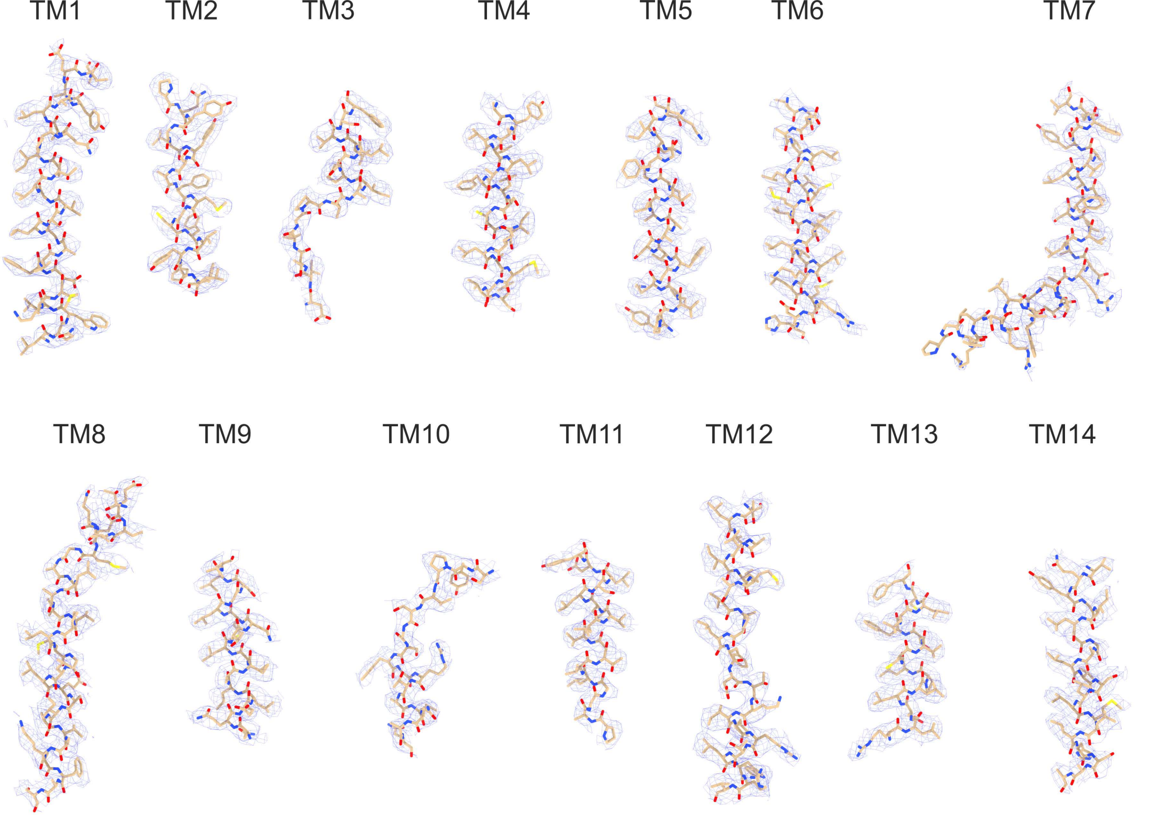

Supplementary Figure 6: Representative densities of SLC26A11-Nb11. Transmembrane segments shown as sticks with corresponding density (blue mesh).

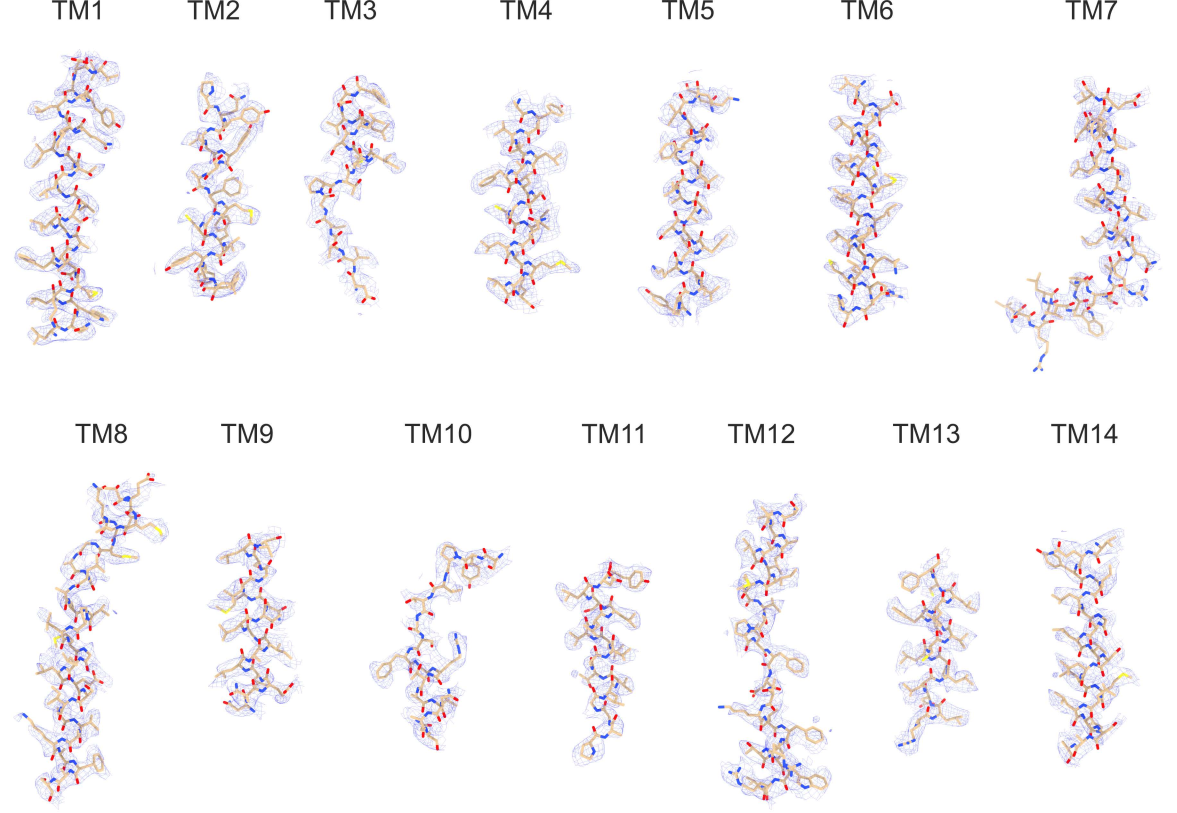

Supplementary Figure 7: Representative densities of SLC26A11-Nb4. Transmembrane segments shown as sticks with corresponding density (blue mesh).

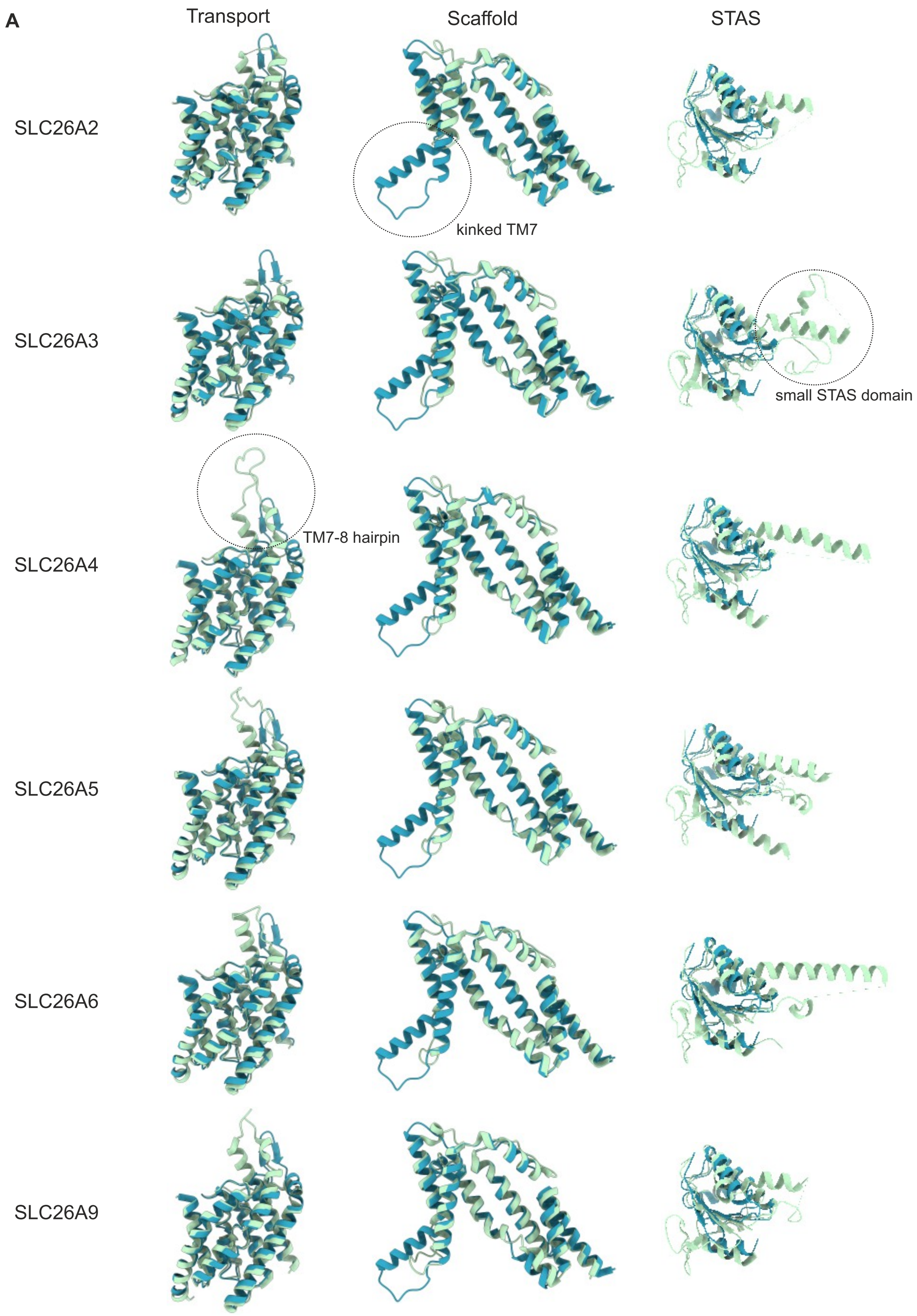
A
Transport
Scaffold
STAS
SLC26A2
kinked TM7
SLC26A3
small STAS domain
SLC26A4
TM7-8 hairpin
SLC26A5
SLC26A6
SLC26A9

B

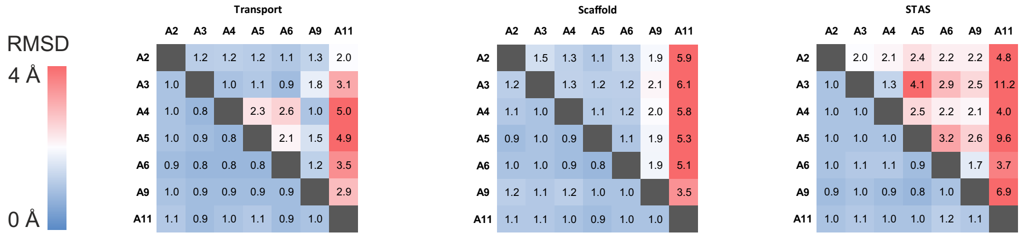

Supplementary Figure 8: The three unique structural motifs of SLC26A11. (A) Structural alignment based on the matchmaker command in UCSF ChimeraX of the individual domains of SLC26A11 (Transport, Scaffold and STAS, blue) with domains from all other human SLC26 isoforms (green) with available experimental high resolution structure. Unique structural features of SLC26A11 are highlighted by black circles. **(B)** RMSD from structural alignment of individual domains of human SLC26 isoforms in ChimeraX. Upper right shows results from all atom pairs and lower left from pruned atom pairs.

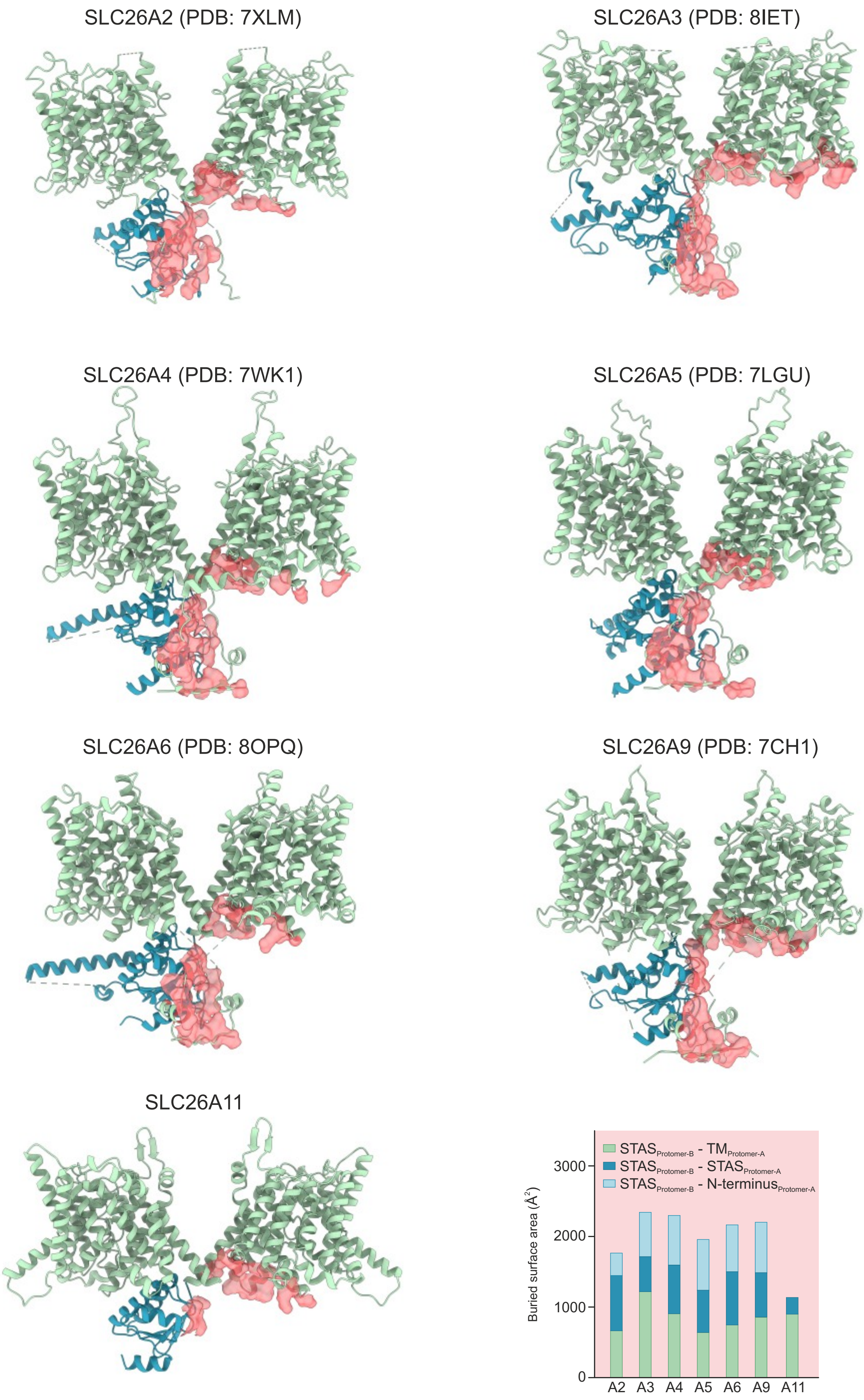

Supplementary Figure 9: STAS mediated dimer interface of human SLC26 transporters. Structures of human SLC26 isoforms with available experimental high-resolution structure with transmembrane domains in green and STAS domain in blue and the second STAS domain left out for clarity. Interfaces are indicated as red surface. Total interface size and contribution of transmembrane domain, STAS domain and N-terminus to the interface were quantified using the interfaces command in UCSF ChimeraX.

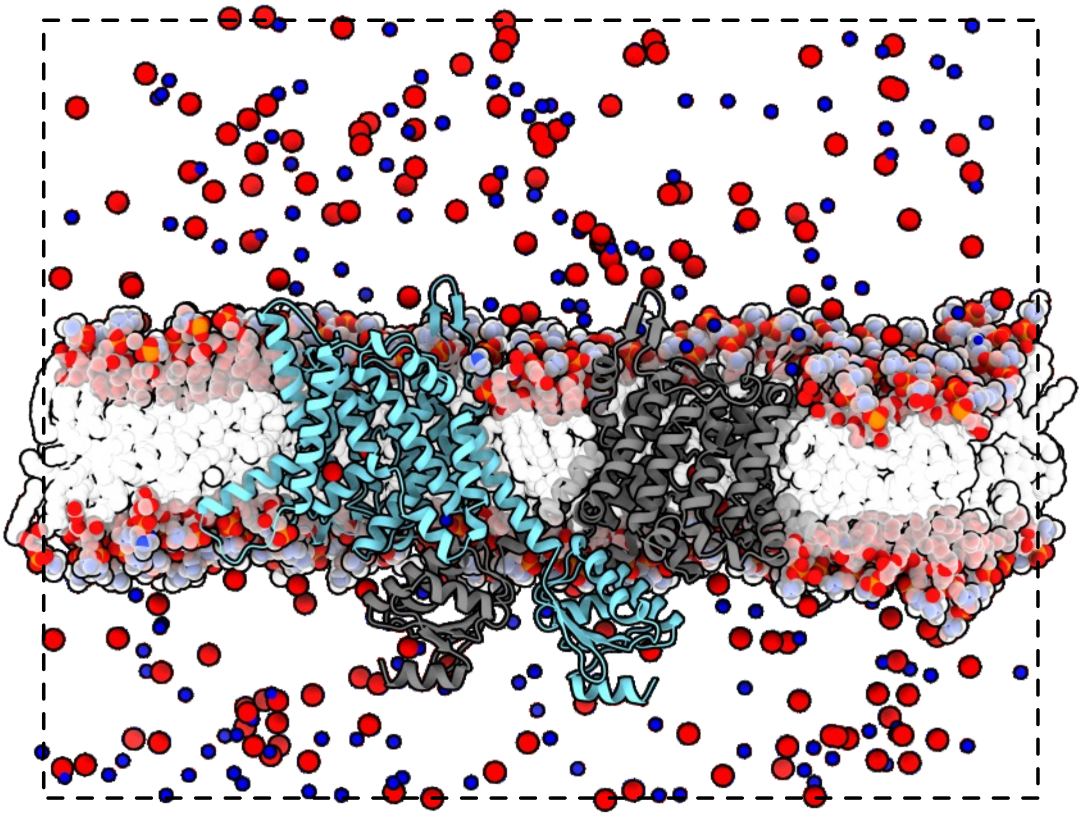

Supplementary Figure 10: SLC26A11 simulation system. Simulation system of SLC26A11 embedded in a POPC membrane, fully solvated (not shown) and ionized in ~200 mM Na^+ (blue)/Cl^- (red).

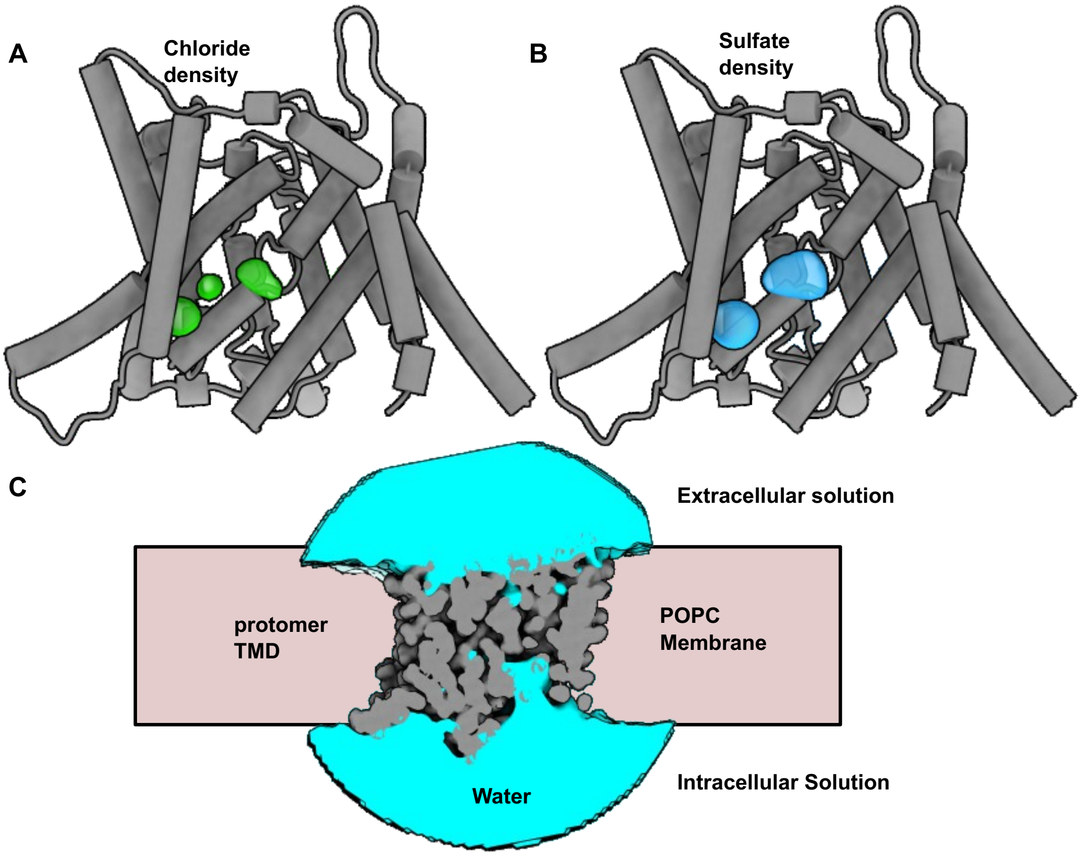

Supplementary Figure 11: Chloride and sulfate densities. Total averaged chloride **(A)** and sulfate **(B)** densities calculated from equilibrium MD simulations. Densities are shown at an iso-density threshold of 0.1 AMU/Å3. Densities are shown against the transmembrane domain of a single protomer. **(C)** Average water density around a TMD protomer.

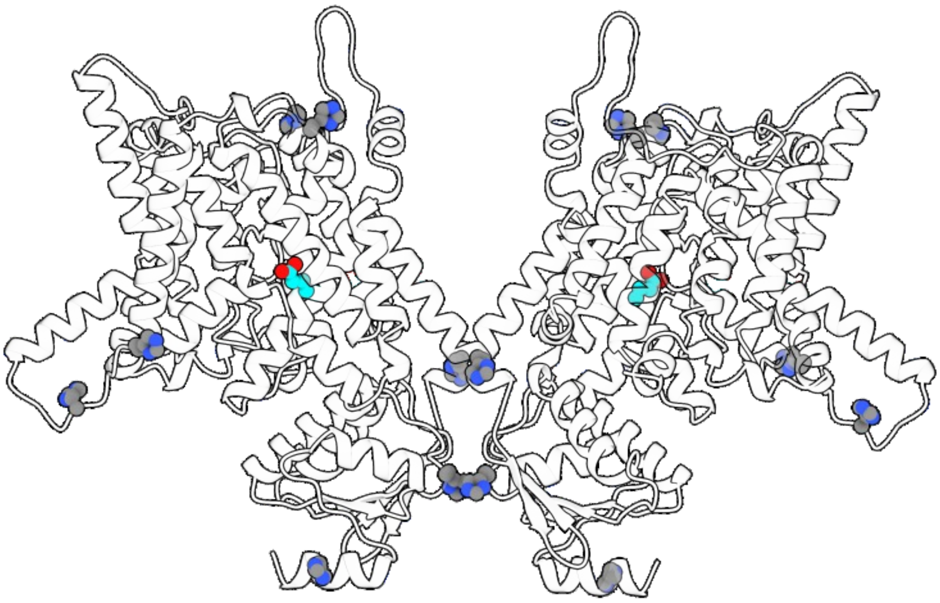

Supplementary Figure 12: Dimeric structure of SLC26A11 in cartoon representation. Location of histidine side chains exclusively on the intra and extra cellular sides of the protein is highlighted by grey sphere representation. The location of glutamate-320 is illustrated in cyan.

A

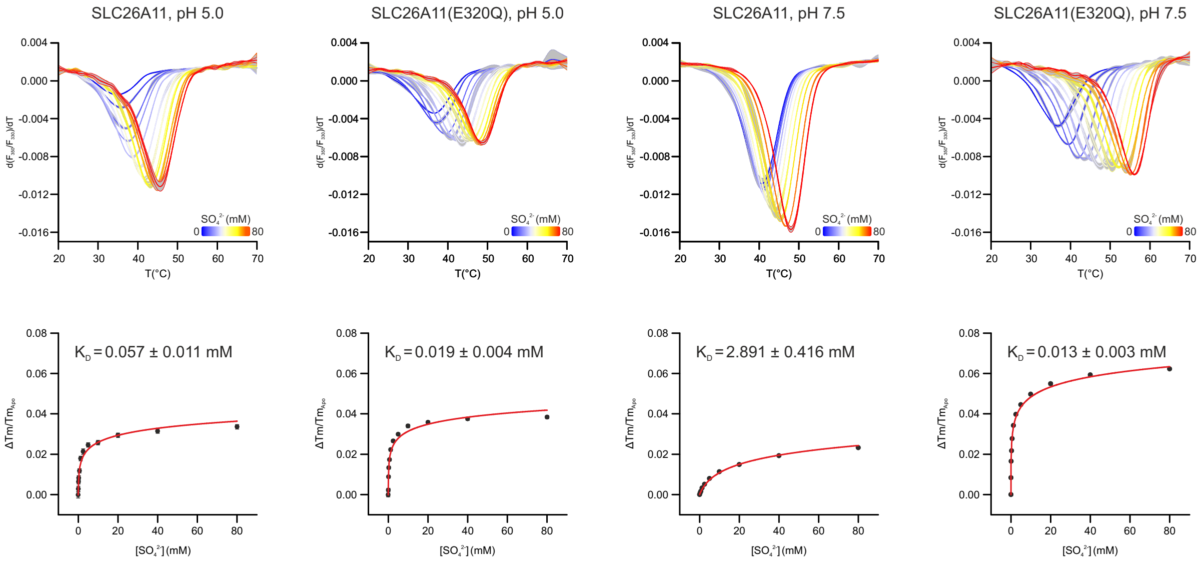

B

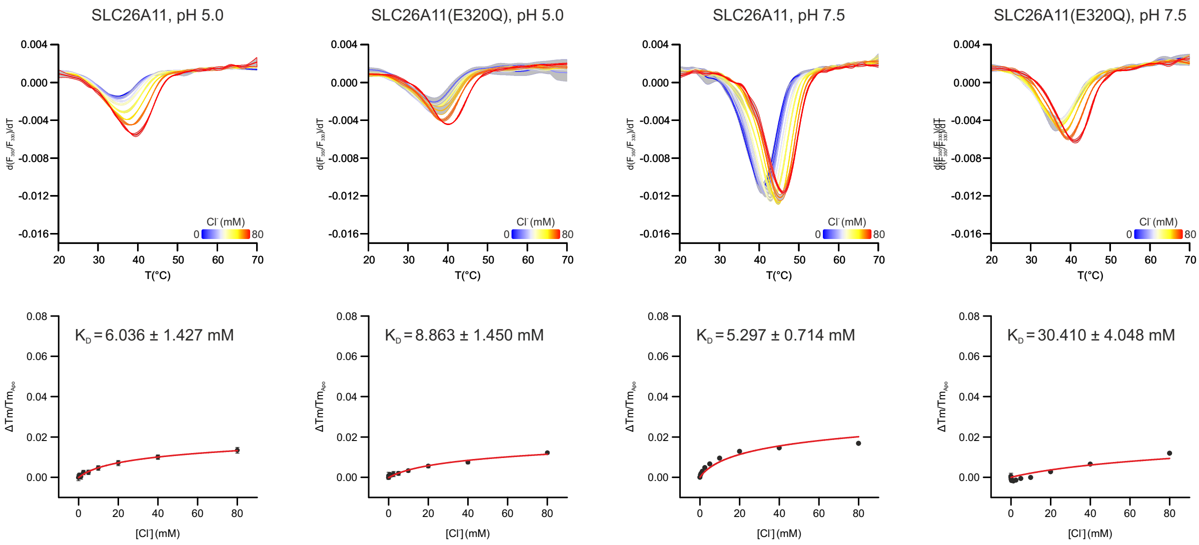

Supplementary Figure 13: pH dependent substrate binding. (A) Thermal unfolding of SLC26A11(ΔC) and SLC26A11(ΔC, E320Q) at pH 5.0 or pH 7.5 in presence of increasing concentrations of sulfate (blue = 0 mM sulfate, red = 80 mM sulfate) and derived dissociation constants (K_D). **(B)** Thermal unfolding of SLC26A11(ΔC) and SLC26A11(ΔC, E320Q) at pH 5.0 or pH 7.5 in presence of increasing concentrations of chloride (blue = 0 mM chloride, red = 80 mM chloride) and derived dissociation constants (K_D). Shown are mean and standard deviation (n = 3) of the first derivative of F_{350}/F_{330}. The melting temperature Tm is reported by the local minimum of $d(F_{350}/F_{330})/dT$.

A

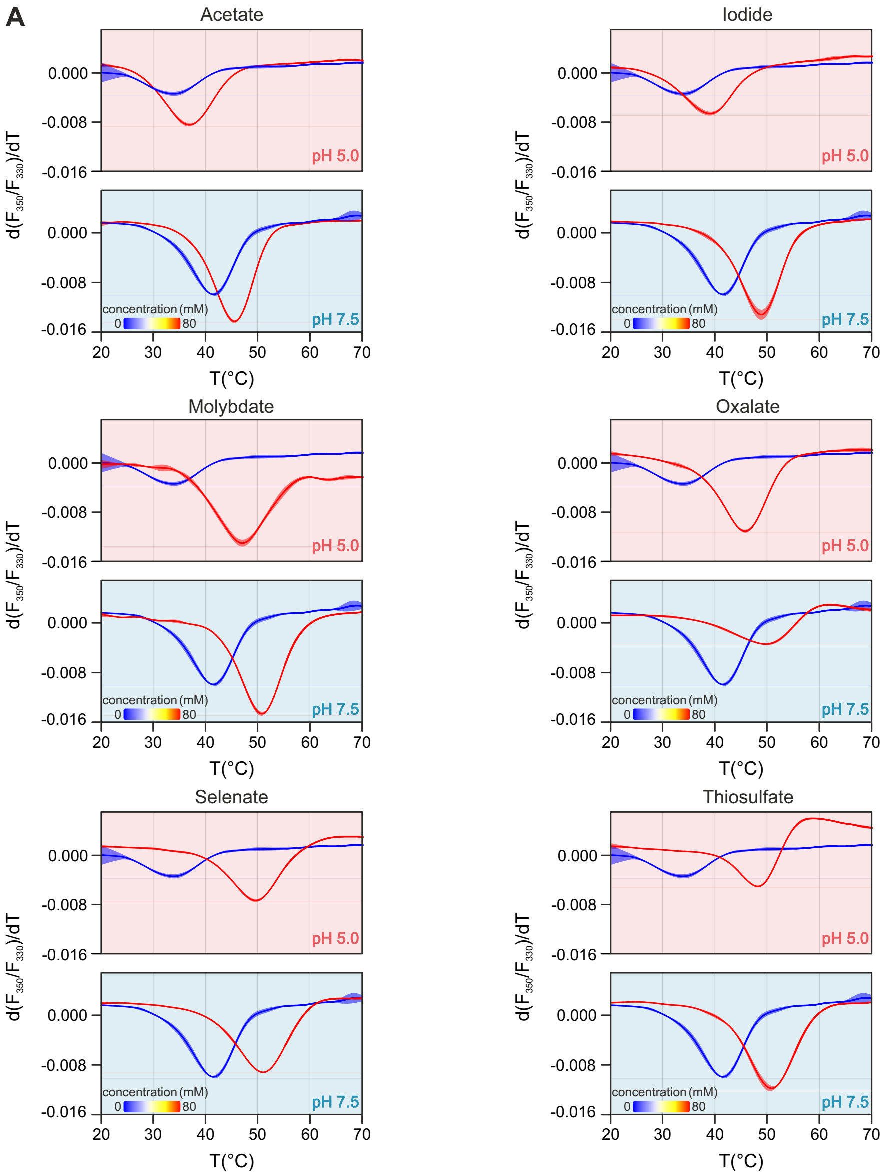

B

	pH 5.0 K_D (mM)	pH 7.5 K_D (mM)
Thiosulfate	0.009 ± 0.003	1.189 ± 0.365
Selenate	0.004 ± 0.001	1.121 ± 0.248
Oxalate	0.040 ± 0.011	2.110 ± 0.594
Molybdate	0.017 ± 0.005	1.241 ± 0.302
Iodide	2.950 ± 0.567	3.016 ± 0.640
Acetate	12.426 ± 1.393	15.522 ± 1.690

C

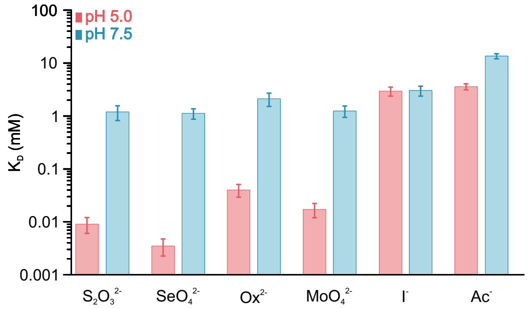

Supplementary Figure 14: pH dependent anion binding. (A) Thermal unfolding of SLC26A11(ΔC) in presence of 80 mM anion added as sodium salt at pH 5.0 or pH 7.5, respectively, with mean and S.D. (n=3). **(B)** pH dependent dissociation constants (K_D) of tested anions calculated from data shown in panel A. **(C)** Bar graph of data from panel B.

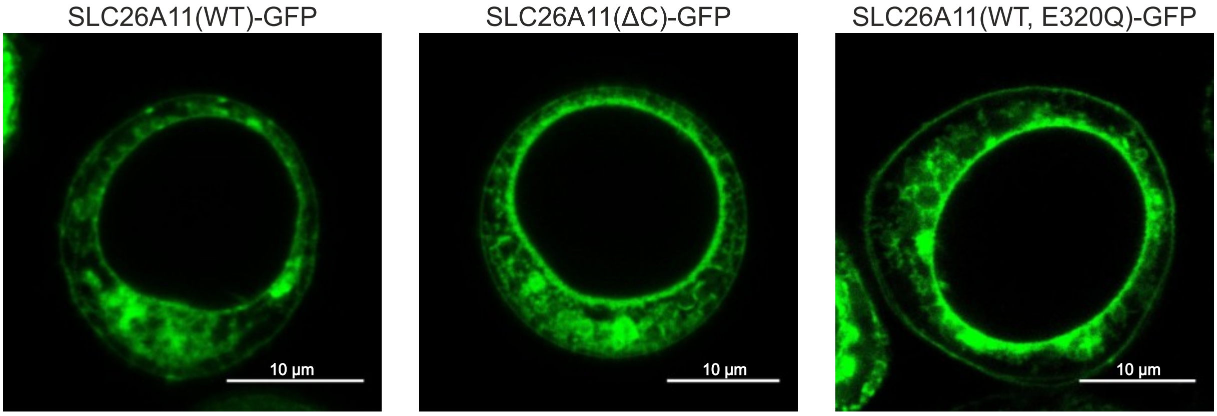

Supplementary Figure 15: Expression of SLC26A11-GFP constructs in Sf9 cells. Confocal microscopy of Sf9 cells transfected with either SLC26A11(WT), SLC26A11(ΔC) or SLC26A11(WT, E320Q) as indicated. All SLC26A11 variants show fractional localization to the plasma membrane with the majority of proteins localized to intracellular compartments.

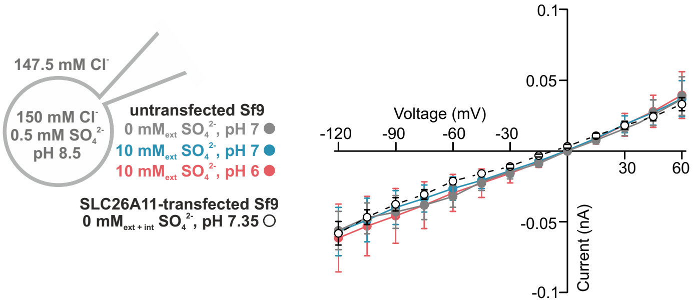

Supplementary Figure 16: Current-voltage relationships from untransfected *Sf*9 cells in three different bath solutions as indicated and transfected *Sf*9 cells in absence of internal sulfate. Shown are means and standard errors.

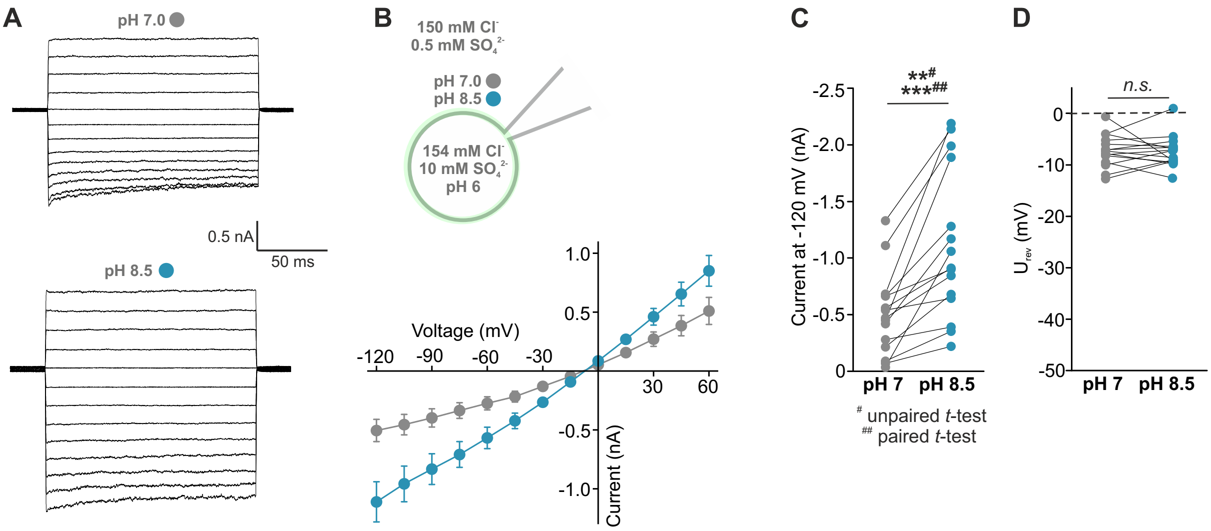

Supplementary Figure 17: Alkanisation stimulates channel currents during sulfate export. (**A**) Representative current recordings from *Sf*9 cells expressing SLC26A11(WT)-eGFP with buffer conditions as shown in the inset. (**B**) Current-voltage relationships from transfected *Sf*9 cells in two different bath solutions as indicated (grey: 140 mM Cl^-, pH 7; blue: 140 mM Cl^-, pH 8.5, n=15/15). Shown are means and standard errors. **(C)** Statistical analysis of current amplitudes at -120 mV for all experiments. An unpaired *t*-test show that means of current amplitudes are significantly different (p=0.004) and pairwise comparison show, that current stimulation by alkanisation was observed in all cases (p<0.001), whereas reversal potentials (**D**) remained constant (p=0.898).

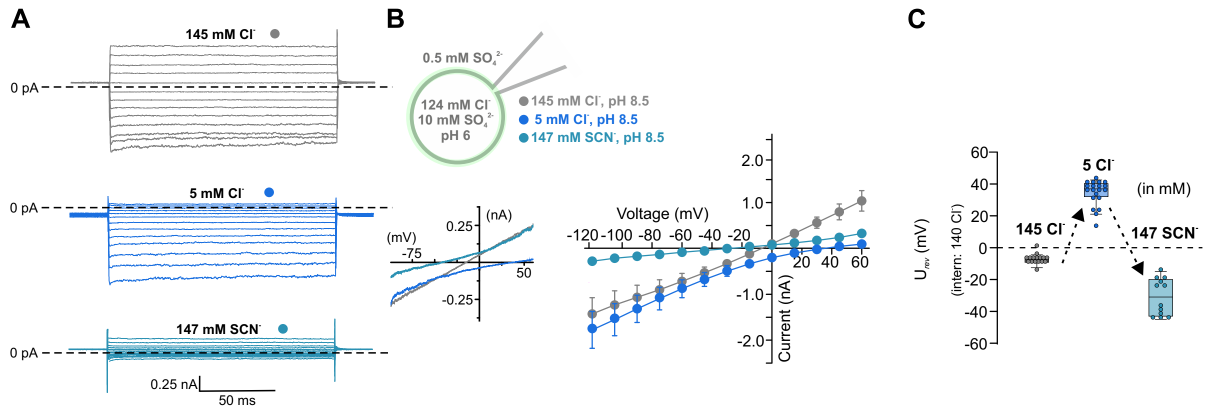

Supplementary Figure 18: SLC26A11 channel is anion-selective. (A) Representative current recordings from *Sf*9 cells expressing SLC26A11(WT)-eGFP with buffer conditions as shown in the inset. **(B)** Current-voltage relationships from transfected *Sf*9 cells in three different bath solutions as indicated (grey: 145 mM Cl^-, pH 8.5; blue: 5 mM Cl^-, pH 8.5; cyan: 147 mM SCN^-, pH 8.5_{ext}:6.0_{int}). Shown are means and standard errors. The inset in B shows current recordings from voltage ramps from a representative experiment. **(C)** Reversal potentials (U_{rev}) from experiments shown in panel A and B for WT (145 mM Cl^-, 5 mM Cl^-, 147 mM SCN^-: n=16/20/12).

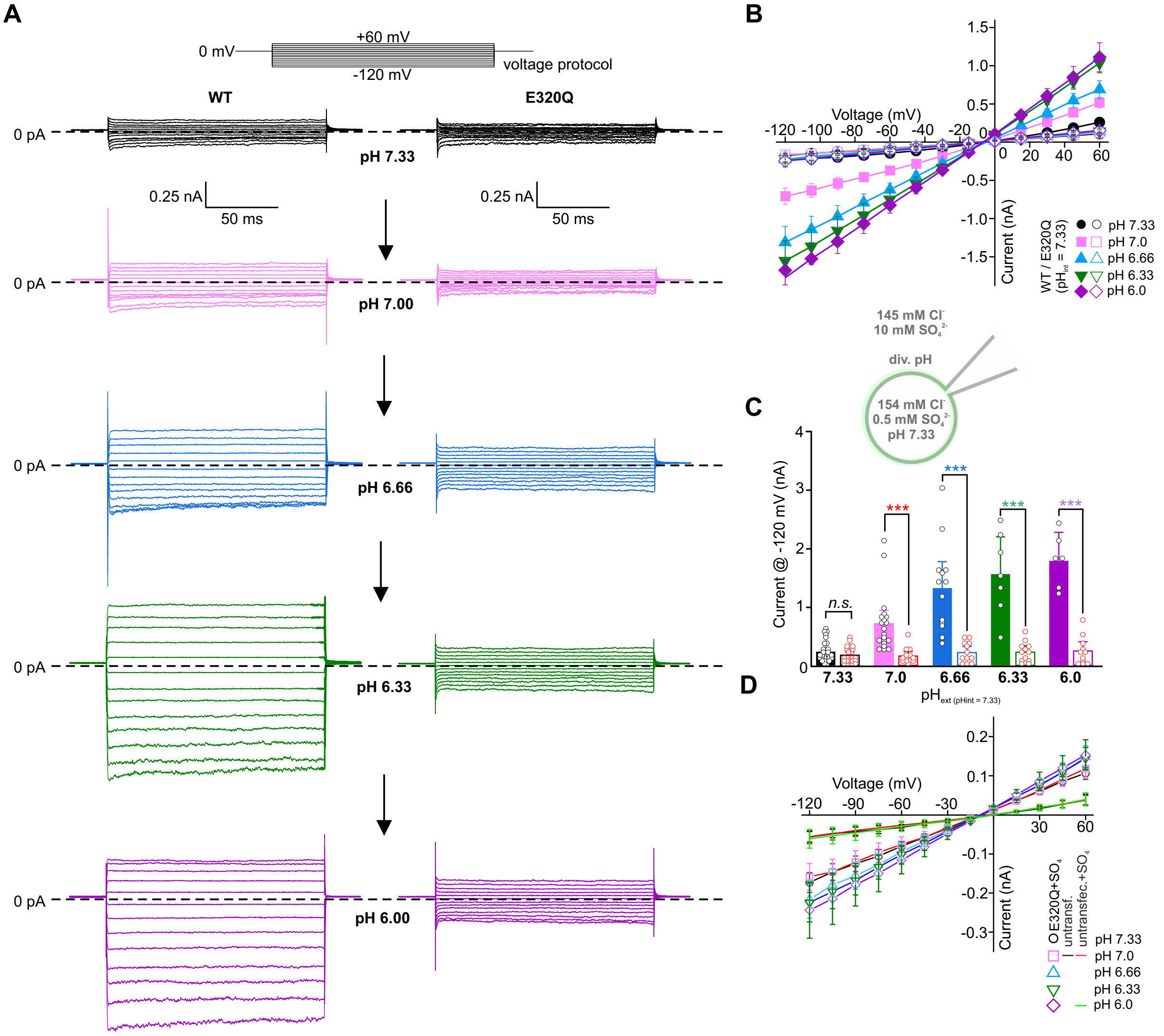

Supplementary Figure 19: Stimulation of SLC26A11 channel by external pH is abolished in E320Q mutant. (**A**) Representative current recordings from *Sf*9 cells expressing SLC26A11(WT)-eGFP (left) and SLC26A11(E320Q)-eGFP with buffer conditions as indicated in the inset. (**B**) Current-voltage relationships from transfected *Sf*9 cells (WT: closed symbols; E320Q: open symbols) in five different bath solutions as indicated (black: pH 7.33; magenta, pH 7.0; blue: pH 6.6, green: pH 6.33, violet: pH 6.0). **(C)** Statistical analyses of current amplitudes at -120 mV for all tested external pH values (*2-way* ANOVA with Holm-Sidak *posthoc* testing, $p<0.001$). (**D**) Current-voltage relationships of background currents from *Sf9* cells (lines, no symbols) and E320Q-mediated residual currents (lines, open symbols) for different ionic conditions. Shown are means and standard errors.

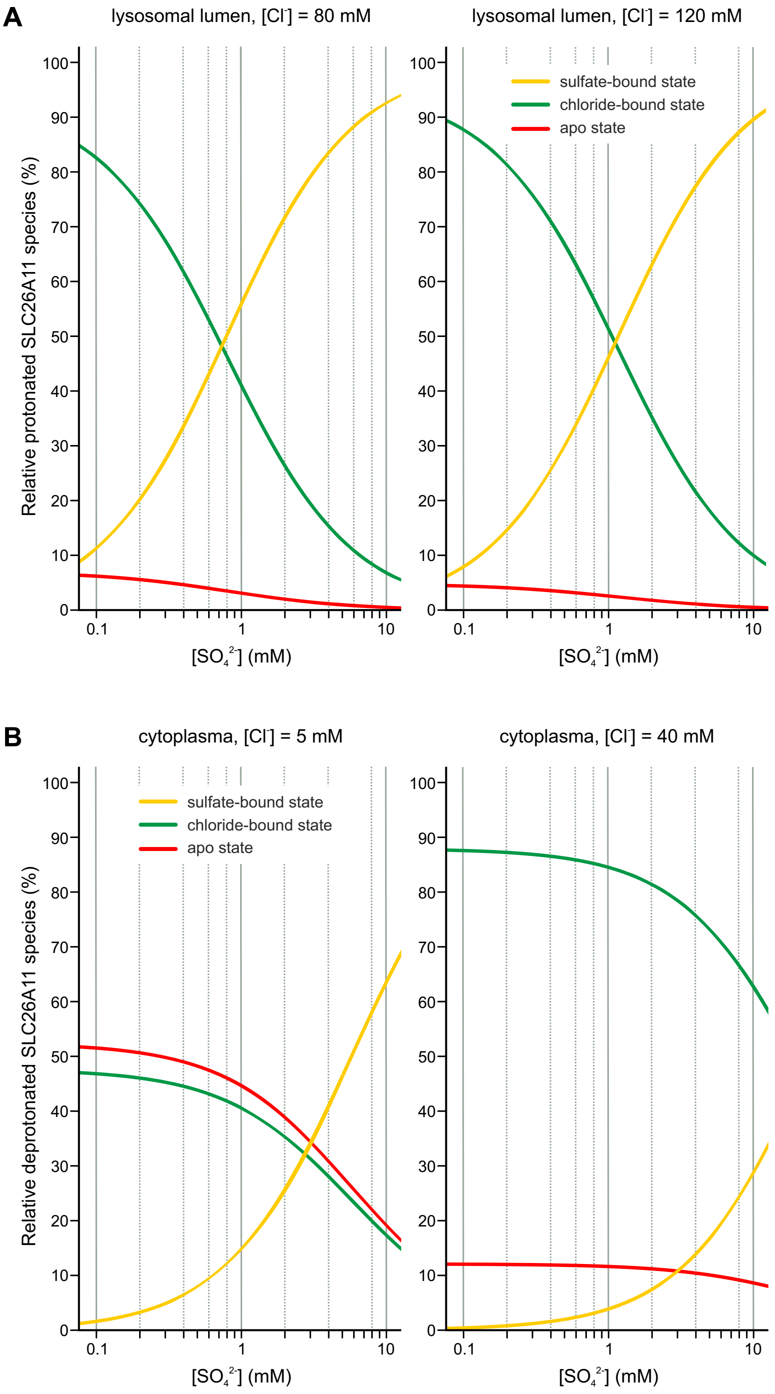

Supplementary Figure 20: Modeling of competitive binding of sulfate and chloride to SLC26A11. (**A**) Competitive binding of sulfate and chloride to the SLC26A11 substrate binding site exposed to the lumen of the lysosome and with Glu-320 in the protonated form. The fraction of each SLC26A11 species (sulfate-bound, chloride-bound, or apo) is depicted as a function of the lysosomal sulfate concentration. The model is based on the experimentally determined dissociation constants (sulfate: K_D 57 µM; chloride: K_D 6.0 mM) and luminal chloride concentrations of 80 mM (left panel) and 120 mM (right panel). (**B**) Competitive binding of sulfate and chloride to the SLC26A11 substrate binding site exposed to the cytoplasm and with Glu-320 in the deprotonated form. The fraction of each SLC26A11 species (sulfate-bound, chloride-bound, or apo) is depicted as a function of the lysosomal sulfate concentration. The model is based on the experimentally determined dissociation constants (sulfate: K_D 2.9 mM; chloride: K_D 5.3 mM) and cytoplasmic chloride concentrations of 5 mM (left panel) and 40 mM (right panel). Models were generated using the simulation applet from Pääkkonen *et al.*, 2022 (https://doi.org/10.1021/acsomega.2c00560).
