# Supplementary material for: SLC26A11 is an atypical solute carrier with dual transport-channel function mediating lysosomal sulfate transport
Source: Nat Commun. 2026 Jul 27;17:7407. doi: 10.1038/s41467-026-75749-4 (PMC13408680; doi:10.1038/s41467-026-75749-4)
Supplement: Supplementary file 3 — Transparent Peer Review file [file 41467_2026_75749_MOESM3_ESM.pdf]

nature portfolio

Peer Review File

SLC26A11 is an atypical solute carrier with dual transport-channel function mediating lysosomal sulfate transport

Corresponding Author: Dr Eric Geertsma

This file contains all reviewer reports in order by version, followed by all author rebuttals in order by version.

Version 0:

Reviewer comments:

Reviewer #1

(Remarks to the Author)
The manuscript titled "SLC26A11 is an atypical solute carrier with dual transport-channel function mediating lysosomal sulfate transport" reports functional characterizations and structural determinations of human SLC26A11. Functional analysis made it clear that SLC26A11 is an electroneutral exchanger that transports a proton and a sulfate ion in one direction and a chloride ion in the other direction. Structural studies led to the realization that E320 mediates proton transport and that its protonation enhances sulfate binding. This notion is tested and confirmed with molecular dynamics simulations and with extensive functional studies on both the wild type and the E320Q mutant. These results are highly significant in terms of advancing our understanding of sulfate transport in lysosomes and enhancing our comprehension of coupled ion transport in the SLC26 family of transporters. The experimental designs are thoughtful and the approaches are rigorous, and the conclusions are supported by the data.

I have a couple of thoughts but these are not requests for more experiments. First, I am curious that the authors did not show if E320Q breaks the electroneutrality in sulfate and chloride exchange. Second, could the authors be more clear why there is a voltage-dependency in one of the directions? Third, I am puzzled by the authors' claim of a sulfate- and proton-dependent (gated) conformation that conducts chloride ions. Would either sulfate or proton be bound to the transporter when it conducts chloride ions? Are there any sidedness in terms of where sulfate/proton activation could occur?

Reviewer #2

(Remarks to the Author)
The manuscript by Kuhn et al describes dual functional role for SLC26A11 lysosomal protein as a protein-driven sulfate/chloride exchanger as well as chloride channel. This is truly a multidisciplinary study that combines functional assays with binding assays, cryo-EM structure determination and computational MD simulations! The findings are novel and should be of interest to the journal audience.
I have several comments related to some of the data interpretation:

1. Page 4, lines 106-109: In relation to Fig. 1D, the manuscript states that the data “indicates that chloride is not an allosteric modulator of SLC26A11, but rather acts as a substrate competing with sulfate for the same binding site when present in the cis compartment, and functions as a counter-substrate when present in the trans compartment”. I am not sure I understand this argument. What is it in the data shown up to this point in the manuscript that suggests that chloride and sulfate are competing for the same binding site? Was there any competition assay performed? With subsequent data shown it becomes more reasonable to conclude about the existence of the common binding site, but I am not sure if at the place where this was stated, such conclusion was valid.

2. Regarding the proposed mechanism, in Fig. 6, given that lysosome is enriched with chloride, why is chloride transport into the lysosome needed? As I understand, this movement would be against the chloride concentration gradient across the lysosomal membrane. Is the argument the following: chloride-channel activity of the protein temporarily reverses the chloride gradients so that than higher chloride concentration is established at the cytoplasmic side of the lysosome and then chloride gets transported back via SLC26A11 to facilitate sulfate efflux from the lysosome?

3. Much of the mechanistic discussion is centered around the binding site, and not much is said about the protein dynamics.

Given that the authors performed around 15 microseconds of MD simulations, some discussion of conformational sampling would be interesting. Is the protein remaining in the cryo-EM like state the whole time during MD? Are there any conformational changes upon binding/unbinding of sulfate/chloride? I realize that full transitions from the inward to occluded or outward open states may not be observed, but I wonder if there are any hints of what type of dynamic changes could occur during the transport or channel activity.

Reviewer #3

(Remarks to the Author)
Summary
The manuscript by Khul et al., report human SLC26A11 (KBAT) is a dual-function membrane protein: a lysosomal sulfate transporter that also exhibits a channel-like chloride conductance gated by proton:sulfate symport. A cryo-EM structure of SLC26A11 is presented, complemented by biochemical transport assays, electrophysiology, and molecular dynamics simulations. The authors propose that SLC26A11 acts as a sulfate/H+/chloride electroneutral exchanger, has channel-like activity and may represent the elusive lysosomal sulfate exporter.

Major Comments
1. Use of C-terminally truncated SLC26A11- the authors employed a C-terminally truncated construct of SLC26A11 for both structural and functional analyses. Prior studies on SLC26A9—a closely related family member—show that its C-terminus acts as an autoinhibitory gate regulating transport activity (see: Zhang et al., Cell Discovery, 2020). It would be valuable for the authors to:
- Provide a sequence alignment comparing the C-terminal regions of SLC26A11 with SLC26A9 and other SLC26 family members.
- Discuss whether a similar autoinhibitory mechanism might exist in SLC26A11 and test experimentally or at least discuss.
- Better explain why the full-length construct could not be used. It is mentioned that the truncated version had higher expression and was more stable, but no data provided. The full-length protein appears to be more stable as a monomer, can the authors discuss this.

2. Cryo-EM data processing and resolution improvement
- The authors report two cryo-EM reconstructions at 2.8 Å and 3.2 Å resolution. Since SLC26A11 is a homodimer, it would be informative to know whether C2 symmetry expansion and focused refinement on a single protomer was attempted to further improve local resolution.
- Have the authors considered particle polishing in RELION to enhance particle alignment and map quality? Including such details would strengthen the methodological rigor.

3. Protein purification and sample characterization
- In Supplementary Fig. 1D and 1E, it is unclear whether the SEC traces correspond to the protein after detergent solubilization or nanodisc reconstitution. The authors should clearly specify this and provide the final SEC profile of the nanodisc-reconstituted sample used for cryo-EM.
- Please clarify whether only the dimer peak was concentrated and used for cryo-EM grid preparation.

4. Substrate binding and buffer conditions. The authors purified the protein in NaCl-containing buffer. Was purification in a sulfate-containing buffer tested? This might help visualize bound substrate density in the cryo-EM map and support their proposed substrate-binding site.

5. The manuscript refers to “cis” and “trans” sides in transport assays without defining them. These terms should be clearly explained (e.g., which side corresponds to the lumenal vs. cytosolic compartment).

6. In Fig. 1B, the background level of uptake (empty liposomes) should be shown and here (and elsewhere) a t-test is not appropriate as you are comparing multiple data sets to one (symmetrical pH 7.5). An Anova with an appropriate post-hoc would be more appropriate.

7. Were the electrophysiology experiments in SF9 performed on the full length or truncated SLC26A11? The manuscript suggests these experiments were performed on WT. Did the authors compare the function of full-length to the truncated form that was used for the proteoliposome experiments?

8. In Fig. 6 there is a statement that “Representations of SLC26A11 with the substrate binding site open to the lysosomal lumen or with a channel-like configuration are toy models”. While I appreciate the authors are pointing out these are not real structures, this could be consuming to the reader and make-believe structures with a continuous pore (such as in step *) should not be represented in structure form – if a structure that doesn’t exist. Cartoon representation should be used in the absence of structural information. Can the open-to-the-lumen side not be modelled from structures of other SLC26 members in a similar state?

9. The comparison to the dual function EAATs where the chloride conductance is uncoupled should be softened. There have been many studies over a long time that have demonstrated this process is uncoupled and the nature of the conductance studied in detail. This situation is not the same as chloride is coupled to the transport/exchange process and

the experiments to determine whether these processes are uncoupled have not been performed. It is also not clear that the authors have demonstrated “the importance of chloride in accelerating the transport mode” (line 382) which appears to be referring to the channel activity as chloride is required for the transport process.

10. In general, n values are low (Supp Table 1) and the valinomycin experiment in Supp Fig 2b should show initial rates of transport rather than uptake at 16 min. Membrane potentials generated by the addition of valinomycin indicated on the graph.

11. The statement that “For electrogenic transport, a more pronounced reduction at -60 mV and a concomitant increase in sulfate accumulation at +60 mV would be expected” is not backed up by the data. There is a significant reduction -60mV and without initial rates of transport, it is difficult to interpret what is going on here.

Minor comments
12. Structural presentation and figure quality - the density maps shown in Supplementary Figs. 6 and 7 appear low in quality and difficult to interpret. The authors should improve the map visualization.
13. On page 10, line 244, the text should reference Supplementary Fig. 13 for clarity.
14. In Fig. 1E, explicitly state that the assay measures [^{35}S]-sulfate uptake.
15. In Fig. 1F, include clear legend labels or color annotations to indicate what each curve represents.

I co-reviewed this manuscript with one of the reviewers who provided the listed reports. This is part of the Nature Communications initiative to facilitate training in peer review and to provide appropriate recognition for Early Career Researchers who co-review manuscripts.

Version 1:

Reviewer comments:

Reviewer #1

(Remarks to the Author)
The authors have address my comments.

Reviewer #2

(Remarks to the Author)
The revised version of the manuscript satisfactorily addresses my comments.

Reviewer #3

(Remarks to the Author)
The authors have addressed most of my comments and clarified several points that have improved the manuscript significantly.

I am still not overly convinced of the electroneutral transport mechanism interpretation. As the data is not clear cut (as stated by the authors), I suggest initial rates of uptake for this protein (SLC26A11), in this system, could be informative and back up the authors interpretation.

Open Access This Peer Review File is licensed under a Creative Commons Attribution 4.0 International License, which permits use, sharing, adaptation, distribution and reproduction in any medium or format, as long as you give appropriate credit to the original author(s) and the source, provide a link to the Creative Commons license, and indicate if changes were made.
In cases where reviewers are anonymous, credit should be given to 'Anonymous Referee' and the source.
The images or other third party material in this Peer Review File are included in the article’s Creative Commons license, unless indicated otherwise in a credit line to the material. If material is not included in the article’s Creative Commons license and your intended use is not permitted by statutory regulation or exceeds the permitted use, you will need to obtain permission directly from the copyright holder.
To view a copy of this license, visit https://creativecommons.org/licenses/by/4.0/

Reviewer comment

Author feedback

Original text

Revised text

REVIEWER COMMENTS

Reviewer #1 (Remarks to the Author):

The manuscript titled "SLC26A11 is an atypical solute carrier with dual transport-channel function mediating lysosomal sulfate transport" reports functional characterizations and structural determinations of human SLC26A11. Functional analysis made it clear that SLC26A11 is an electroneutral exchanger that transports a proton and a sulfate ion in one direction and a chloride ion in the other direction. Structural studies led to the realization that E320 mediates proton transport and that its protonation enhances sulfate binding. This notion is tested and confirmed with molecular dynamics simulations and with extensive functional studies on both the wild type and the E320Q mutant. These results are highly significant in terms of advancing our understanding of sulfate transport in lysosomes and enhancing our comprehension of coupled ion transport in the SLC26 family of transporters. The experimental designs are thoughtful and the approaches are rigorous, and the conclusions are supported by the data.

We thank Reviewer #1 for this positive feedback.

I have a couple of thoughts but these are not requests for more experiments.

First, I am curious that the authors did not show if E320Q breaks the electroneutrality in sulfate and chloride exchange.

The E320Q mutant shows a very low sulfate transport efficiency (Fig. 4F). We have explained this phenotype with the high and pH-independent sulfate binding affinity that is likely to decrease unbinding of sulfate in the lumen of the proteolipomes. We expect this sulfate-bound E320Q to oscillate between the inward-open and outward-open conformations, but not to contribute to net sulfate uptake. The reviewer is correct that in addition, a loss of electroneutrality and the concomitant build-up of a membrane potential, could contribute to the very low sulfate transport efficiency.

We have now modified our statement (line 255-260) to reflect that the loss of electroneutrality offers an alternative and complementary explanation for the poor transport efficiency of E320Q.

*“Finally, we did not observe any (cis) proton- and (trans) chloride-driven sulfate accumulation in SLC26A11$^{\Delta C}$(E320Q) proteoliposomes **[Fig. 4F]**. This is consistent with the mutant's very high, pH-independent sulfate binding affinity, which likely results in a high prevalence of the sulfate-bound state in both compartments. Furthermore, any alteration in the electroneutrality of transport resulting from the E320Q mutation would generate an inhibitory membrane potential, thereby reducing sulfate uptake in the liposomal lumen further.”*

Second, could the authors be more clear why there is a voltage-dependency in one of the directions?

We assume this point refers to Suppl. Fig. 2B. Here, we observe a decrease in sulfate uptake of ~30% ($p<0.01$) in the presence of an inside-negative membrane potential ($\Delta\Psi$ = -60 mV) while an inward-positive potential ($\Delta\Psi$ = +60 mV) elicits no significant effect. This data strongly suggest an electroneutral transport mode of SLC26A11, as electrogenic transport modes are expected to lead to effects that are: *a)* stronger, and *b)* of opposite magnitudes for positive and negative potentials (line 123-130).

We have refrained from explaining the observation of voltage-dependency in one of the directions as we can only speculate why we exclusively observe an effect with a negative membrane potential. For example, this phenotype may result from a bias in the direction to which the protein reconstitutes in the membrane, e.g., more inside-out than right-side-out, in combination with a voltage-sensitive inhibition that only occurs for protein reconstituted in one of the directions.

To reflect that we do not fully understand the voltage-dependence in only one direction, we have updated the text (line 127-135).

We observed only modest changes when generating a membrane potential of 0 and +60 mV, but at -60 mV sulfate accumulation was reduced by approximately 30%. For electrogenic transport, a more pronounced reduction at -60 mV and a concomitant increase in sulfate accumulation at +60 mV would be expected, as shown for the proton:fumarate symporter SLC26Dg [13]. Though the reduced accumulation levels at -60 mV resist simple interpretation, these results together support an electroneutral transport mode for SLC26A11. In the most parsimonious model, SLC26A11 is an exchanger that couples the symport of one proton and one sulfate ion (net charge of -1) to the antiport of one chloride ion (net charge of -1).

Please find additional comments regarding this point in our feedback to Question 11 from Reviewer 3.

Third, I am puzzled by the authors' claim of a sulfate- and proton-dependent (gated) conformation that conducts chloride ions.
Would either sulfate or proton be bound to the transporter when it conducts chloride ions?
Are there any sidedness in terms of where sulfate/proton activation could occur?

We assume a sulfate ion to be bound during chloride conductance: we do not observe chloride currents in the absence of sulfate (Fig. 5B), indicating that the transporter assumes the chloride-conducting conformation in a sulfate-bound state. There is no sidedness in terms of where sulfate activation occurs, sulfate can activate from the outside (Fig. 5B) as well as from the inside (Supplementary Figure 17).

We further assume that proton-binding is one of the required steps preceding formation of the chloride-conducting state, but that the chloride conductance emerges following release of the proton from the proton+sulfate-bound state. This is based on the conductance of E320Q, that is a fraction of that observed for the wild type protein at an external pH of 6.0 (Fig. 5D-F). This suggests that chloride conductance requires Glu-320 to alternate between a protonated and a deprotonated state, something that the E320Q mutant, which permanently mimics the protonated state, cannot do.

The need for protonation and deprotonation of Glu-320 is further reflected in our observation that chloride conductance is enhanced if the pH-gradient is enhanced in the same direction of the sulfate-gradient. Whereas external acidification activates SLC26A11 anion channels with an inwardly directed sulfate gradient (Fig. 5A-C), chloride currents were larger at an external pH of 8.5 than at pH 7.0 in cells with an outwardly directly sulfate gradient (Supplementary Figure 17). Thus, concerning protonation and deprotonation one can speak about a sidedness that is relative to the sulfate gradient.

Taken together, our data suggest that to assume a chloride conductance, SLC26A11 requires Glu-320 to alternate between a protonated and a deprotonated state. Since the chloride conductance requires sulfate (Fig. 5A-C) and assuming that the conducting state emerges from a conformational state part of the conventional transport cycle, we conclude that the chloride conductance must occur between the sulfate-bound protonated state (state 3 in Fig. 6) and the sulfate-bound deprotonated state (state 4 in Fig. 6).

Reviewer #2 (Remarks to the Author):

The manuscript by Kuhn et al describes dual functional role for SLC26A11 lysosomal protein as a protein-driven sulfate/chloride exchanger as well as chloride channel. This is truly a multidisciplinary study that combines functional assays with binding assays, cryo-EM structure determination and computational MD simulations! The findings are novel and should be of interest to the journal audience.

We thank Reviewer #2 for this positive feedback.

I have several comments related to some of the data interpretation:

1. Page 4, lines 106-109: In relation to Fig. 1D, the manuscript states that the data “indicates that chloride is not an allosteric modulator of SLC26A11, but rather acts as a substrate competing with sulfate for the same binding site when present in the cis compartment, and functions as a counter-substrate when present in the trans compartment”. I am not sure I understand this argument. What is it in the data shown up to this point in the manuscript that suggests that chloride and sulfate are competing for the same binding site? Was there any competition assay performed? With subsequent data shown it becomes more reasonable to conclude about the existence of the common binding site, but I am not sure if at the place where this was stated, such conclusion was valid.

At this stage in the manuscript, we have entertained two scenarios: *i)* chloride is an allosteric modulator; and *ii)* chloride is a genuine substrate. Based on Fig. 1D, specifically, the last two columns, we conclude that chloride is not an allosteric modulator as only the presence of chloride in the opposite compartment (“trans”) with respect to the sulfate-containing compartment (“cis”) is stimulating transport. This brings us to the assumption that chloride is a genuine substrate. The reviewer correctly argues that this can be met by having either *a)* two separate binding sites for chloride and sulfate; or *b)* one binding site that both substrates compete for. The reviewer is correct that we cannot formally exclude the first scenario *at this stage of the manuscript*. But following the principle of parsimony, the one-site scenario remains the most simple and straightforward explanation for our observations (and one that is well accepted in the field). Indeed, our extensive all-atom MD simulations consistently revealed binding of both anions to the same site (Supplementary Fig. 10, 11). An additional indirect argument in favor of the one-site model are the available structures of SLC4, SLC23, and SLC26 proteins, which share the architecture of their membrane domain, that all depict only one substrate binding site (at the center of the transport domain on the side facing the scaffold domain).

To better reflect what we can formally conclude at this stage in the manuscript, we have now modified our statement (line 103-111).

Next, we systematically varied the chloride concentrations in both compartments to explain the stimulatory effect of trans chloride on sulfate transport ***[Fig. 1D]****. While the presence of chloride in the trans compartment led to strong sulfate accumulation, the presence of chloride in both compartments showed similar reduced uptake levels as observed in the absence of chloride. Sulfate uptake was further reduced in the presence of chloride in the cis compartment only. This indicates that chloride is not an allosteric modulator of SLC26A11. Instead, in the most parsimonious model, chloride could act as a substrate competing with sulfate for the same binding site when present in the cis compartment, and functions as a counter-substrate when present in the trans compartment.*

2. Regarding the proposed mechanism, in Fig. 6, given that lysosome is enriched with chloride, why is chloride transport into the lysosome needed? As I understand, this movement would be against the chloride concentration gradient across the lysosomal membrane. Is the argument the following: chloride-channel activity of the protein temporarily reverses the chloride gradients so that than higher chloride concentration is established at the cytoplasmic side of the lysosome and then chloride gets transported back via SLC26A11 to facilitate sulfate efflux from the lysosome?

The relevance of chloride counter-transport for the function of SLC26A11 is two-fold. First, SLC26A11 operates more efficiently with a counter-substrate than without (Fig. 1D, F) suggesting that the return of the unliganded binding site is rate-limiting. We thus argue that one role of chloride counter-transport lies in accelerating sulfate export.

This first role of chloride counter-transport is mentioned in the discussion (line 347-349).

Although deprotonated apo SLC26A11 appears to be translocation competent, its reorientation seems much slower than that of the chloride-bound state ***[Fig. 1A]****.*

Second, the counter-transport of one chloride ion *into* the lysosome compensates for the net transport of one elementary charge *out* of the lysosome in the first half of the transport cycle ($H^+:SO_4^{2-}$). This renders the SLC26A11 transport cycle electroneutral. Given that lysosomes assume a positive inside membrane potential, a hypothetical electrogenic $H^+:SO_4^{2-}$ symporter would experience a reduced driving force and thereby operate less efficient. In contrast, an electroneutral $H^+:SO_4^{2-}/Cl^-$ transporter operates independently from the membrane potential.

We have extended our discussion to highlight this aspect (line 350-354).

Finally, upon subsequent arrival in the lysosomal lumen, chloride is released and Glu-320 is rapidly protonated, which may lock the carrier until a new sulfate ion is bound. The counter-transport of chloride renders the transport cycle electroneutral. This prevents a reduction in driving force due to the lysosomal membrane potential that would be experienced by a hypothetical electrogenic $H^+:SO_4^{2-}$ symporter.

The reviewer is correct in stating that chloride import into the lysosome is against its chemical gradient. We suspect that the rapid protonation of Glu-320 in the lysosomal lumen prevents subsequent reorientation of the chloride-bound protonated binding site to the cytoplasmic side (line 340-343), thereby reducing the consequences of the thermodynamic penalty of chloride transport against its chemical gradient. We propose that, in addition, the SLC26A11 anion channel serves to increase the overall driving force of $H^+:SO_4^{2-}/Cl^-$ transport by reducing the lysosomal chloride concentration due to chloride efflux.

We have rephrased the relevant section in the discussion (line 382-391).

While the relevance of sulfate export from the lysosome is apparent, the necessity of the SLC26A11 chloride channel is not. Chloride is actively accumulated in the lysosome [weinert2010]. Thus, SLC26A11-mediated $H^+:SO_4^{2-}/Cl^-$ transport moves chloride against its chemical gradient. The rapid protonation of Glu-320 in the lysosome likely prevents the chloride-bound binding site from reorienting back to the cytoplasm, thereby reducing the consequences of the thermodynamic penalty for chloride accumulation. Uncoupled chloride conductance permits chloride diffusion out of the lysosome. We propose that this chloride efflux via the SLC26A11 anion channel serves to reduce the lysosomal chloride concentration, making $H^+:SO_4^{2-}/Cl^-$ transport more energetically favourable. Given the importance of chloride in accelerating the transport mode ***[Fig. 1A]****, this feedback would ensure high transport rates.*

3. Much of the mechanistic discussion is centered around the binding site, and not much is said about the protein dynamics. Given that the authors performed around 15 microseconds of MD simulations, some discussion of conformational sampling would be interesting.
Is the protein remaining in the cryo-EM like state the whole time during MD?
Are there any conformational changes upon binding/unbinding of sulfate/chloride? I realize that full transitions from the inward to occluded or outward open states may not be observed, but I wonder if there are any hints of what type of dynamic changes could occur during the transport or channel activity.

Our unbiased MD simulation, and the simulation timescales in particular, were primarily designed to quantitatively investigate ion-binding kinetics and thermodynamics of these interactions. To address your comment, we performed C-alpha root mean squared deviation (RMSD) calculations over the roughly 15 µs of total simulation time. We focused our analysis on the following domains: transport domain (TD), scaffold domain (SD), transmembrane domain (TMD), transmembrane-helix 3 (TM3), transmembrane-helix 10 (TM10), and the STAS domain. RMSD calculations were performed such that each structural element is aligned on itself before the calculation—alignments were performed at the protomeric and dimeric levels respectively. In all cases the cryo-EM structure was used as the reference for alignment and RMSD calculation.

Overall, our analyses of individual protomers and dimers suggest that, in the total simulation time of ~15 µs, the protein remains stably in a state close to the cryo-EM structure. We do not observe any

anion-dependent changes in conformation. Please find a more detailed breakdown of our analyses below.

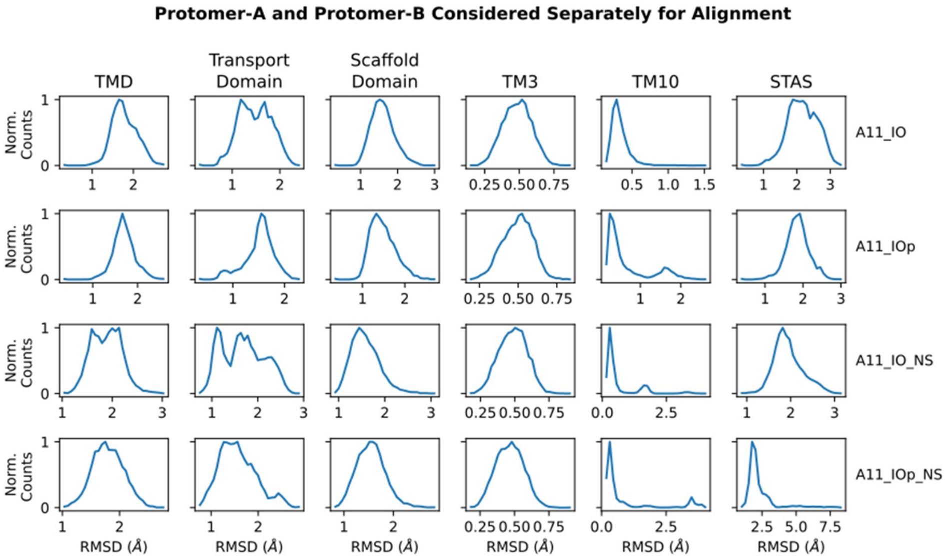

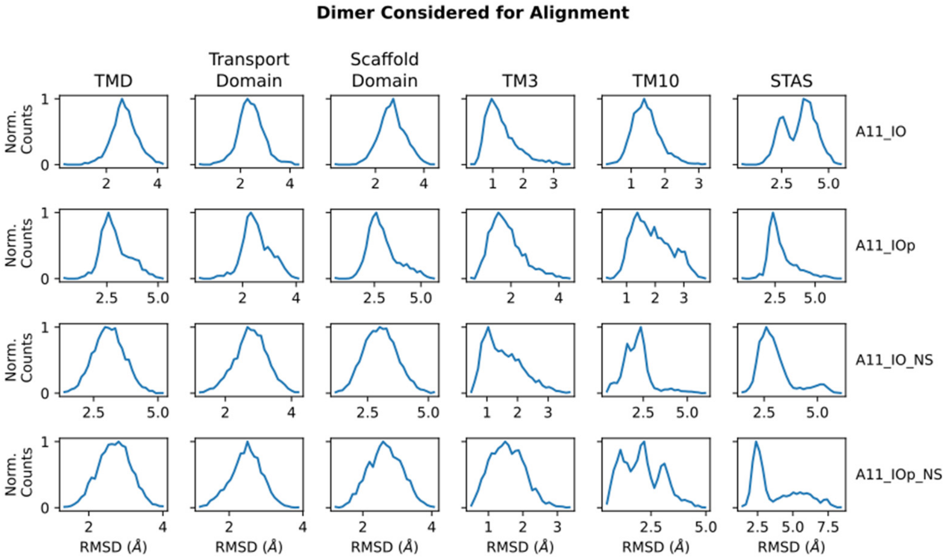

Simulation system nomenclature: inward-open with NaCl buffer (A11_IO), inward-open protonated with NaCl buffer (A11_IOp), inward-open with NaSO4 buffer (A11_IO_SO4), inward-open protonated with NaSO4 buffer (A11_IOp_SO4).

Our RMSD analyses that focus on intradomain dynamics of individual protomers (top panel) show that the inward-open structure is largely stable throughout the majority of the simulations, with the TD, SD, TMD and STAS maintaining mean RMSDs below 2 Å. When focused on TM3 and TM10—the TMs which primarily form the anion binding pocket—the mean RMSDs are around 0.2-0.5 Å. There are a few exceptional cases where TM10 begins to unravel (RMSD > 3 Å); however this is only seen in 13% of the IOp_SO4 simulation.

Our RMSD analyses of SLC26A11 dimers, which focus on changes in quaternary structure, show the same overall picture; however, these RMSDs are shifted to 2.5–3 Å. As the protomers themselves are quite stable, this increase in RMSD is due to relative motions of the protomers with respect to each other—within the simulated time, there are no clear time-resolved correlations between the RMSD traces of individual protomers, thus each protomer is generally considered as independent sample. Such dynamics are expected as the lipid-environment at 310 K is significantly more dynamic than at cryogenic temperatures, allowing the protomers to gently separate from each other while largely maintaining dimeric interfaces.

Overall, the observed intrinsic dynamics within the protomers and the slightly increased RMSDs observed for the dimer suggest that the cryo-EM structure is stable, and that we do not observe anion-dependent conformation-changes.

Obtaining a quantitative description of the kinetics and thermodynamics of SLC26A11 translocation, the coupling between ion binding and protein-conformational changes as well as an integrative understanding of how translocation and anion-conduction are coupled will require longer-timescale MD simulation, ideally combined with enhanced-sampling MD simulations; however, this would be beyond the scope of our current manuscript and will be an important topic of future investigations.

Reviewer #3 (Remarks to the Author):

Summary
The manuscript by Kuhn et al., report human SLC26A11 (KBAT) is a dual-function membrane protein: a lysosomal sulfate transporter that also exhibits a channel-like chloride conductance gated by proton:sulfate symport. A cryo-EM structure of SLC26A11 is presented, complemented by biochemical transport assays, electrophysiology, and molecular dynamics simulations. The authors propose that SLC26A11 acts as a sulfate/H+/chloride electroneutral exchanger, has channel-like activity and may represent the elusive lysosomal sulfate exporter.

Major Comments
1. Use of C-terminally truncated SLC26A11- the authors employed a C-terminally truncated construct of SLC26A11 for both structural and functional analyses. Prior studies on SLC26A9—a closely related family member—show that its C-terminus acts as an autoinhibitory gate regulating transport activity (see: Zhang et al., Cell Discovery, 2020). It would be valuable for the authors to:

- Provide a sequence alignment comparing the C-terminal regions of SLC26A11 with SLC26A9 and other SLC26 family members.

- Discuss whether a similar autoinhibitory mechanism might exist in SLC26A11 and test experimentally or at least discuss.

We assume the reviewer meant to refer to Chi et al., Cell Discovery, 2020 (PMID 32818062). The authors of this study had already mentioned poor sequence conservation in the carboxy-termini of SLC26 proteins (see cut-out below and Suppl. Fig. 4 of Chi et al., 2020) and we can only confirm this.

> **C-terminal sequence as a gating modulator**
> In our full-length structure, we observed an unexpected density located in the intracellular pocket lined by TM5, 8, 10, and 12. Further analysis shows that this density belongs to the C-terminal sequence (Fig. 2a). Sequence similarity analysis revealed poor conservation, indicating that the C-terminal sequence feature is unique to mammalian SLC26A9 (Supplementary Fig. S4). Because of the missing structure of the linkage sequence, we cannot determine which protomer the C-terminal sequence comes from.

Chi, X., Jin, X., Chen, Y. et al. Structural insights into the gating mechanism of human SLC26A9 mediated by its C-terminal sequence. Cell Discov 6, 55 (2020). https://doi.org/10.1038/s41421-020-00193-7
Licensed under CC BY 4.0 (http://creativecommons.org/licenses/by/4.0/)

Section from Chi et al., 2020

Please find a sequence alignment of all human SLC26 proteins attached at the end of this document. We do, however, note that a poor sequence conservation in the C-terminus not necessarily precludes a similar autoinhibitory mechanism as the inward-facing cavity of the SLC26A9 membrane domain, which interacts with the carboxy-terminus, is also not identical to that of SLC26A11. We therefore further investigated this option.

The size of the C-terminus, as measured starting after the last α-helix of the STAS, greatly differs between both proteins: it measures 50 amino acids for human SLC26A9 (791-741) and only 23 for human SLC26A11 (606-583).

We do not expect that SLC26A11 features an autoinhibitory mechanism similar to that of SLC26A9 for two reasons. First, our thermal unfolding experiments (Suppl. Fig. 1F) suggest that, compared to the truncation mutant, the full-length protein is not stabilized as may be expected for an inhibited state. Second, the experimental structures of SLC26A9 (PDB:7CH1) and SLC26A11 superimpose reasonably well (sequence alignment score = 735.7; RMSD between 315 pruned atom pairs is 1.125 angstroms; (across all 521 pairs: 4.651)). The last alpha-helix of their STAS domains is in a very similar position and in the same orientation. In SLC26A9, the distance between the last resolved amino acid of the STAS domain (Ala-741) and the first amino acid of the amphipathic helix that modulates activity (Asp-773) is covered by 31 amino acids. In SLC26A11, the last resolved amino acid of the STAS domain is Glu-583. This leaves only 22 amino acids to bring the terminal residue of SLC26A11 (Ala-606) in the same position. Thus, should the C-terminal residues of SLC26A11 follow a similar path as those of SLC26A9, they would cover only 71% of the distance. Finally, we note that, unlike SLC26A9, AF3 predictions for SLC26A11 do not support a position of the C-terminus near the membrane domain (figure below).

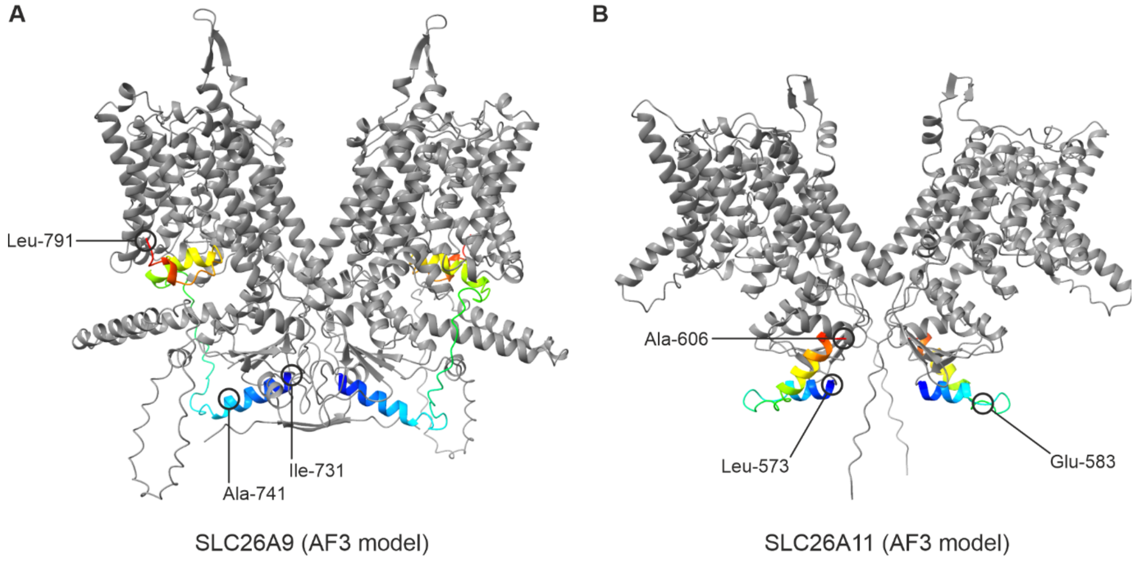

Alphafold3 models of SLC26A9 and -A11. The amino acid sequences comprising the last α-helix of the STAS domain up to the carboxy-terminus are shown in rainbow coloring.

We cannot, however, firmly exclude that the C-terminus of SLC26A11 is involved in any modulatory activity. While we agree that this is an important topic, we feel that addressing this particular aspect, one on which we do not make any claims here, falls outside of the scope of the current manuscript.

- Better explain why the full-length construct could not be used. It is mentioned that the truncated version had higher expression and was more stable, but no data provided.

In addition to the reduced expression levels and purification yields, the purification of SLC26A11^{FL} was further complicated by incomplete proteolytic cleavage of the C-terminal GFP fusion. We have added this information to Supplementary Figure 1E (depicted below).

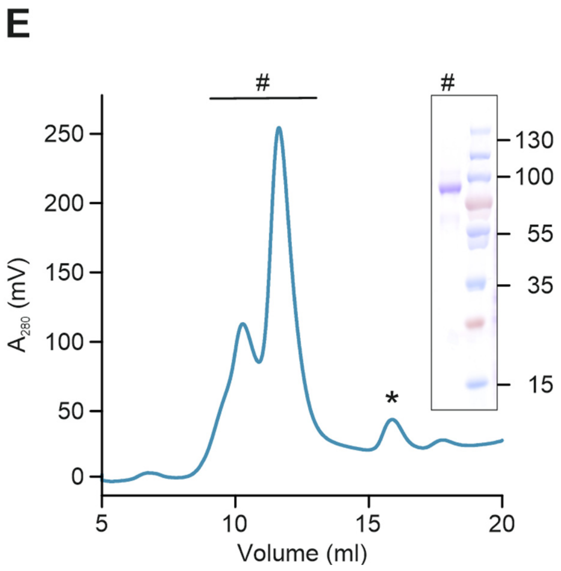

(E) Size exclusion of detergent-solubilized, IMAC-purified and HRV 3C protease-treated SLC26A11(WT) on a Superdex 200 increase 10/300 and SDS-PAGE of pooled fractions. Note the inefficiency of the 3C protease-treatment in cleaving the C-terminal GFP fusion protein as indicated by the small SEC peak at an elution volume of 16 ml (*) and the apparent migration position of the protein on SDS-PAGE.

Because our nanodisc reconstitution for cryo-EM sample preparation heavily relied on the enrichment of SLC26A11-filled nanodiscs via affinity chromatography and subsequent on-column cleavage for elution, we chose to perform structural characterization and consistently all biochemical experiments using SLC26A11$^{\Delta C}$.

We have updated the respective section in the Methods to mention the difference in 3C cleavage efficiency (line 490-492)

The SLC26A11(ΔC)-GFP fusion protein was cleaved off for 1 h at 4 °C upon addition of 500 µg HRV-3C protease. In contrast to full-length SLC26A11, the 3C site in SLC26A11(ΔC)-GFP was efficiently cleaved under these conditions.

The data supporting our claims can be found in Supplementary Fig. 1D-E (“excellent biochemical behavior”) and Supplementary Fig. 15 (“higher expression levels”). We could not find any claim in our manuscript to which the reviewer referred, stating that the truncation mutant “was more stable”. Our nanoDSF data (Supplementary Fig. 1F) suggest that both proteins are equally stable.

The full-length protein appears to be more stable as a monomer, can the authors discuss this.

The reviewer refers to Suppl. Fig. 1D-E and highlights that, in their detergent-solubilized state, the full-length protein is more stable as a monomer, or, in other words: that the full-length protein is less stable as a dimer. While we agree that the respective size-exclusion chromatograms show different fractions of monomers and dimers, we do not wish to claim any changes in the oligomeric state between the truncation mutant and the full-length protein based on the data shown. First of all, this would require a more systematic analysis with replicates as we have performed previously (Kuhn et al., 2024). In addition, any shift in the population of oligomeric states in detergent does not imply that a similar shift will also occur once the protein is embedded in the membrane (where diffusion is limited to two dimensions, and, consequently, much higher protein concentrations are reached). It is for these reasons that we respectfully prefer to refrain from commenting on these data.

2. Cryo-EM data processing and resolution improvement
- The authors report two cryo-EM reconstructions at 2.8 Å and 3.2 Å resolution. Since SLC26A11 is a

homodimer, it would be informative to know whether C2 symmetry expansion and focused refinement on a single protomer was attempted to further improve local resolution.

We explored this suggestion from the reviewer and found little improvement for the Nb4 dataset and no big effect for the Nb11 dataset. Please find the respective summary slides below. A detailed analysis is attached at the end of this document. We have deposited these datasets in the EMDB:

- EMD-56651: Sulfate transporter SLC26A11 in nanodiscs with nanobody Nb4, local refinement
- EMD-56650: Sulfate transporter SLC26A11 in nanodiscs with nanobody Nb11, local refinement

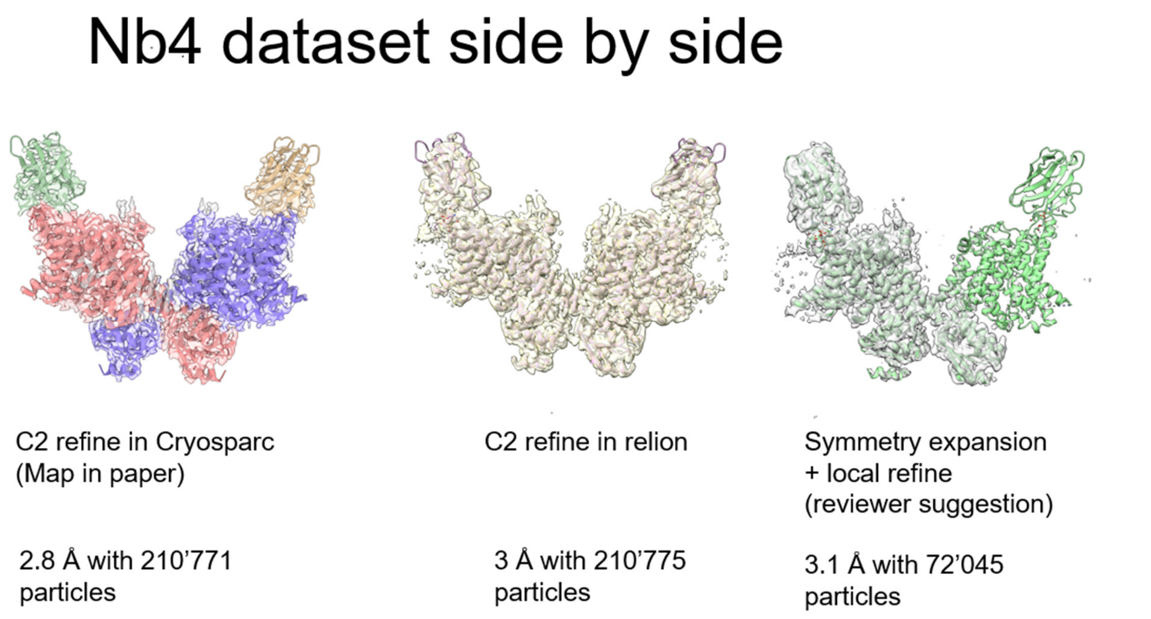

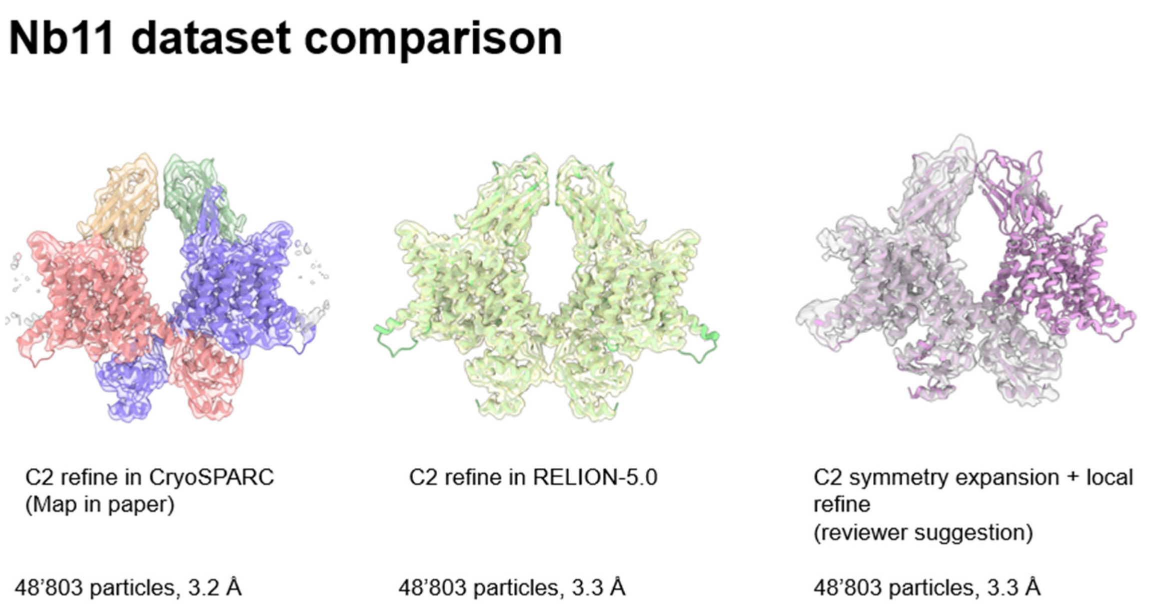

- Have the authors considered particle polishing in RELION to enhance particle alignment and map quality? Including such details would strengthen the methodological rigor.

Our experience with particle polishing is that it tends to not lead to any significant improvement so we usually do not perform it.

3. Protein purification and sample characterization
- In Supplementary Fig. 1D and 1E, it is unclear whether the SEC traces correspond to the protein after detergent solubilization or nanodisc reconstitution. The authors should clearly specify this and provide the final SEC profile of the nanodisc-reconstituted sample used for cryo-EM.

The SEC traces in these panels result from detergent-solubilized protein. This may have been revealed by the SDS-PAGE gel in panel D of Suppl. Fig. 1 that only shows a band for SLC26A11 but not an additional band for MSP1E3D1 (~33 kDa). In the revised manuscript, we now clearly state that this concerns detergent-solubilized protein.

***(D)** Size exclusion of detergent-solubilized SLC26A11(ΔC) on a Superdex 200 increase 10/300 column and SDS-PAGE of elution fractions showing the monomeric and dimeric species of SLC26A11(ΔC) (#) and the cleaved C-terminal GFP (*). **(E)** Size exclusion of detergent-solubilized SLC26A11(WT) on a Superdex 200 increase 10/300. **(F)**Thermal unfolding of detergent-solubilized SLC26A11(WT) and SLC26A11(ΔC).*

In addition, in the new panel H of Suppl. Fig. 1 (depicted below) we now provide a SEC profile of the nanodisc-reconstituted sample used for cryo-EM as requested by the reviewer.

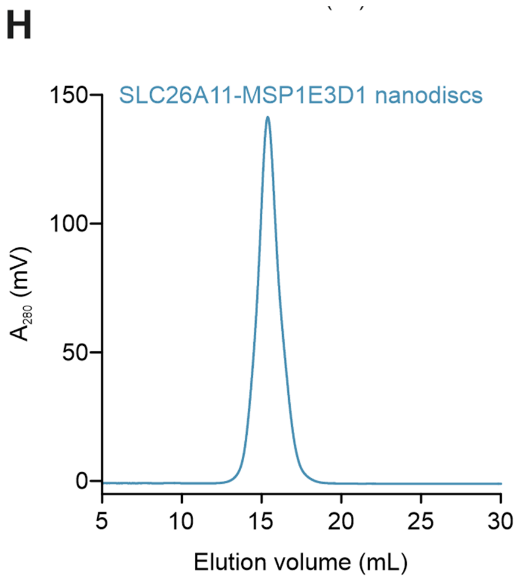

(H) Size exclusion of SLC26A11(ΔC)-MSP1E3D1 nanodiscs on a Superose 6 increase 10/300 column. The sample was injected twice to improve homogeneity. Shown is the re-injected size exclusion fraction of the first SEC run. The center of the peak was used for single-particle cryoEM.

- Please clarify whether only the dimer peak was concentrated and used for cryo-EM grid preparation.

Our sample preparation procedure is specified in the Materials/Methods section (line 475-493). We indicate there that we started the nanodisc-reconstitution procedure with protein purified by affinity chromatography. This implies that we have a mixture of monomers and dimers.

4. Substrate binding and buffer conditions. The authors purified the protein in NaCl-containing buffer. Was purification in a sulfate-containing buffer tested? This might help visualize bound substrate density in the cryo-EM map and support their proposed substrate-binding site.

We did not explore sulfate-containing buffers, but we agree with the reviewer that this may have allowed visualization of bound sulfate in our cryo-EM map.

Given the high conservation of the substrate-binding site between SLC26A11 and the *Arabidopsis thaliana* sulfate transporter SULTR4:1 —whose sulfate-bound structure has been published— we assigned a lower priority to the characterization of sulfate coordination in SLC26A11 compared to the chloride-bound structure. We agree with the reviewer that a chloride-free purification would have been ideal for this experiment, and we would like to add that sample preparation using acidic pH could further enhance sulfate binding to the protein for structural characterization.

While the suggested experiment may have supported our claim on the location of the substrate-binding site, we respectfully note that the proposed site is in full agreement with experimental structures of other liganded proteins part of the same fold. Furthermore, we show a strong change in sulfate binding affinity upon mutating Glu-320 that flanks this respective site (Fig. 4D-F). Together, we feel this leaves little doubt on our assignment of the location for the substrate-binding site.

5. The manuscript refers to “cis” and “trans” sides in transport assays without defining them. These terms should be clearly explained (e.g., which side corresponds to the lumenal vs. cytosolic compartment).

Throughout our manuscript, we consistently refer to *cis* (latin: on this side) as the compartment where (radioactive) sulfate is present and to *trans* (latin: on the other side) as the other compartment. Given that we measure uptake of sulfate into the proteoliposomes, this implies that *cis* is outside and *trans* is the lumen of the proteoliposomes.

We have explained the term *trans* upon its first mentioning (line 89).

We observed that sulfate accumulation was strongly dependent on the presence of chloride ions in trans, i.e., in the opposite compartment.

In the way we have setup our *in vitro* transport assay in Fig. 1A, the applied sulfate and chloride gradients mimic the driving forces that the protein would experience if the outside compartment was the lysosomal lumen (pH 5.0) and the inside compartment was the cytoplasm (pH 7.5).

In the revised manuscript, we have explicitly mentioned this in the legend of Fig. 1.

Figure 1: Sulfate transport of SLC26A11 is pH and chloride dependent. (A) *Time-dependent sulfate uptake into SLC26A11 proteoliposomes in presence of a pH gradient (pH 7.5 inside, pH 5.0 outside) and 50 mM internal chloride (blue) or 50 mM gluconate as chloride substitute (dark grey) and empty liposome control (light grey). In this particular configuration, the internal and external compartments mimic the cytoplasm and the lysosomal lumen, respectively.*

6. In Fig. 1B, the background level of uptake (empty liposomes) should be shown and here (and elsewhere) a t-test is not appropriate as you are comparing multiple data sets to one (symmetrical pH 7.5). An Anova with an appropriate post-hoc would be more appropriate.

Concerning the data shown, we respectfully disagree and feel that the current representation is most clear. We note that Fig. 1A, which depicts the background level of uptake over time, clearly demonstrates the (very good) signal to noise ratios of these experiments. For the background levels in the other panels, we kindly refer to the Source Data File.

Concerning the applied statistical test, we thank the reviewer for pointing out that an Anova is more appropriate. We have performed a one-way ANOVA followed by a Tukey test for data shown in figures 1B and 1E and adjusted the figure legend accordingly:

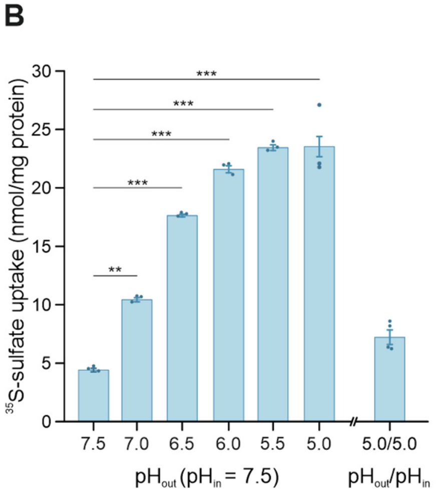

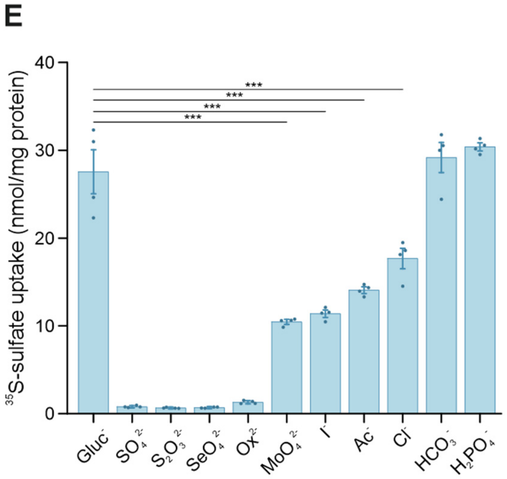

*(B) pH dependence of sulfate transport in presence of 50 mM internal chloride and with internal and external pH as indicated below the individual bars. A one-way ANOVA followed by a Tukey test was performed (*** indicates $p < 0.0001$, ** $p < 0.001$, all p values are shown in the Source Data file). (C) Sulfate transport kinetics. Data represents background-corrected initial sulfate uptake after 20 seconds in presence of increasing sulfate concentrations. Km and Vmax were derived from nonlinear curve fitting using the Michaelis-Menten function in OriginPro. (D) Chloride dependence of sulfate transport with internal and external 50 mM chloride or 50 mM gluconate as chloride substitute as indicated below the individual bars. (E) Inhibition of sulfate transport in presence of different anions as indicated below individual bars. Anions were added as sodium salt at 100-fold the concentration of external sulfate. A one-way ANOVA followed by a Tukey test was performed (*** $p < 0.0001$, all p values are shown in the Source Data file).*

7. Were the electrophysiology experiments in SF9 performed on the full length or truncated SLC26A11? The manuscript suggests these experiments were performed on WT.

We apologize for not having provided this information in the original version. All electrophysiological experiments to characterize the chloride channel activity in Sf9 insect cells were performed with the truncated version of SLC26A11. We initially compared the surface expressions of truncated and full-length versions by confocal imaging and electrophysiology. The truncated version showed higher membrane expression levels (Supplementary Fig. 15) and higher currents than the full-length version.

We have updated the legend of Fig. 5 to clearly reflect that the truncated version of the protein was analyzed.

Figure 5: SLC26A11 exhibits pH- and sulfate-dependent intrinsic chloride channel activity. (A) *Representative current recordings from Sf9 cells expressing SLC26A11$^{\Delta C}$-eGFP with buffer conditions as shown in panel B.* ***(B)*** *Current-voltage relationships (means ± standard errors; n = 13/11/7) from transfected Sf9 cells in three different bath solutions as indicated (grey: 0 mM sulfate, pH 7; blue: 10 mM sulfate, pH 7; red: 10 mM sulfate, pH 6.0).* ***(C)*** *Current-voltage relationships (means ± standard errors; n = 9/10) from untransfected Sf9 cells in the same solutions as indicated in panel B.* ***(D, E)*** *Representative whole-cell current traces* ***(D)*** *and current-voltage relationships (mean ± standard errors; WT: n = 29/6, E320Q: n = 25/11)* ***(E)*** *for SLC26A11$^{\Delta C}$(WT) and SLC26A11$^{\Delta C}$(E320Q) in absence or presence of a pH gradient (blue: symmetrical pH 7.33; red: pH_{out} 6.00, pH_{in} 7.33).* ***(F)*** *upper panel: Reversal potentials (U_{rev}) for SLC26A11$^{\Delta C}$(WT) at different external pH values presented as whisker-box plots. Boxes indicate upper and lower 75/25% quartiles with medians. Whiskers span 95% confidence limits. Data points are derived from experiments shown in panel D and E and Supplementary Fig. 19. lower panel: Current amplitudes of SLC26A11$^{\Delta C}$(WT) and SLC26A11$^{\Delta C}$(E320Q) at -120 mV at experimental conditions as shown in panel E at various external pH conditions (pH 7.33/7.00/6.66/6.33/6.00; WT: n =29/20/12/7/6, E320Q: n = 25/15/12/12/11).*

Did the authors compare the function of full-length to the truncated form that was used for the proteoliposome experiments?

No, we did not directly compare the full-length and the truncated forms in functional assays.

8. In Fig. 6 there is a statement that “Representations of SLC26A11 with the substrate binding site open to the lysosomal lumen or with a channel-like configuration are toy models”. While I appreciate the authors are pointing out these are not real structures, this could be consuming to the reader and make-believe structures with a continuous pore (such as in step *) should not be represented in structure form – if a structure that doesn’t exist. Cartoon representation should be used in the absence of structural information. Can the open-to-the-lumen side not be modelled from structures of other SLC26 members in a similar state?

The outward-open conformations shown in Fig. 6 are modelled on the basis of the SLC26A11 Alphafold2 model AF-Q86WA9-F1-v6. In our revised manuscript, we have now clearly stated this in the legend of Fig. 6.

Representations of SLC26A11 with the substrate binding site open to the lysosomal lumen are based on an outward-open, monomeric SLC26A11 Alphafold2 model (AF-Q86WA9-F1-v6) that was converted into an asymmetric dimer through structural alignment with an inward-open, dimeric SLC26A11 structure. The representation of SLC26A11 with a channel-like configuration is a toy model.

In addition, we have updated the representation of the continuous pore state to reflect that it is not based on an actual structure, while still highlighting key features such as the bound sulfate ion, the deprotonated state of the central glutamate, and the anticipated location of the conductance path (see below).

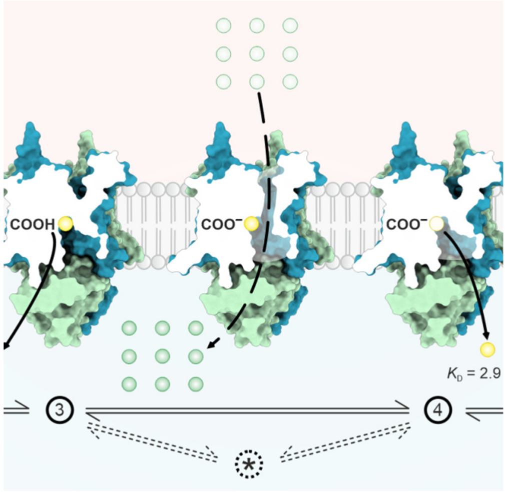

Previous version

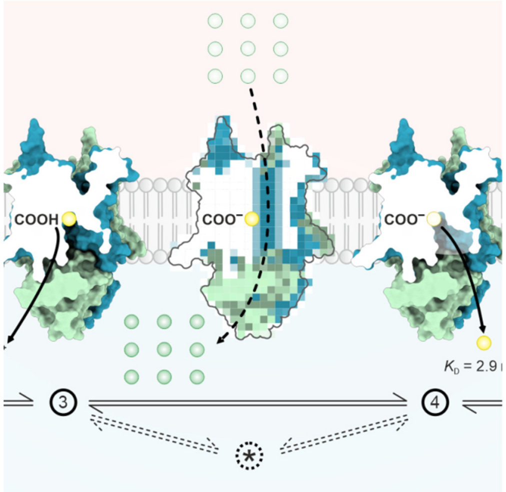

Revised version

9. The comparison to the dual function EAATs where the chloride conductance is uncoupled should be softened. There have been many studies over a long time that have demonstrated this process is uncoupled and the nature of the conductance studied in detail. This situation is not the same as chloride is coupled to the transport/exchange process and the experiments to determine whether these processes are uncoupled have not been performed.

We respectfully disagree and believe that the comparison with uncoupled chloride conductance is highly appropriate.

SLC26A11 differs from the EAATs in that chloride ions are a substrate in the coupled transport in SLC26A11 ($H^+:SO_4^{2-}/Cl^-$), whereas chloride ions are not a substrate in the coupled transport of EAATs ($3Na^+:H^+$:glutamate/K^+).

However, like the EAATs, SLC26A11 additionally exhibits a thermodynamically uncoupled conductance of chloride ions. This enables SLC26A11 to perform both carrier-mediated chloride transport and channel-mediated chloride fluxes. We have demonstrated uncoupled chloride conductance through the heterologous expression of SLC26A11 in insect cells and whole-cell patch clamp recordings. In this system, we observe SLC26A11-dependent currents **[Fig. 5]**. Such currents are not expected for an electroneutral transporter without uncoupled conductance. These currents reverse close to the chloride equilibrium potential **[Supplementary Figure 18]**. The current reversal potential does not change with variation in the sulfate concentration **[Fig. 5B]** or pH **[Fig. 5E, Supplementary Figure 17B]**. These currents therefore fulfil all the criteria of passive transport. They are not mediated by the coupled transport function of SLC26A11 ($H^+:SO_4^{2-}/Cl^-$), but by an additional uncoupled mode.

In the following section in the discussion, we detail why we propose that this uncoupled transport mode is due to the formation of an anion channel (line 356-366). We have reworded this section slightly to avoid any misconception that the chloride conductance emerges from a conventional alternating-access transport scheme.

In principle, passive currents can be conducted by ion channels, but also by transporters, i.e., by chloride uniporters. Since SLC26A11 functions as a proton:sulfate/chloride exchanger, a possible explanation for conductive chloride transport is a variation of the transport cycle resulting in chloride uniport. For example, after chloride translocation, the transporter could return to its initial conformation in the apo state instead of the proton- and sulfate-bound state. However, the experimentally observed enhancement of chloride currents by conditions favoring sulfate transport ***[Fig. 5; Supplementary Fig. 17]*** *is incompatible with this uniport mode of chloride transport. The co-existence of secondary active proton:sulfate/chloride exchange and passive chloride currents activated by sulfate transport leaves little alternatives to a channel-like conducting state in SLC26A11.*

It is also not clear that the authors have demonstrated "the importance of chloride in accelerating the transport mode" (line 382) which appears to be referring to the channel activity as chloride is required for the transport process.

The respective statement does not refer to the channel activity, but to the transport activity instead. We have demonstrated the relevance of *trans* chloride in accelerating *cis* sulfate:proton uptake in Fig. 1A.

In the revised manuscript, we now refer to the respective figure to avoid this misconception (line 390-391).

Given the importance of chloride in accelerating the transport mode ***[Fig. 1A]****, this feedback would ensure high transport rates.*

10. In general, n values are low (Supp Table 1) and the valinomycin experiment in Supp Fig 2b should show initial rates of transport rather than uptake at 16 min. Membrane potentials generated by the addition of valinomycin indicated on the graph.

With respect to the perceived low number of replicates, we note that the datapoints in all panels of Fig. 1 show only a small spread and that the respective error bars are barely visible for several datapoints. We thus feel that the current number of replicates suffices to support our claims.

With respect to the valinomycin experiment, we respectfully disagree and refer to the discussion below and Fig. 1D from Geertsma et al., 2015 (PMID: 26367249; ref. 13; shown below for your convenience) that indicates that both the initial rate and the final accumulation levels can be used to study the effect of the membrane potential.

Based on the suggestion of the reviewer, we have modified Suppl. Fig. 2B to indicate the magnitude of the membrane potential as well (see below).

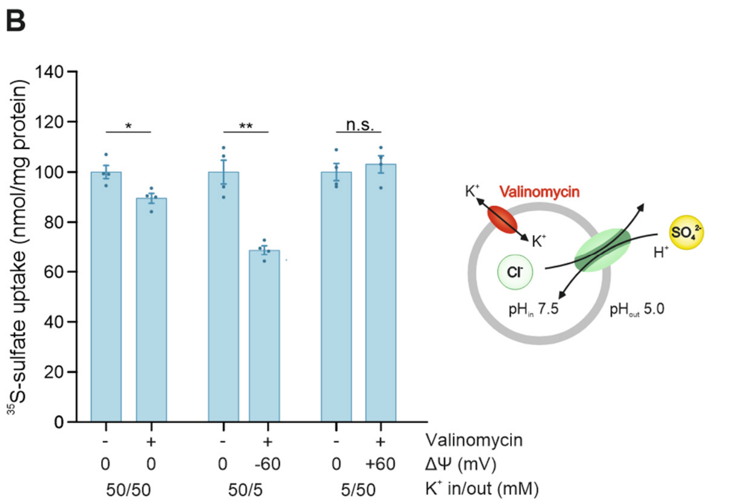

11. The statement that "For electrogenic transport, a more pronounced reduction at -60 mV and a concomitant increase in sulfate accumulation at +60 mV would be expected" is not backed up by the data. There is a significant reduction -60mV and without initial rates of transport, it is difficult to interpret what is going on here.

We refer here to Fig. 1D from Geertsma et al., 2015 (PMID: 26367249; ref. 13). We depict the respective panel below for your convenience. This protein (SLC26Dg) is clearly electrogenic: we observe a strong reduction in initial transport rate <u>and final accumulation level</u> in the presence of an inside-negative membrane potential, while we observe the opposite for an inside-positive membrane potential: a strong increase in initial transport rate <u>and final accumulation level</u>.

Reproduced from Geertsma, E., Chang, YN., Shaik, F. et al. Structure of a prokaryotic fumarate transporter reveals the architecture of the SLC26 family. Nat Struct Mol Biol 22, 803–808 (2015). https://doi.org/10.1038/nsmb.3091

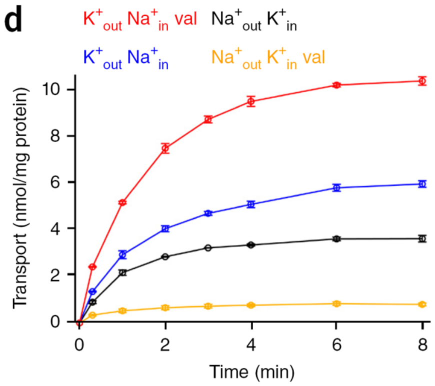

addition of nigericin. Individual traces of duplicate experiments are shown. (**d**) Dependence of ^{14}C-fumarate (50 μM) uptake on the membrane potential. 'K^+_{out} Na^+_{in} val' refers to 50 mM of K^+ outside and 50 mM Na^+ inside the liposome, which generates a positive membrane potential upon addition of valinomycin. This ratio is inverted in 'Na^+_{out} K^+_{in} val'. 'K^+_{out} Na^+_{in}' and 'Na^+_{out} K^+_{in}' refer to conditions in which no valinomycin was added. Data in **a**, **b** and **d** represent mean and s.e.m. of 3 technical replicates.

Clearly, the magnitude of the membrane potential-induced change in transport rate and accumulation level observed for SLC26Dg is very different from the 30% drop that we observe here for SLC26A11. In addition, we do not observe any increase in accumulation level when we apply a membrane potential with the opposite direction.

In a more formal explanation, we can predict how the sulfate accumulation level would be affected in a simple, ideal system if SLC26A11 was electrogenic, e.g., a proton:sulfate symporter resulting in the net uptake of one elementary charge per transport cycle. In that case, the equilibrium concentrations of protons and sulfate will depend on each other with

$$\frac{[SO_4{}^{2-}]_{in}}{[SO_4{}^{2-}]_{out}} = \frac{[H^+]_{out}}{[H^+]_{in}} e^{\frac{UF}{RT}}$$

Using this formula, we expect the steady-state sulfate accumulation to decrease by a factor of 0.1 from 0 mV to -60 mV and to increase by a factor of 10 (exp(60 mV*F/RT) from 0 mV to +60 mV.

In contrast, there will be no voltage dependence for electroneutral H^+:SO_4^{2-}/Cl^- exchange.

While we stand by our conclusion that these data are best explained by an electroneutral mode of transport, we agree that the 30% reduction is difficult to interpret.

We have updated the text to reflect that we do not fully understand this behavior (line 127-135).

We observed only modest changes when generating a membrane potential of 0 and +60 mV, but at -60 mV sulfate accumulation was reduced by approximately 30%. For electrogenic transport, a more pronounced reduction at -60 mV and a concomitant increase in sulfate accumulation at +60 mV would be expected, as shown for the proton:fumarate symporter SLC26Dg [13]. Though the reduced accumulation levels at -60 mV resist simple interpretation, these results together support an electroneutral transport mode for SLC26A11. In the most parsimonious model, SLC26A11 is an exchanger that couples the symport of one proton and one sulfate ion (net charge of -1) to the antiport of one chloride ion (net charge of -1).

Minor comments
12. Structural presentation and figure quality - the density maps shown in Supplementary Figs. 6 and 7 appear low in quality and difficult to interpret. The authors should improve the map visualization.

We agree that the quality of the respective figures in the PDF is low. This was caused by the conversion to the PDF format. In the revised version of the manuscript, we have exported our supplement at the highest resolution. We also provide the original images as files.

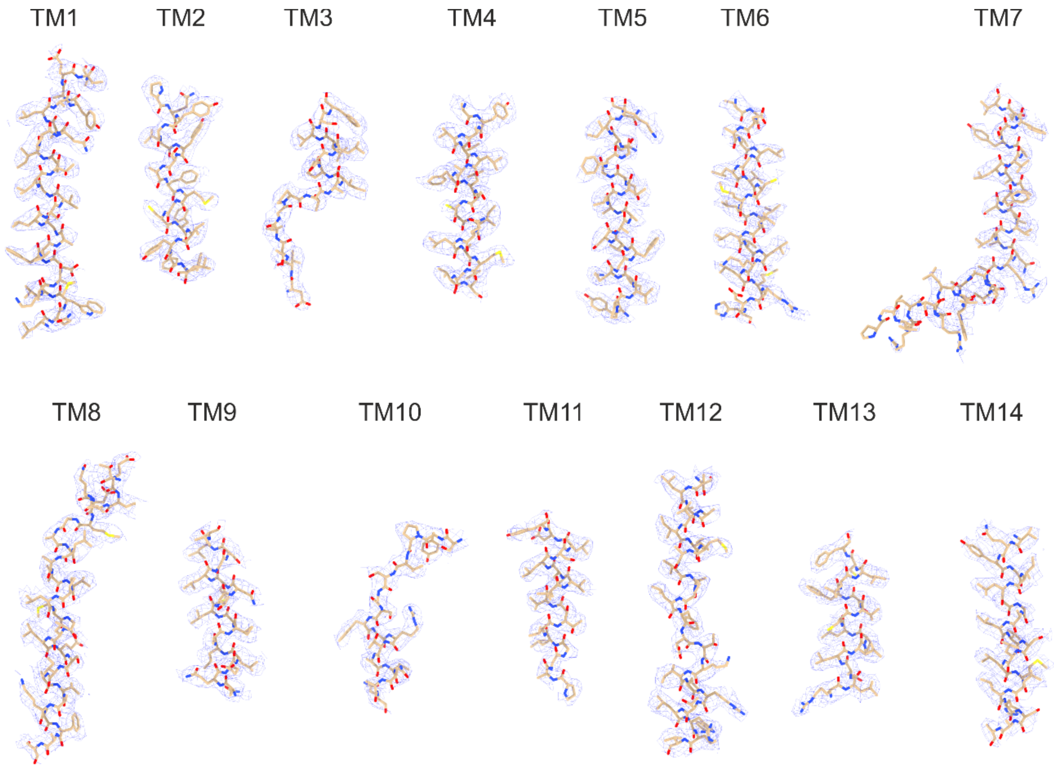

Supplementary Figure 6: Representative densities of SLC26A11-Nb11. Transmembrane segments shown as sticks with corresponding density (blue mesh).

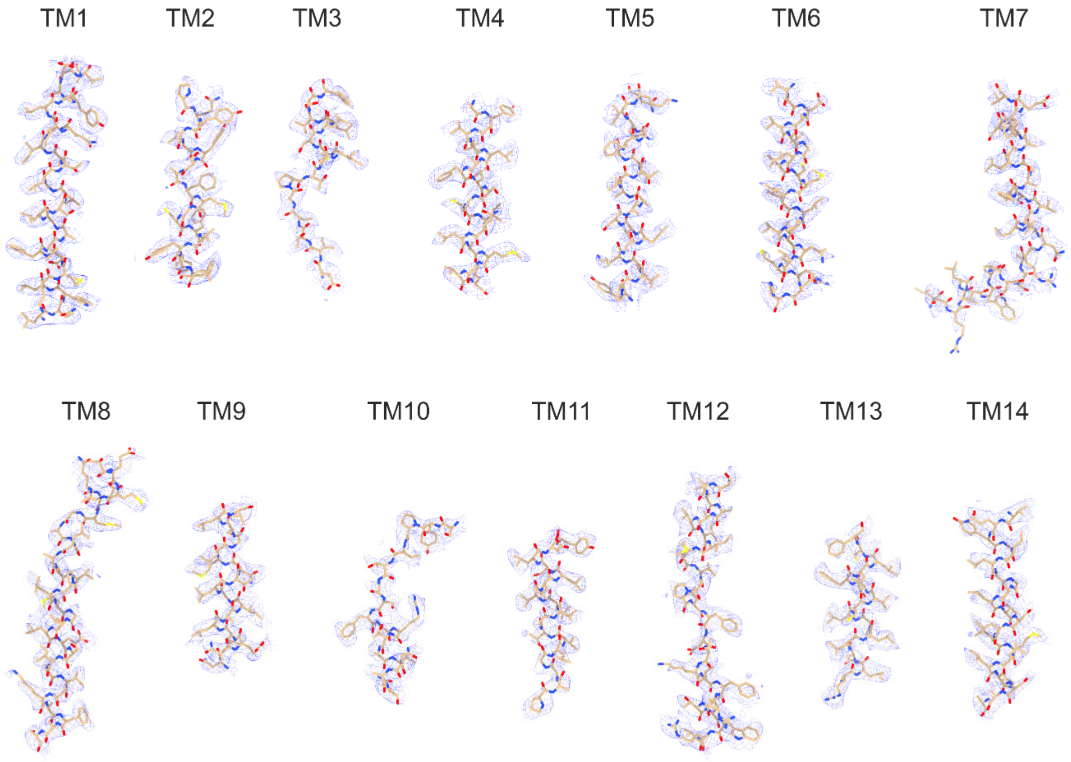

Supplementary Figure 7: Representative densities of SLC26A11-Nb4. Transmembrane segments shown as sticks with corresponding density (blue mesh).

13. On page 10, line 244, the text should reference Supplementary Fig. 13 for clarity.

In the revised manuscript we now refer to this figure as well (line 244-246).

The chloride dissociation constant of SLC26A11$^{\Delta C}$(E320Q), as determined by DSF, was comparable to that of the wild type protein at pH 5.0 (8.8 ± 1.4 mM) and was increased six-fold at pH 7.5 (30 ± 4.0 mM) ***[Fig. 4D, E; Suppl. Fig. 13]****.*

14. In Fig. 1E, explicitly state that the assay measures [35S]-sulfate uptake.

All measurements in Fig. 1 are based on [35S]-sulfate uptake. We have updated all the respective axis labels of all panels to reflect that these measurements involved [35S]-sulfate (below).

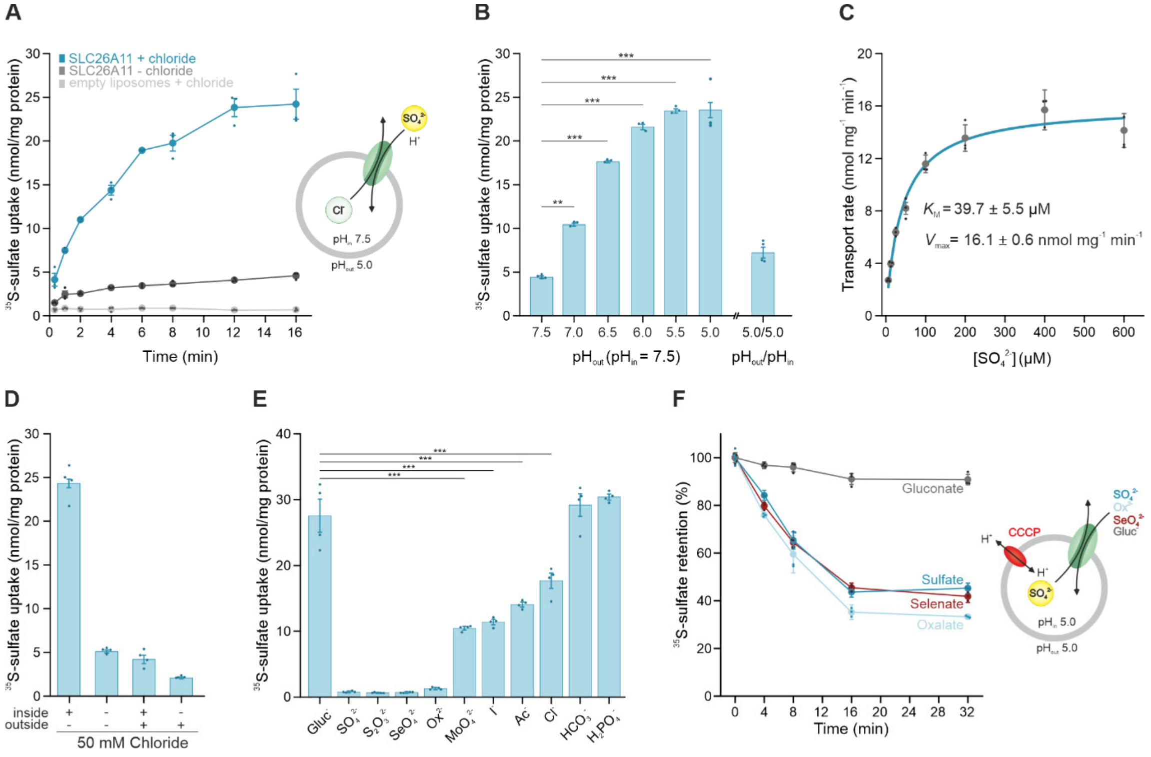

15. In Fig. 1F, include clear legend labels or color annotations to indicate what each curve represents.

We have updated the respective panel based on the suggestions of the reviewer (see above).

I co-reviewed this manuscript with one of the reviewers who provided the listed reports. This is part of the Nature Communications initiative to facilitate training in peer review and to provide appropriate recognition for Early Career Researchers who co-review manuscripts.

```
CLUSTAL O(1.2.4) multiple sequence alignment


SLC26A11               ------------------------------------------------------------   0
SLC26A08               ----------------------------MAQLERSA------ISGFSSKSRRNSFAYDVK   26
SLC26A01               -------------------------------MDESPE---PL--------QQGRGPVPVR   18
SLC26A02               MSSESKEQHNVSPRDSAEGNDSYPSGIHLELQRESST---DFKQFETNDQCRPYHRILIE   57
SLC26A09               -------------------------------------------------MSQPRPRYVVD   11
SLC26A05               -------------------------------MDH----------AEENEILAATQRYYVE   19
SLC26A06               ----------------------------MGLADASGPRDTQALLSATQAMDLRRRDYHME   32
SLC26A03               ------------------------------------------------MIEPFGNQYIVA   12
SLC26A04               -------------------------------MAAPGGRSEPP------QLPEYSCSYMVS   23
SLC26A07               ------------------------------------------------------------   0
SLC26A10_[pseudo]      ------------------------------------------------------------   0


SLC26A11               -MPSSVTAL-----GQARSSGP-------GMAPSACCCSPAA----LQRRLPILAWLPSY   43
SLC26A08               REVYNEETFQQEHKRKASSSGNMNINITTFRHHVQCRCSWHRFLRCVLTIFPFLEWMCMY   86
SLC26A01               RQRPAPRGL----------REM-----LKARLWCSCSCSVLCVRALVQDLLPATRWLRQY   63
SLC26A02               RQEKSDTNF----------KEF-----VIKKLQKNCQCSPAKAKNMILGFLPVLQWLPKY   102
SLC26A09               RAAYSLTLFDDEFEKKDR-TYP-----VGEKLRNAFRCSSAKIKAVVFGLLPVLSWLPKY   65
SLC26A05               RPIFSHPVLQERLHTKDKVPDS-----IADKLKQAFTCTPKKIRNIIYMFLPITKWLPAY   74
SLC26A06               RPLLNQEHLEELGRWGSA---P-----RTHQWRTWLQCSRARAYALLLQHLPVLVWLPRY   84
SLC26A03               RPVYSTNAFEENHKKTGRHHKT-----FLDHLKVCCSCSPQKAKRIVLSLFPIASWLPAY   67
SLC26A04               RPVYSELAFQQQHERRLQERKT-----LRESLAKCCSCSRKRAFGVLKTLVPILEWLPKY   78
SLC26A07               ------------MTGAKRKKKS-----MLWSKMHTPQC--EDIIQWCRRRLPILDWAPHY   41
SLC26A10_[pseudo]      -------------------MRL-----DLASLMSAPKSLGSAFKSWRLDKAP--------   28
                                                            .             *

SLC26A11               SL-QWLKMDFVAGLSVGLTAIPQALAYAEVAG--LPPQYGLYSAFMGCFVYFFLGTSRDV   100
SLC26A08               RLKDWLLGDLLAGISVGLVQVPQGLTLSLLARQLIPPLNIAYAAFCSSVIYVIFGSCHQM   146
SLC26A01               RPREYLAGDVMSGLVIGIILVPQAIAYSLLAG--LQPIYSLYTSFFANLIYFLMGTSRHV   121
SLC26A02               DLKKNILGDVMSGLIVGILLVPQSIAYSLLAG--QEPVYGLYTSFFASIIYFLLGTSRHI   160
SLC26A09               KIKDYIIPDLLGGLSGGSIQVPQGMAFALLAN--LPAVNGLYSSFFPLLTYFFLGGVHQM   123
SLC26A05               KFKEYVLGDLVSGISTGVLQLPQGLAFAMLAA--VPPIFGLYSSFYPVIMYCFLGTSRHI   132
SLC26A06               PVRDWLLGDLLSGLSVAIMQLPQGLAYALLAG--LPPVFGLYSSFYPVFIYFLFGTSRHI   142
SLC26A03               RLKEWLLSDIVSGISTGIVAVLQGLAFALLVD--IPPVYGLYASFFPAIIYLFFGTSRHI   125
SLC26A04               RVKEWLLSDVISGVSTGLVATLQGMAYALLAA--VPVGYGLYSAFFPILTYFIFGTSRHI   136
SLC26A07               NLKENLLPDTVSGIMLAVQQVTQGLAFAVLSS--VHPVFGLYGSLFPAIIYAIFGMGHHV   99
SLC26A10_[pseudo]      -----------SPQHTFPSTSIPGMAFALLAS--VPPVFGLYTSFFPVLIYSLLGTGRHL   75
                                  .           .:: : :           * ::   . * ::*  :.:

SLC26A11               TLGPTAIMSLLVSFYTFHEP---------------------------------------A   121
SLC26A08               SIGSFFLVSALLINVLKVS-P---FNNGQLVMGS--FVKNE----FSAPSYLMGYNKSLS   196
SLC26A01               SVGIFSLLCLMVGQVVDRELQLAGFDPSQDGLQPG--ANSSTLNGSAAMLDCGRDCYAIR   179
SLC26A02               SVGIFGVLCLMIGETVDRELQKAGYDNAHSAPSLGMVSNGSTLLNHTSDRICDKSCYAIM   220
SLC26A09               VPGTFAVISILVGNICLQLAPES----KF---QV----FNN-ATNESYVDTAAMEAERLH   171
SLC26A05               SIGPFAVISLMIGGVAVRLVP----DDIVIPGGV----NA-------TNGTEARDALRVK   177
SLC26A06               SVGTFAVMSVMVGSVTESLAP-------------------Q-ALNDSMINETARDAARVQ   182
SLC26A03               SVGPFPILSMMVGLAVSGAVSKAVPDRNATTLGL----PNN-SNNSS---LLDDERVRVA   177
SLC26A04               SVGPFPVVSLMVGSVVLSMA----PDEHFLVSSS----NGT-VLNTTMIDTAARDTARVL   187
SLC26A07               ATGTFALTSLISANAVERIVPQNM-------------QNLTTQSNTSVLGLSDFEMQRIH   146
SLC26A10_[pseudo]      STGTFAILSLMTGSAVERLVPEPL-------------VGN-----LSGIEKEQLDAQRVG   117
                         *   : . :

SLC26A11               YAVLLAFLSGCIQLAMGVLRLGFLLDFISYPVIKGFTSAAAVTIGFGQIKNLLGLQ----   177
SLC26A08               VVATTTFLTGIIQLIMGVLGLGFIATYLPESAMSAYLAAVALHIMLSQLTFIFGIMISFH   256
SLC26A01               VATALTLMTGLYQVLMGVLRLGFVSAYLSQPLLDGFAMGASVTILTSQLKHLLGVRIPRH   239
SLC26A02               VGSTVTFIAGVYQVAMGFFQVGFVSVYLSDALLSGFVTGASFTILTSQAKYLLGLNLPRT   280
SLC26A09               VSATLACLTAIIQMGLGFMQFGFVAIYLSESFIRGFMTAAGLQILISVLKYIFGLTIPSY   231
SLC26A05               VAMSVTLLSGIIQFCLGVCRFGFVAIYLTEPLVRGFTTAAAVHVFTSMLKYLFGVKTKRY   237
SLC26A06               VASTLSVLVGLFQVGLGLIHFGFVVTYLSEPLVRGYTTAAAVQVFVSQLKYVFGLHLSSH   242
SLC26A03               AAASVTVLSGIIQLAFGILRIGFVVIYLSESLISGFTTAAAVHVLVSQLKFIFQLTVPSH   237
SLC26A04               IASALTLLVGIIQLIFGGLQIGFIVRYLADPLVGGFTTAAAFQVLVSQLKIVLNVSTKNY   247
SLC26A07               VAAAVSFLGGVIQVAMFVLQLGSATFVVTEPVISAMTTGAATHVVTSQVKYLLGMKMPYI   206
SLC26A10_[pseudo]      VAAAVAFGSGALMLGMFVLQLGVLSTFLSEPVVKALTSGAALHVLLSQLPSLLGLSLPRQ   177
                            :   .   . :    .*     :    : .   ...  :  .    :: :
```

```
SLC26A11               NIPRPFFLQVYHTFLRIAETRVGDAVLGLVCMLLLLVLKLMRDHVPPVHPEMPPGVRLSR   237
SLC26A08               AGPISFFYDIINYCVALPKANSTSILVFLTVVVALRINKCIRISF-NQYPIEFPM-----   310
SLC26A01               QGPGMVVLTWLSLLRGAGQANVCDVVTSTVCLAVLLAAKELSDRYRHRLRVPLPT-----   294
SLC26A02               NGVGSLITTWIHVFRNIHKTNLCDLITSLLCLLVLLPTKELNEHFKSKLKAPIPI-----   335
SLC26A09               TGPGSIVFTFIDICKNLPHTNIASLIFALISGAFLVLVKELNARYMHKIRFPIPT-----   286
SLC26A05               SGIFSVVYSTVAVLQNVKNLNVCSLGVGLMVFGLLLGGKEFNERFKEKLPAPIPL-----   292
SLC26A06               SGPLSLIYTVLEVCWKLPQSKVGTVVTAAVAGVVLVVVKLLNDKLQQQLPMPIPG-----   297
SLC26A03               TDPVSIFKVLYSVFSQIEKTNIADLVTALIVLLVVSIVKEINQRFKDKLPVPIPI-----   292
SLC26A04               NGVLSIIYTLVEIFQNIGDTNLADFTAGLLTIVVCMAVKELNDRFRHKIPVPIPI-----   302
SLC26A07               SGPLGFFYIYAYVFENIKSVRLEALLLSLLSIVVLVLVKELNEQFKRKIKVVLPV-----   261
SLC26A10_[pseudo]      IGCFSLFKTLASLLTALPRSSPAELTISALSLALLVPVKELNVRFRDRLPTPIPG-----   232
                            ..                               * :            *

SLC26A11               GLVWAATTARNALVVSFAALVAYSFEVTGYQPFILTGETAEGLPPVRIPPFSVTTANGTI   297
SLC26A08               ----------ELFLIIGFTVIANKISMATETSQTLIDMIPYSFLLPVTPDFSLLPKI---   357
SLC26A01               ----------ELLVIVVATLVSHFGQLHKRFGSSVAGDIPTGFMPPQVPEPRLMQRV---   341
SLC26A02               ----------ELVVVVAATLASHFGKLHENYNSSIAGHIPTGFMPPKVPEWNLIPSV---   382
SLC26A09               ----------EMIVVVVATAISGGCKMPKKYHMQIVGEIQRGFPTPVSPVVSQWKDM---   333
SLC26A05               ----------EFFAVVMGTGISAGFNLKESYNVDVVGTLPLGLLPPANPDTSLFHLV---   339
SLC26A06               ----------ELLTLIGATGISYGMGLKHRFEVDVVGNIPAGLVPPVAPNTQLFSKL---   344
SLC26A03               ----------EFIMTVIAAGVSYGCDFKNRFKVAVVGDMNPGFQPPITPDVETFQNT---   339
SLC26A04               ----------EVIVTIIATAISYGANLEKNYNAGIVKSIPRGFLPPELPPVSLFSEM---   349
SLC26A07               ----------DLVLIIAASFACYCTNMENTYGLEVVGHIPQGIPSPRAPPMNILSAV---   308
SLC26A10_[pseudo]      ----------EVVLVLLASVLCFTSSVDTRYQVQIVGLLPGGFPQPLLPNLAELPRI---   279
                                 : .     :  .    .       :      .:     *

SLC26A11               SFTEMVQDMGAGLAVVPLMGLLESIAVAKAFASQNNYRIDANQELLAIGLTNMLGSLVSS   357
SLC26A08               ---------ILQAFSLSLVSSFLLIFLGKKIASLHNYSVNSNQDLIAIGLCNVVSSFFRS   408
SLC26A01               ---------ALDAVALALVAAAFSISLAEMFARSHGYSVRANQELLAVGCCNVLPAFLHC   392
SLC26A02               ---------AVDAIAISIIGFAITVSLSEMFAKKHGYTVKANQEMYAIGFCNIIPSFFHC   433
SLC26A09               ---------IGTAFSLAIVSYVINLAMGRTLANKHGYDVDSNQEMIALGCSNFFGSFFKI   384
SLC26A05               ---------YVDAIAIAIVGFSVTISMAKTLANKHGYQVDGNQELIALGLCNSIGSLFQT   390
SLC26A06               ---------VGSAFTIAVVGFAIAISLGKIFALRHGYRVDSNQELVALGLSNLIGGIFQC   395
SLC26A03               ---------VGDCFGIAMVAFAVAFSVASVYSLKYDYPLDGNQELIALGLGNIVCGVFRG   390
SLC26A04               ---------LAASFSIAVVAYAIAVSVGKVYATKYDYTIDGNQEFIAFGISNIFSGFFSC   400
SLC26A07               ---------ITEAFGVALVGYVASLALAQGSAKKFKYSIDDNQEFLAHGLSNIVSSFFFC   359
SLC26A10_[pseudo]      ---------LADSLPIALVSFAVSASLASIHADKYSYTIDSNQEFLAHGASNLISSLFSC   330
                                      : ::.      :.   :    * :  **:: * *  * . ...

SLC26A11               YPVTGSFGRTAVNAQSGVCTPAGGLVTGVLVLLSLDYLTSLFYYIPKSALAAVIIMAVAP   417
SLC26A08               CVFTGAIARTIIQDKSGGRQQFASLVGAGVMLLLMVKMGHFFYTLPNAVLAGIILSNVIP   468
SLC26A01               FATSAALAKSLVKTATGCRTQLSSVVSATVVLLVLLALAPLFHDLQRSVLACVIVVSLRG   452
SLC26A02               FTTSAALAKTLVKESTGCHTQLSGVVTALVLLLVLLVIAPLFYSLQKSVLGVITIVNLRG   493
SLC26A09               HVICCALSVTLAVDGAGGKSQVASLCVSLVVMITMLVLGIYLYPLPKSVLGALIAVNLKN   444
SLC26A05               FSISCSLSRSLVQEGTGGKTQLAGCLASLMILLVILATGFLFESLPQAVLSAIVIVNLKG   450
SLC26A06               FPVSCSMSRSLVQESTGGNSQVAGAISSLFILIIVKLGELFHDLPKAVLAAIIIVNLKG   455
SLC26A03               FAGSTALSRSAVQESTGGKTQIAGLIGAIIVLIVVLAIGFLLAPLQKSVLAALALGNLKG   450
SLC26A04               FVATTALSRTAVQESTGGKTQVAGIISAAIVMIAILALGKLLEPLQKSVLAAVVIANLKG   460
SLC26A07               IPSAAAMGRTAGLYSTGAKTQVACLISCIFVLIVIYAIGPLLYWLPMCVLASIIVVGLKG   419
SLC26A10_[pseudo]      FPNSATLATTNLLVDAGGKTQLAGLFSCTVVLSVLLWLGPFFYYLPKAVLACINISSMRQ   390
                            ::. :     :*     .      .::  :      :  :  ..*. :    :

SLC26A11               LFDT-KIFRTLWRVKRLDLLPLCVTFLLCF-WEVQYGILAGALVSL----LMLLHSAARP   471
SLC26A08               YLETISNLPSLWRQDQYDCALWMMTFSSSIFLGLDIGLIISVVSAF----FITTVRSHRA   524
SLC26A01               ALRKVWDLPRLWRMSPADALVWAGTAATCMLVSTEAGLLAGVILSL----LSLAGRTQRP   508
SLC26A02               ALRKFRDLPKMWSISRMDTVIWFVTMLSSALLSTEIGLLVGVCFSI----FCVILRTQKP   549
SLC26A09               SLKQLTDPYYLWRKSKLDCCIWVVSFLSSFFLSLPYGVAVGVAFSV----LVVVFQTQFR   500
SLC26A05               MFMQFSDLPFFWRTSKIELTIWLTTFVSSLFLGLDYGLITAVIIAL----LTVIYRTQSP   506
SLC26A06               MLRQLSDMRSLWKANRADLLIWLVTFTATILLNLDLGLVVAVIFSL----LLVVVRTQMP   511
SLC26A03               MLMQFAEIGRLWRKDKYDCLIWIMTFIFTIVLGLGLGLAASVAFQL----LTIVFRTQFP   506
SLC26A04               MFMQLCDIPRLWRQNKIDAVIWVFTCIVSIILGLDLGLLAGLIFGL----LTVVLRVQFP   516
SLC26A07               MLIQFRDLKKYWNVDKIDWGIWVSTYVFTICFAANVGLLFGV----VCTIAIVIGRFPRA   475
SLC26A10_[pseudo]      VFCQMQELPQLWHISRVDFLLQVPGLCILS---YPTPLYFGTRGQFRCNLEWHLGLGEGE   447
                        :         *  .  :                   :  .

SLC26A11               ETKVS--------------------EGPVLVLQPASGLSFPAMEALREEIL---------   502
SLC26A08               KILLLGQIPNTNIYRSINDYREIITIPGVKIFQCCSSITFVNVYYLKHKLLKEVDMVKVP   584
SLC26A01               RTALLARIGDTAFYEDATEFEGLVPEPGVRVFRFGGPLYYANKDFFLQSLYSLTGLDAGC   568
SLC26A02               KSSLLGLVEESEVFESVSAYKNLQIKPGIKIFRFVAPLYYINKECFKSALYKQTVNPILI   609
SLC26A09               NGYALAQVMDTDIYVNPKTYNRAQDIQGIKIITYCSPLYFANSEIFRQKVIAKTGMDPQK   560
SLC26A05               SYKVLGKLPETDVYIDIDAYEEVKEIPGIKIFQINAPIYYANSDLYSNALKRKTGVNPAV   566
SLC26A06               HYSVLGQVPDTDIYRDVAEYSEAKEVRGVKVFRSSATVYFANAEFYSDALKQRCGVDVDF   571
SLC26A03               KCSTLANIGRTNIYKNKKDYYDMYEPEGVKIFRCPSPIYFANIGFFRRKLIDAVGFSPLR   566
SLC26A04               SWNGLGSIPSTDIYKSTKNYKNIEEPQGVKILRFSSPIFYGNVDGFKKCIKSTVGFDAIR   576
SLC26A07               MTVSIKNMKEME--FKVKTEMDSETLQQVKIISINNPLVFLNAKKFYTDLMNMIQKENAC   533
SLC26A10_[pseudo]      KE---------------------TSKPDGPMVA---------------------------   459
                                                     :.
```

```
SLC26A11                  ------------------------------------------------------------   502
SLC26A08                  LKEEEIFSLFNSSDTNLQGGKICRCFCNCDDLEPLPRILYTERFENKLDPEASSINLIHC   644
SLC26A01                  MAARRKEG---GSE-T---------------G---------------------------V   582
SLC26A02                  KVAWKKAAKRKIKE-K---------------V---------------------------V   626
SLC26A09                  VLLAKQKYLKKQEKRR---------------MRPTQ-------QRRSLFMKTK-----TV   593
SLC26A05                  IMGARRKAMRKYAKEV---------------GN------------ANMA------NATVV   593
SLC26A06                  LISQKKKLLKKQEQLK---------------LKQLQ-------KEEKLRKQAASPKGASV   609
SLC26A03                  ILRKRNKALRKIRK--------------------LQ-------KQGLL---QVTPKGFIC   596
SLC26A04                  VYNKRLKALRKIQK--------------------LI-------KSGQL---RATKNGIIS   606
SLC26A07                  -----NQPLDDISK---------------------------------------------C   543
SLC26A10_[pseudo]         ------------------------------------------------------------   459

SLC26A11                  ------------------------------------------------------------   502
SLC26A08                  SHFESMNTS------QTASEDQVPYTVSSV-------S--QKNQGQQYEEVEEVWLPNNS   689
SLC26A01                  GEGGPAQGE-----------DLG--------------------------------PVST-   598
SLC26A02                  TLGGIQDE--------------------------------------------------M-   635
SLC26A09                  SLQELQQDFENAPPTDP-NNNQTPANGTSVSYITFSPDSSSPAQSEPPASAEAPGEPSD-   651
SLC26A05                  KADAEVDGEDA---TKP-EEEDG-----EVKYPPIVIK---------------STFPEE-   628
SLC26A06                  SINV-NTSL-----EDMRSNNVE-----DCKMMQVSSGDKMEDATANGQEDSKAPDGST-   657
SLC26A03                  TVDTIKDSD-----EEL-DNNQI-----EVLDQPINTTDLP---------FHIDWNDDL-   635
SLC26A04                  DAVSTNNAF------EP-DEDIE-----DLEELDIPTKEIE---------IQVDWNSEL-   644
SLC26A07                  EQNTLLNSLSN--------GNC------------------------------------N-   558
SLC26A10_[pseudo]         ------------------------------------------------------------   459

SLC26A11                  -----------------------SRALEVSPPRCLVLECTHVCSIDYTVVLGLGEL----   535
SLC26A08                  SRNSSPGLPDVAESQGRRSLIPYSDASLLPSVHTIILDFSMVHYVDSRGLVVLRQI----   745
SLC26A01                  ---------------------RAALVPAAAGFHTVVIDCAPLLFLDAAGVSTLQDL----   633
SLC26A02                  ---------------------SVQLSHDPLELHTIVIDCSAIQFLDTAGIHTLKEV----   670
SLC26A09                  ---------------------MLASVPPFVTFHTLILDMSGVSFVDLMGIKALAKL----   686
SLC26A05                  ---------------------MQRFMPPGDNVHTVILDFTQVNFIDSVGVKTLAGI----   663
SLC26A06                  ---------------------LKALGLPQPDFHSLILDLGALSFVDTVCLKSLKNI----   692
SLC26A03                  ---------------------PLNIEVPKISLHSLILDFSAVSFLDVSSVRGLKSI----   670
SLC26A04                  ---------------------PVKVNVPKVPIHSLVLDCGAISFLDVVGVRSLRVI----   679
SLC26A07                  ---------------------EEASQSCPNEKCYLILDCSGFTFFDYSGVSMLVEV----   593
SLC26A10_[pseudo]         ---------------------------VAEPVRVVVLDFSGVTFADAAGAREVVQVRERL   492
                                                            ::::   .   *      :  :

SLC26A11                  LQDFQKQGVALAFVGLQVPVLRVLLSADLKG---------FQYFSTLEEAEKHLRQEP--   584
SLC26A08                  CNAFQNANILILIAGCHSSIVRAFERNDFFDAG----ITKTQLFLSVHDAVLFALSRKVI   801
SLC26A01                  RRDYGALGISLLLACCSPPVRDILSRGGFLGEGPGDTAEEEQLFLSVHDAVQTARARHRE   693
SLC26A02                  RRDYEAIGIQVLLAQCNPTVRDSLTNGEYCKKE-----EENLLFYSVYEAMAFAEVSKNQ   725
SLC26A09                  SSTYGKIGVKVFLVNIHAQVYNDISHGGVFEDGS---LECKHVFPSIHDAVLFAQANARD   743
SLC26A05                  VKEYGDVGIYVYLAGCSAQVVNDLTRNRFFE-NP---ALWELLFHSIHDAVLGSQLREAL   719
SLC26A06                  FHDFREIEVEVYMAACHSPVVSQLEAGHFFD-AS---ITKKHLFASVHDAVTFALQHPRP   748
SLC26A03                  LQEFIRIKVDVYIVGTDDDFIEKLNRYEFFD-GE---VKSSIFFLTIHDAVLHILMKKDY   726
SLC26A04                  VKEFQRIDVNVYFASLQDYVIEKLEQCGFFD-DN---IRKDTFFLTVHDAILYLQNQVK-   734
SLC26A07                  YMDCKGRSVDVLLAHCTASLIKAMTYYGNLDSE-----KPIF-FESVSAAI-SHIHSN-K   645
SLC26A10_[pseudo]         ASRCRDARIRLLLAQCNALVQGTLTRVGLLDRV-----TPDQLFVSVQDAAAYALGSLLR   547
                                  : : :.     .   :                   * ::  *

SLC26A11                  GT----------------QPYNIRED---SILDQKVALLKA-------------------   606
SLC26A08                  GSSELSIDESETV---IRETYSETDKNDNSRYKMSSSFLGSQKNVSPGFIKIQQPVEEES   858
SLC26A01                  LEAT-DAHL---------------------------------------------------   701
SLC26A02                  KGVCVPNGLSLSSD----------------------------------------------   739
SLC26A09                  VTPGHNFQGA---P----------GDAELSLYDSEEDI--------------------RS   770
SLC26A05                  AEQEASAPPSQEDLEPNATP-ATPEA----------------------------------   744
SLC26A06                  VPD---------SPVSVTRL----------------------------------------   759
SLC26A03                  STSK--FNPSQEKDGKIDFTINTNGGLRNRVYEVPV----------------------ET   762
SLC26A04                  ---------SQEGQGSILETITLIQDCKDTLELIETEL-------------------TEE   766
SLC26A07                  NLSKL--SDHSEV-----------------------------------------------   656
SLC26A10_[pseudo]         GSSTR--SGSQEALGCGK------------------------------------------   563

SLC26A11                  ------------------------------------------------------------   606
SLC26A08                  ELDLELESEQEAGLGLDLDLDRELEPEMEPKAETETKTQTEMEPQPETEPEMEPNPKSRP   918
SLC26A01                  ------------------------------------------------------------   701
SLC26A02                  ------------------------------------------------------------   739
SLC26A09                  YWDLEQE----------------MFGSMF---HAETLTAL--------------------   791
SLC26A05                  ------------------------------------------------------------   744
SLC26A06                  ------------------------------------------------------------   759
SLC26A03                  KF----------------------------------------------------------   764
SLC26A04                  ELDVQDE----------------AMRTLA---S---------------------------   780
SLC26A07                  ------------------------------------------------------------   656
SLC26A10_[pseudo]         ------------------------------------------------------------   563
```

```
SLC26A11              ---------------------------------------------------- 606
SLC26A08              RAHTFPQQRYWPMYHPSMASTQSQTQTRTWSVERRRHPMDSYSPEGNSNEDV 970
SLC26A01              ---------------------------------------------------- 701
SLC26A02              ---------------------------------------------------- 739
SLC26A09              ---------------------------------------------------- 791
SLC26A05              ---------------------------------------------------- 744
SLC26A06              ---------------------------------------------------- 759
SLC26A03              ---------------------------------------------------- 764
SLC26A04              ---------------------------------------------------- 780
SLC26A07              ---------------------------------------------------- 656
SLC26A10_[pseudo]     ---------------------------------------------------- 563
```

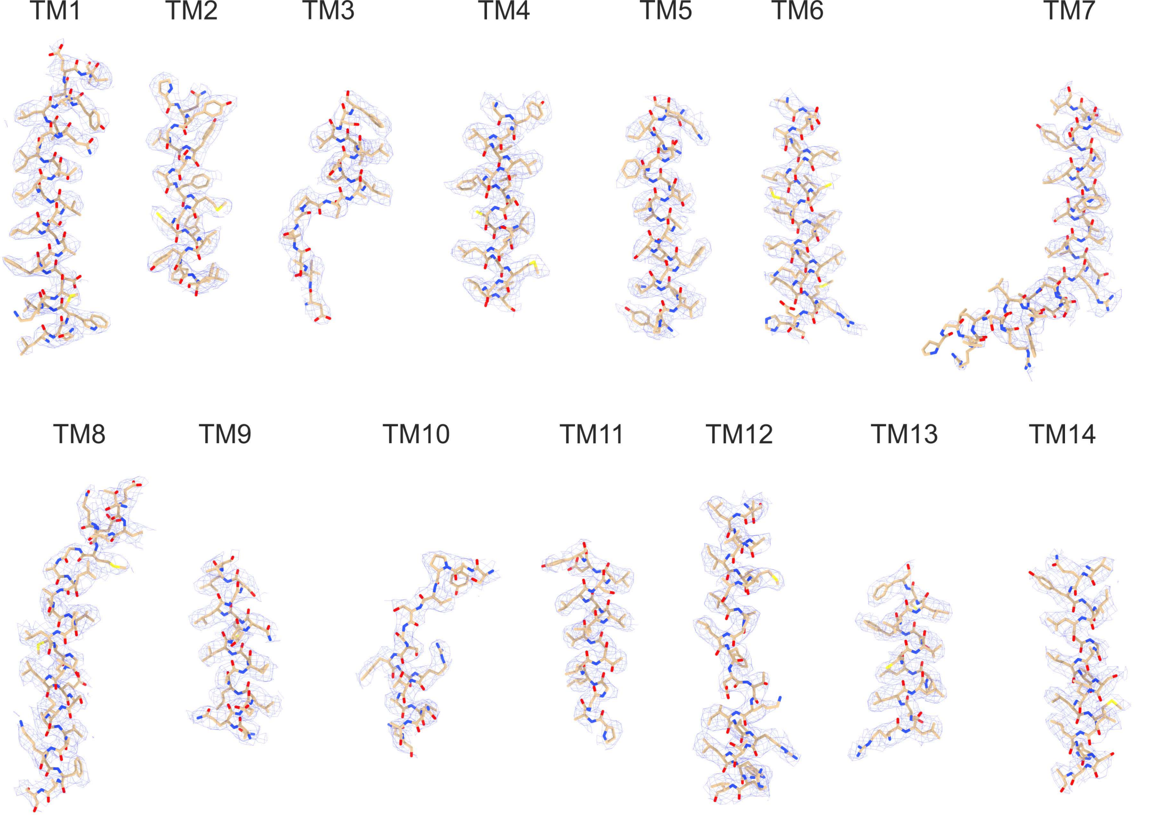

Supplementary Figure 6: Representative densities of SLC26A11-Nb11. Transmembrane segments shown as sticks with corresponding density (blue mesh).

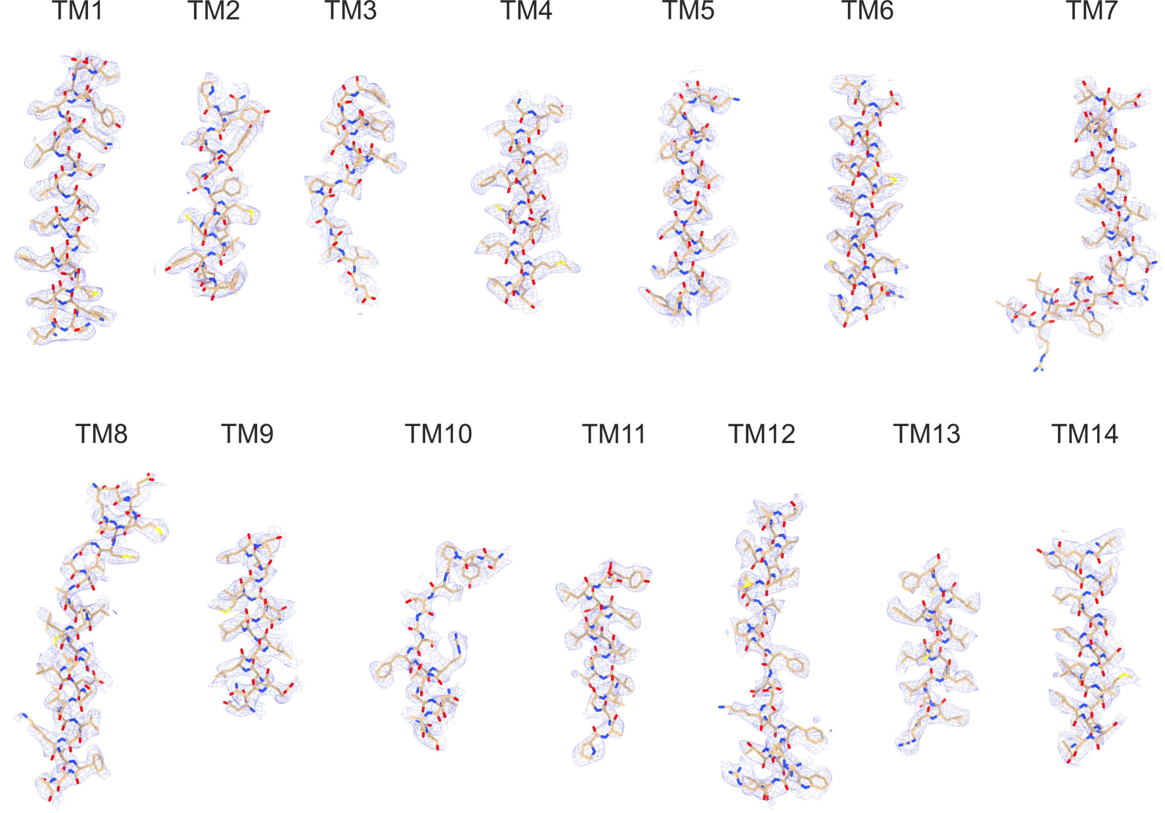

Supplementary Figure 7: Representative densities of SLC26A11-Nb4. Transmembrane segments shown as sticks with corresponding density (blue mesh).

Nb4 dataset: Map in Paper

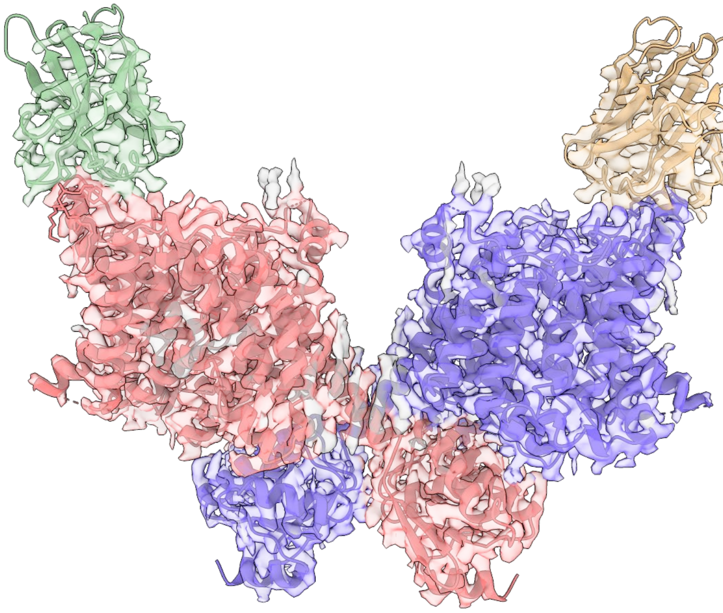

C2 refine in CryoSPARC
210’771 particles 2.8 Å

Nb4 dataset Refinement tree

C2 refine in CryoSPARC

↓

C1 refine in RELION-5.0

↓

C2 refine in RELION-5.0

↓

Symmetry expand

↓

C1 Local refine

↓

Unaligned 3D Classification

↓

C1 Local refine

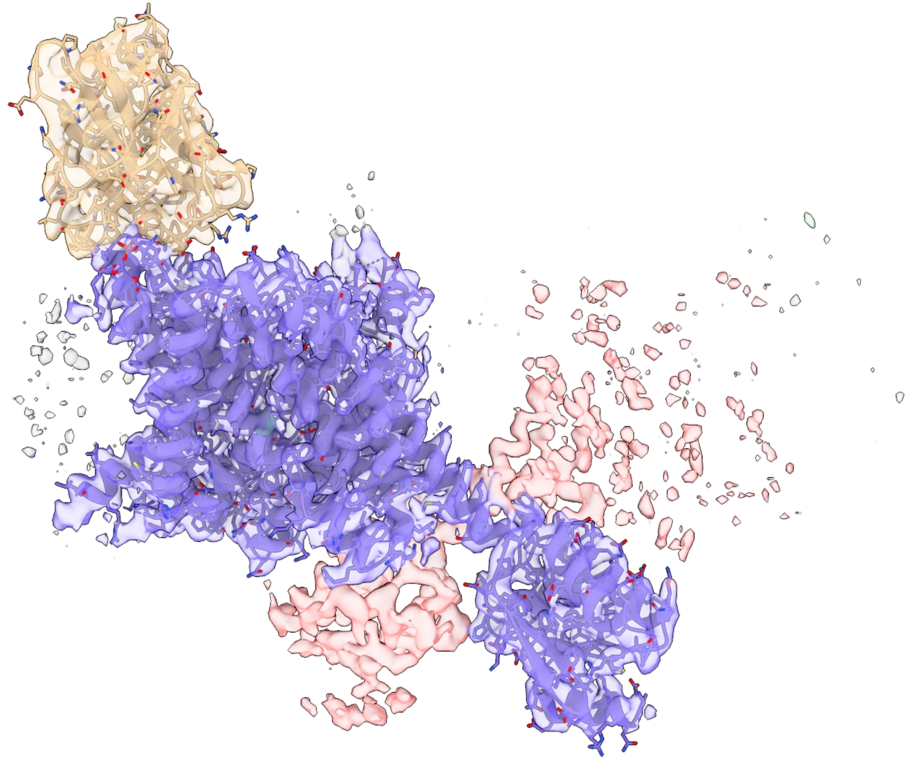

3.1 Å with 72’045 particles

Nb4 dataset side by side

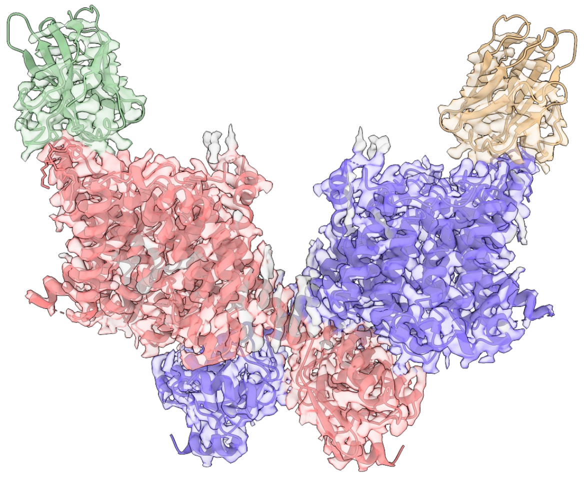

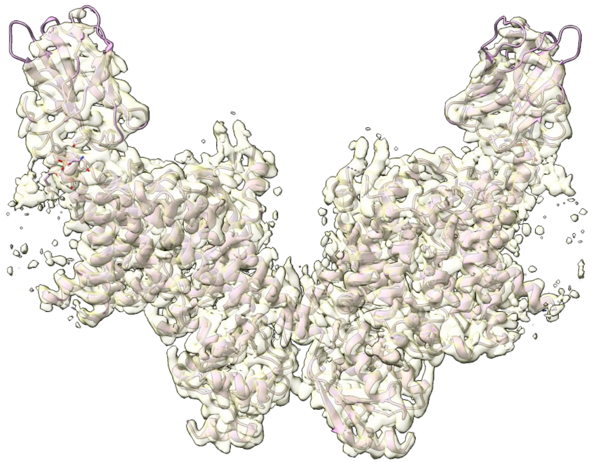

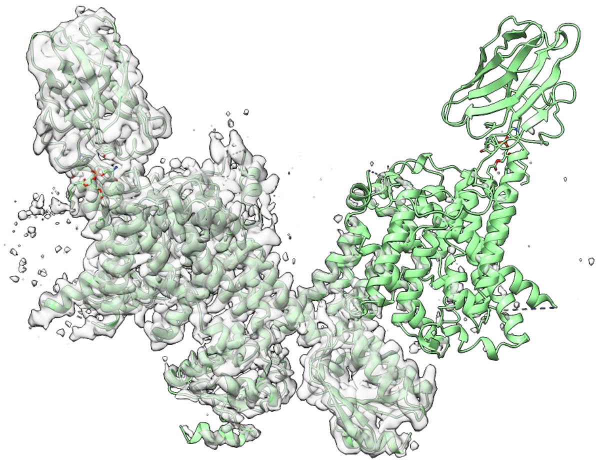

C2 refine in CryoSPARC
(Map in paper)

2.8 Å with 210’771
particles

C2 refine in RELION-5.0

3 Å with 210’775
particles

Symmetry expansion +
local refine (reviewer
suggestion)

3.1 Å with 72’045 particles

Nb4 dataset volume comparison

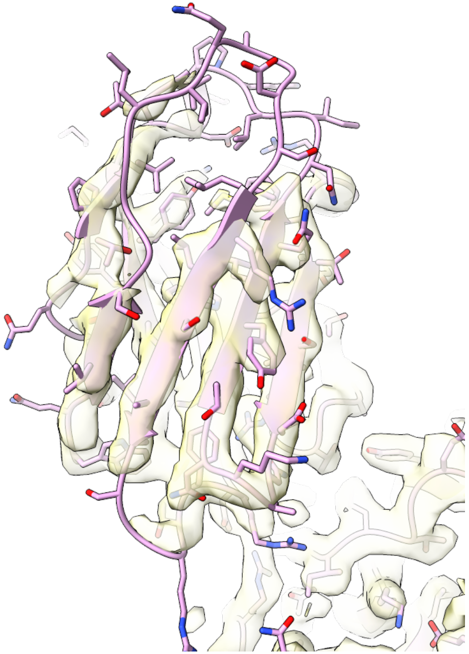

C2 refine in RELION-5.0

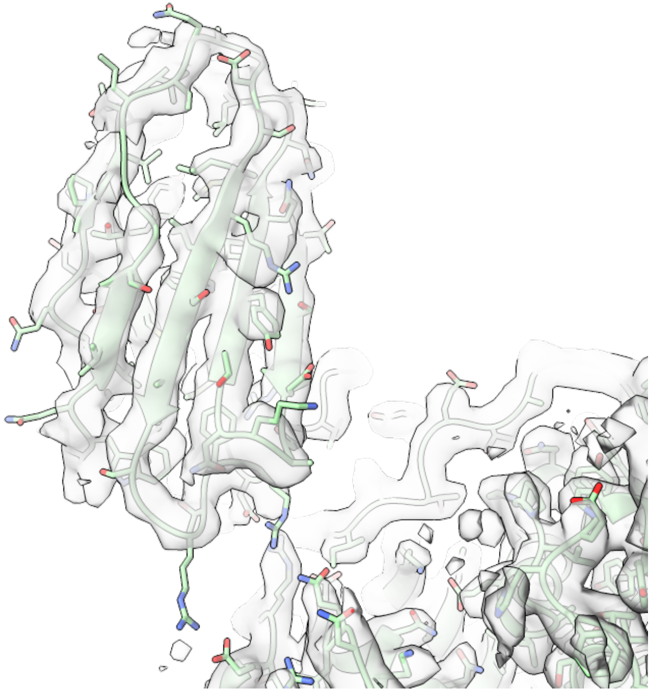

symmetry expansion + local refine
(reviewer suggestion)

Nb4 dataset volume comparison

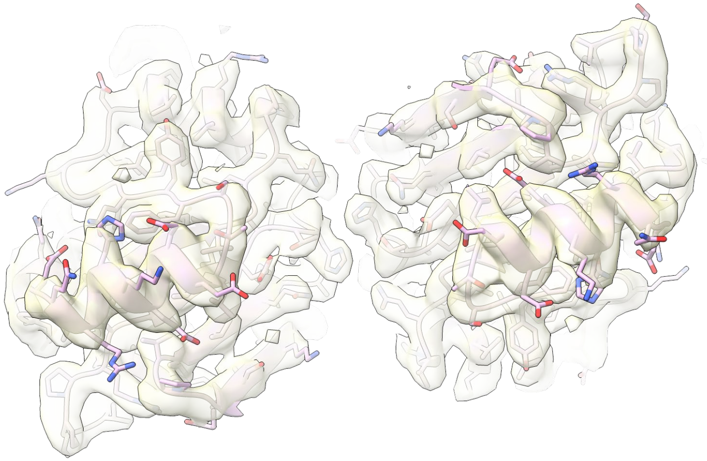

C2 refine in RELION-5.0

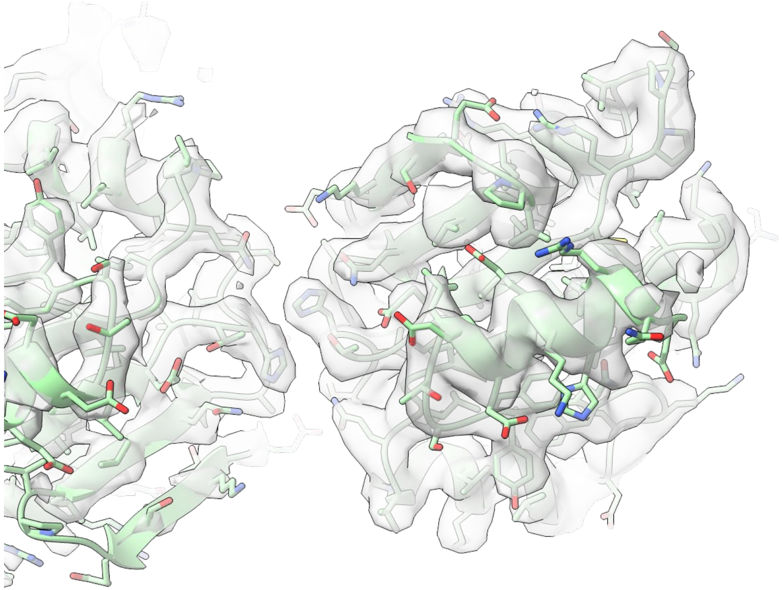

Symmetry expansion + local refine
(reviewer suggestion)

Nb11 dataset: Map in Paper

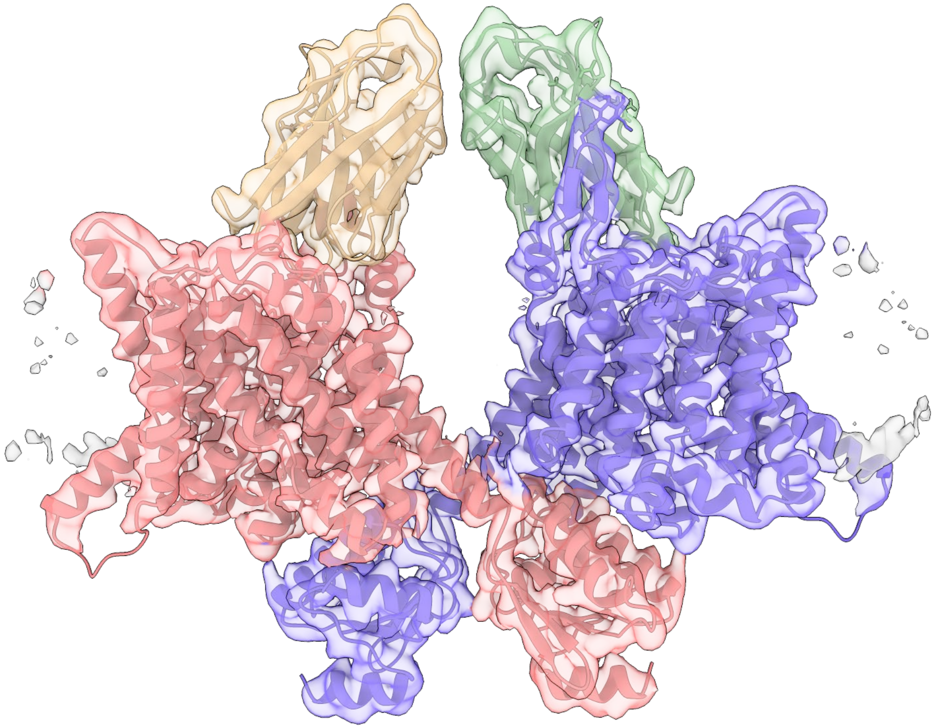

C2 refine in CryoSPARC
48’803 particles, 3.2 Å

Nb11 dataset Refinement tree

C2 refine in CryoSPARC

↓

Expand Box size to 400 pix

↓

C2 refine in CryoSPARC w/CTFRefine

↓

C1 refine in RELION-5.0

↓

C2 refine in RELION-5.0

↓

Symmetry expand

↓

C1 Local refine

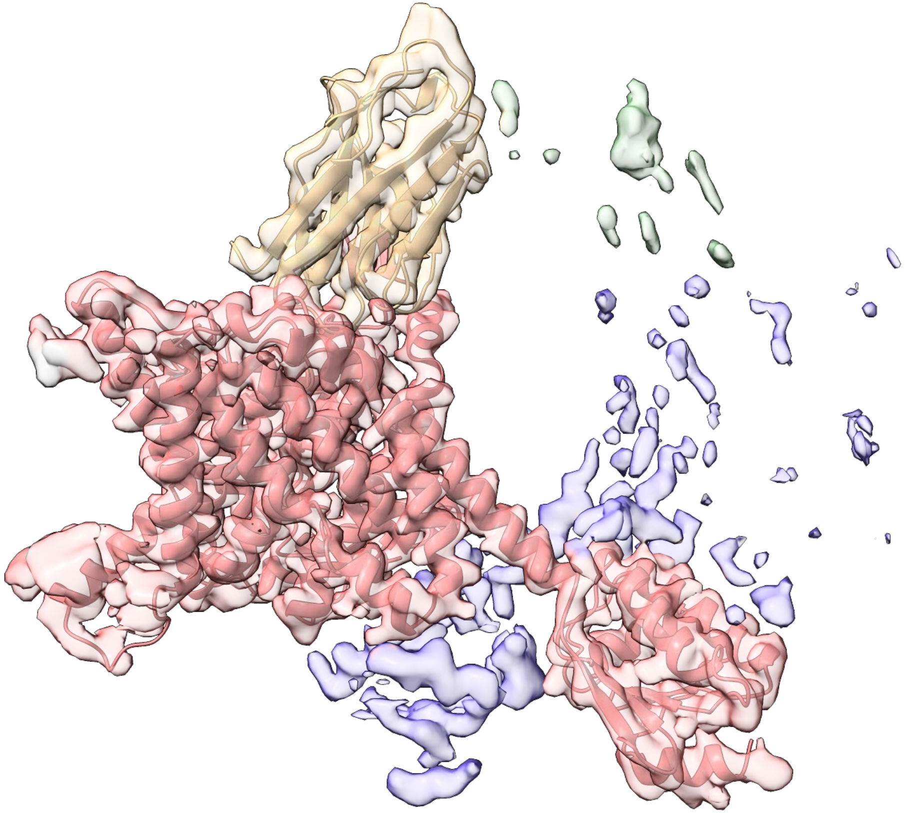

48’803 particles, 3.3 Å

Nb11 dataset comparison

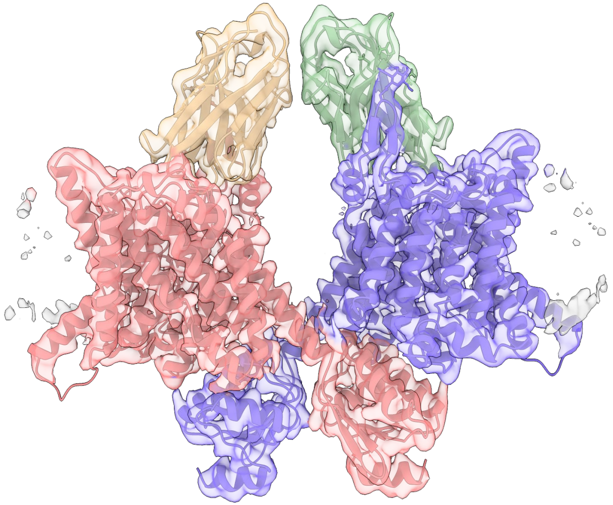

C2 refine in CryoSPARC
(Map in paper)

48’803 particles, 3.2 Å

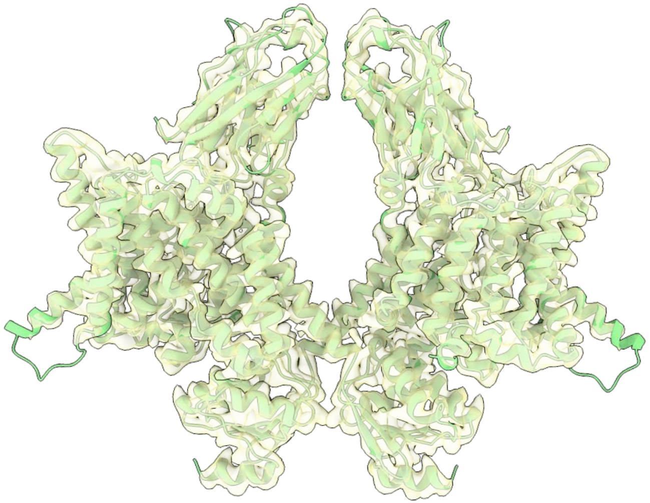

C2 refine in RELION-5.0

48’803 particles, 3.3 Å

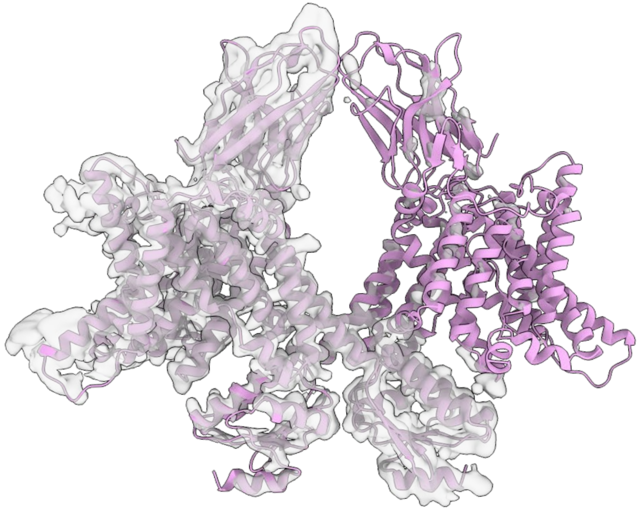

C2 symmetry expansion + local refine
(reviewer suggestion)

48’803 particles, 3.3 Å

Reviewer comment

Author feedback

Original text

Revised text (first revision)

Revised text (second revision)

REVIEWER COMMENTS – second round

Reviewer #1 (Remarks to the Author):

The authors have address my comments.

We thank Reviewer #1 for this positive feedback.

Reviewer #2 (Remarks to the Author):

The revised version of the manuscript satisfactorily addresses my comments.

We thank Reviewer #2 for this positive feedback.

Reviewer #3 (Remarks to the Author):

The authors have addressed most of my comments and clarified several points that have improved the manuscript significantly.

We thank Reviewer #3 for this positive feedback.

I am still not overly convinced of the electroneutral transport mechanism interpretation. As the data is not clear cut (as stated by the authors), I suggest initial rates of uptake for this protein (SLC26A11), in this system, could be informative and back up the authors interpretation.

We feel that the reviewer's statement suggesting that we do not find the data "clear cut" is formulated too strong. We have stated that the 30% reduction observed when imposing a negative-inside membrane potential is *difficult to interpret*. It is *difficult to interpret* as the reduction in uptake observed for a negative-inside potential is: 1) too modest to be explained by electrogenic transport; and 2) not matched with an opposite effect for a positive-inside membrane potential.

Following extensive discussions with our colleagues, we concluded that the 30% reduction in accumulation at a negative-inside membrane potential is best explained by a combination of slow passive proton leakage and comparably low buffer capacity. In addition to the applied pH gradient, a negative-inside membrane potential provides an additional driving force for proton uptake. In our experimental setup, at an internal pH of 7.5, 20 mM Hepes is effectively the only buffer component. Slow passive influx of protons may therefore lead to a reduction in the internal pH, thereby impacting the magnitude of the pH gradient and the final sulfate accumulation level. Since a membrane potential is generated instantaneously while dissipation of a proton gradient by passive proton leakage is comparably slow, the latter's impact would be most pronounced at later time points. We therefore appreciate the reviewer's suggestion to measure initial transport rates under these conditions. However, regrettably, we do not currently have the resources to perform this experiment in a timely fashion (as explained in more detail to the editor). Instead, we will follow the editor's suggestion and tone down and appropriately caveat our interpretation.

As our analysis of the stoichiometry of the transported ions involves two reciprocal experiments of which one (positive-inside membrane potential) is in agreement with electroneutrality, while the other (negative-inside membrane potential) resists simple interpretation, but is in principle not in disagreement with electroneutrality, we have toned down our claim to say that "the overall pattern observed is broadly consistent with an electroneutral transport mode". In addition, we have softened or redrafted each subsequent statement on electroneutrality. Please find these changes below.

Line 122-133:

We observed only modest changes when generating a membrane potential of 0 and +60 mV, but at -60 mV sulfate accumulation was reduced by approximately 30%. For electrogenic transport, a more pronounced reduction at -60 mV and a concomitant increase in sulfate accumulation at +60 mV would be expected, as shown for the proton:fumarate symporter SLC26Dg [13]. Though the reduced accumulation levels at -60 mV resist simple interpretation, ~~these results together support~~ the overall pattern observed is broadly consistent with an electroneutral transport mode for SLC26A11. In the most parsimonious model, SLC26A11 is an exchanger that couples the symport of one proton and one sulfate ion (net charge of -1) to the antiport of one chloride ion (net charge of -1).

Line 253-258:

*Finally, we did not observe any (cis) proton- and (trans) chloride-driven sulfate accumulation in SLC26A11$^{\Delta C}$(E320Q) proteoliposomes **[Fig. 4F]**. This is consistent with the mutant's very high, pH-independent sulfate binding affinity, which likely results in a high prevalence of the sulfate-bound state in both compartments. Furthermore, any alteration in the ~~electroneutrality of transport resulting from~~ stoichiometry of the transported ions due to the E320Q mutation could generate an inhibitory membrane potential, thereby reducing sulfate uptake in the liposomal lumen further.*

Line 285-294

*Given the impact of Glu-320 protonation on substrate selectivity and transport, we examined the consequences of the E320Q mutation on SLC26A11-specific whole-cell currents. SLC26A11(E320Q) showed similar expression levels and intracellular distribution as the wild type protein **[Supplementary Fig. 15]**. However, whereas SLC26A11(WT)-specific currents were activated by external acidification at high external sulfate, SLC26A11(E320Q) anion currents instead remained low but exceeded background currents **[Fig. 5D-F, Supplementary Fig. 19]**. The lack of pH-dependence observed for SLC26A11(E320Q) chloride currents suggests that activation of the chloride channel requires the protein to cycle through different Glu-320 protonation states, as previously observed for the ~~electroneutral~~ proton:sulfate/chloride exchange mode of transport **[Fig. 4F]**.*

Line 295-299

*Taken together, our findings ~~indicate~~ suggest that SLC26A11 exhibits two distinct transport functions: electroneutral proton:sulfate/chloride exchange and chloride channel activity. In conjunction with the sulfate dependency of chloride channel activation, the distinct pH dependence of the currents recorded with internal **[Supplementary Fig. 17]** or external sulfate **[Fig. 5A-C]** demonstrate that SLC26A11 chloride channels are gated by SLC26A11 sulfate transport.*

Line 309-317

*Our in vitro transport studies using membrane-reconstituted protein ~~indicate~~ suggest that SLC26A11 operates as a electroneutral proton:sulfate/chloride exchanger **[Fig.1]**. SLC26A11 thus closely resembles the function of the rat lysosomal sulfate transporter reported three decades ago [42]. Together with its lysosomal localization [26,30] and its upregulation during lysosomal biogenesis [27], our functional characterization suggests that SLC26A11 represents the elusive lysosomal transporter for sulfate. Its use of the proton gradient as driving force to export sulfate from the lysosome is consistent with other lysosomal transporters [37], e.g., SLC17A5 [55], SLC36A1 [56], SLC46A3 [57], SLC63A1 [58], and SLC66A4 [59]. In agreement with its role as a housekeeping protein, SLC26A11 shows a broad tissue distribution in the human body [6].*
